# FlexStat: combinatory differentially expressed protein extraction

**DOI:** 10.1093/bioadv/vbae056

**Published:** 2024-04-11

**Authors:** Senuri De Silva, Asfa Alli-Shaik, Jayantha Gunaratne

**Affiliations:** Translational Biomedical Proteomics Laboratory, Institute of Molecular and Cell Biology (IMCB), Agency for Science, Technology and Research (A*STAR), Singapore 138673, Singapore; Department of Anatomy, Yong Loo Lin School of Medicine, National University of Singapore, Singapore 117594, Singapore; Translational Biomedical Proteomics Laboratory, Institute of Molecular and Cell Biology (IMCB), Agency for Science, Technology and Research (A*STAR), Singapore 138673, Singapore; Translational Biomedical Proteomics Laboratory, Institute of Molecular and Cell Biology (IMCB), Agency for Science, Technology and Research (A*STAR), Singapore 138673, Singapore; Department of Anatomy, Yong Loo Lin School of Medicine, National University of Singapore, Singapore 117594, Singapore

## Abstract

**Motivation:**

Mass spectrometry-based system proteomics allows identification of dysregulated protein hubs and associated disease-related features. Obtaining differentially expressed proteins (DEPs) is the most important step of downstream bioinformatics analysis. However, the extraction of statistically significant DEPs from datasets with multiple experimental conditions or disease types through currently available tools remains a laborious task. More often such an analysis requires considerable bioinformatics expertise, making it inaccessible to researchers with limited computational analytics experience.

**Results:**

To uncover the differences among the many conditions within the data in a user-friendly manner, here we introduce FlexStat, a web-based interface that extracts DEPs through combinatory analysis. This tool accepts a protein expression matrix as input and systematically generates DEP results for every conceivable combination of various experimental conditions or disease types. FlexStat includes a suite of robust statistical tools for data preprocessing, in addition to DEP extraction, and publication-ready visualization, which are built on established R scientific libraries in an automated manner. This analytics suite was validated in diverse public proteomic datasets to showcase its high performance of rapid and simultaneous pairwise comparisons of comprehensive datasets.

**Availability and implementation:**

FlexStat is implemented in R and is freely available at https://jglab.shinyapps.io/flexstatv1-pipeline-only/. The source code is accessible at https://github.com/kts-desilva/FlexStat/tree/main.

## 1 Introduction

**Figure 1.**
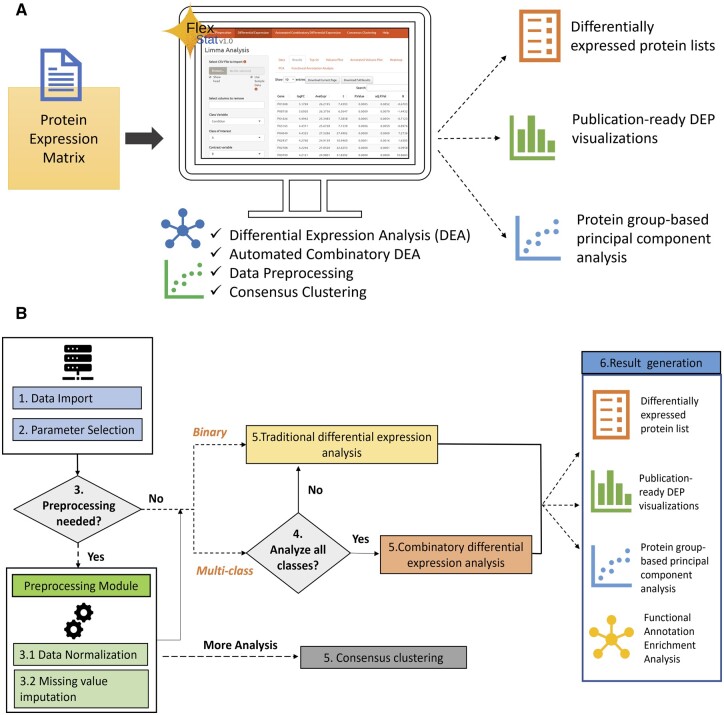
Overview of FlexStat and its ecosystem. (A) FlexStat leverages on well-established R packages. It accommodates CSV files containing protein expression matrices from various downstream software tools. FlexStat is accessible through a user-friendly GUI on shinyapps, offering both flexibility and simplicity. With a robust suite of scientific computing packages, including Limma and Plotly, as well as EnhancedVolcano and other complementary tools, FlexStat ensures efficient data processing, analysis, and visualization. (B) Symbolic visualization of FlexStat’s workflow. The stepwise process includes data preprocessing, combinatory and user-defined conditions-based DEA, and comprehensive functionalities with visualization for informed data interpretation.

Mass spectrometry (MS)-based proteomics has emerged as a robust technology to efficiently identify and quantify proteins across diverse conditions, enabling comprehensive exploration of molecular functions and cellular changes (Thompson *et al.* 2003, Cox *et al.* 2014, Aebersold and Mann 2016, Zecha *et al.* 2019). Proteomics search engines produce tables with protein identities and abundances, which are used as input files for extracting differentially expressed proteins (DEPs). Several downstream proteomic analysis tools offer DEP functionalities and capabilities. Currently available standalone statistical tools such as Perseus (Tyanova *et al.* 2016) and MSPypeline (Heming *et al.* 2022) have proven to be invaluable comprehensive data analysis solutions. Moreover, the Shiny framework facilitates easy access to statistical applications, offering simpler interfaces and data visualization. However, these tools are commonly tailored to specific data types. For example, LFQ-analyst (Shah *et al.* 2020), ProtExA (Minadakis *et al.* 2020), and StatsPro (Yang *et al.* 2022) cater specifically to label-free data analysis. ProVision (Gallant *et al.* 2020), Protigy (https://github.com/broadinstitute/protigy), and Fragpipe-Analyst (Hsiao *et al.* 2024)  are used for analyzing outputs from widely used raw data processing software such as MaxQuant, Spectralmill, and Fragpipe (https://github.com/MonashProteomics/FragPipe-Analyst). Tools such as Amica (Didusch *et al.* 2022) and MSStatShiny (Kohler *et al.* 2023) are primarily amenable for DEP analysis between user-defined comparison conditions in a low throughput manner. While all the above tools streamline proteomic downstream analysis workflows, they often require considerable bioinformatics expertise and may pose accessibility challenges for beginners and researchers with limited experience in this area. This underscores the need for a user-friendly tool that efficiently extracts DEPs from generic input formats, while also providing in-depth biological insights from quantitative proteomics data derived from multiple conditions. To address all these challenges, we developed an easy-to-use combinatory-based differential expression analysis (DEA) tool, FlexStat—an R shiny web application. It can seamlessly analyze proteome differences in multiple conditions by automatically generating combinatory pairwise comparisons yielding DEA results in minutes. FlexStat accepts a simpler input format and is equipped with comprehensive data preprocessing steps including missing value imputation and normalization algorithms. It enables DEA in both automated and user-defined combinatory manners (Fig. 1). The results generated by FlexStat include downloadable preprocessed DEP result tables, and publication-ready visualization including box plots, volcano plots, and scatter plots. FlexStat is freely accessible at https://jglab.shinyapps.io/flexstatv1-pipeline-only

## 2 Methods

### 2.1 Input data format

FlexStat simplifies input requirements by employing a straightforward data format, which mandates only an expression matrix with condition/class assignments. The necessary data format illustrated by an example dataset is readily available on the front page of each FlexStat function.

### 2.2 Data preprocessing

Data preprocessing is a vital step in transforming raw data into a usable and comparable format. FlexStat facilitates this process by supporting data transformation, normalization, and imputation as the primary preprocessing steps. In FlexStat, logarithmic transformations, with bases two and ten, are offered. FlexStat presents commonly employed data normalization methods of median, quantile, internal reference scaling (iRS), and variance stabilization normalization (VSN). Median normalization is well-suited for scenarios where the majority of proteins are assumed to remain unchanged across samples, establishing the median protein abundance as a stable reference point (Välikangas *et al.* 2018). FlexStat implements median normalization using the limma R package (Ritchie *et al.* 2015). Quantile normalization ensures a uniform distribution of protein abundances across all samples, which is helpful when comparing samples with disparate distributions (Välikangas *et al.* 2018, Zhao *et al.* 2020). FlexStat utilizes quantile normalization through the “normalize.quantiles” function from the R/Bioconductor package “preprocessor” (Bolstad 2014). iRS is a specialized normalization method used for label-based quantified data, such as TMT, and iTRAQ, correcting for random MS2 sampling that occurs between experiments (Plubell *et al.* 2017). VSN is a statistical method designed to update sample variances independent of their mean intensities, thereby harmonizing samples onto a common scale more suitable for label-free proteomics data (Välikangas *et al.* 2018). FlexStat implements VSN using the vsn2 R package (Huber *et al.* 2002).

Data imputation involves estimating missing or incomplete data points within a dataset using statistical techniques. FlexStat allows users to impute missing values through random draws from a normal distribution (Yanovich *et al.* 2018, Jin *et al.* 2021), k-nearest neighbor (Guo *et al.* 2015), and missForest (Mertins *et al.* 2016) algorithms. The normalized and imputed data generated by FlexStat are available as downloadable files for downstream analysis. Each preprocessing step is complemented by quality control plots, including boxplots and density plots illustrating sample distribution, along with a principal component analysis (PCA) plot displaying how data preprocessing influences the biological condition.

### 2.3 Combinatory statistical analysis

The current iteration of FlexStat relies on limma (Ritchie *et al.* 2015) DEA method to automatically generate combinations of selected covariates, including all pairwise and grouped comparisons specified by the user. The limma workflow is a robust and widely used approach for analyzing gene expression data, aiding in the identification of genes associated with specific biological processes or conditions. FlexStat’s efficiency is optimized to ensure swift execution, such that the execution time is proportional to the number of unique conditions of the covariate, leading to a time complexity of O(*n*^2^), where *n* is the number of unique conditions.

In traditional limma analysis, contrasts are defined, the design matrix is generated, the linear model is fitted for each protein, coefficients and standard errors are estimated for defined contrasts, and *t*-statistics and *P*-values are computed using empirical Bayes moderation. Results are sorted and filtered based on specified cutoffs for log fold changes and *P*-values corrected for multiple tests. In FlexStat’s DEA feature on user-defined comparison conditions, the design matrix is constructed based on the input class variable, where the contrasts are defined as the class of interest compared to the contrast class. When the “Contrast other classes” flag is activated, all classes/conditions except the one of interest are labeled as “Others.”

In FlexStat’s combinatory DEA feature, all possible pairwise combinations are defined in two stages. Firstly, all possible pairwise combinations are created considering each class individually using the “comb” function in the “utils” package. Secondly, FlexStat groups multiple classes together which are in the same combination generated in the previous step, and effectively creates pairs for comparisons. This function utilizes the “starts” function in the “partitions” package, known as a modified version of the original enumeration function of set partitions introduced in the listParts function (Hankin 2006), where a partition of a set of unique class *S* = {1, …., *n*} is a family of sets of *T*_1_, …, *T_k_* satisfying,

-
$i\;\neq \;j\;\to \;T_{i}\;\cap \;T_{j}\;=\;\emptyset$ .
-
${⋃}_{i=1}^{k}\;T_{k}=\;Ss$

-
$T_{i}\;\neq \;\emptyset \;\mathrm{for}\;i\;=\;1\ldots ,\;k$

-
$1<\;|T_{i}|\;<\;|S|\;\vee (|T_{i}|\;==1\;\wedge 1<\;|T_{s}|<\;|S)\mathrm{for}\;n>2i$

The superset and sets with a cardinality of one (i.e. sets with all unique class values) are excluded. Subsequently, DEA is performed for each pair generated as described above, and the results are filtered based on the provided cutoffs, similar to a user-defined pairwise comparison. The web application was thoroughly optimized to efficiently work with large datasets with multiple conditions, delivering results within seconds of execution.

### 2.4 Visualization

FlexStat provides a user-friendly interface for data visualization of the results from DEA. Users can effortlessly generate various publication-ready plots to depict the DEA results. These visualizations are generated in real time and accompany the analysis results. The available visualization plots include heat maps, box plots highlighting the top fifty DEPs, PCA plots, and volcano plots. Users have the option to visualize and download separate boxplots for the top-up and down-regulated proteins. Additionally, PCA plots for each protein group mentioned above are available for download, allowing users to derive more meaningful interpretations of the data. Furthermore, the volcano plots provide customization options, allowing users to define cutoff values for log fold changes and *P*-values based on their preferences.

### 2.5 Data download

FlexStat provides users with convenient options to download protein matrices at various stages of processing and analysis. The preprocessing results, including normalization and imputation, are accessible as downloadable comma-separated files. Users can easily download the results of DEA, which include filtered DEA results on user-defined comparison conditions. Additionally, the resulting matrices from automated combinatory DEA are organized into sheets, where each sheet represents a pairwise comparison. FlexStat platform also facilitates the downloading of visualizations such as volcano plots, heatmaps, and PCA plots, enabling users to enhance their data analysis effortlessly.

### 2.6 Functional protein classes-based analysis

FlexStat offers users with convenient options to download protein matrices at Functional protein classes-based analysis in disease conditions that provide important implications compared to studying the proteome alone. Proteases, kinases, and transcription factors represent the most common and widely interrogated functional categories that play pivotal roles in disease therapeutics. For instance, kinases are often targeted by common anti-cancer drugs (Shawver *et al.* 2002, Bhullar *et al.* 2018) while proteases and transcription factors are emerging as new therapeutic targets (Kosok *et al.* 2020, Alli-Shaik *et al.* 2022). GO database is a useful resource for deriving the protein lists for these functional protein classes. However, utilizing the GO database is not straightforward for visualizing differences in different disease conditions. Therefore, in the FlexStat web application, functional protein classes-based PCA and visualization are supported with proteases (http://degradome.uniovi.es/asp.html), kinases (http://www.kinhub.org/kinases.html), and transcription factors (http://humantfs.ccbr.utoronto.ca/download.php). The users can select the functional protein class of interest and conduct PCA to identify any disease-specific differences automatically.

### 2.7 Functional enrichment and protein network analysis

The functional enrichment analysis is commonly performed using widely recognized web server Database for Annotation, Visualization, and Integrated Discovery (DAVID) (https://david.ncifcrf.gov/tools.jsp) (Huang *et al.* 2009, Sherman *et al.* 2022) functional annotation analysis platform and the ShinyGO application (http://bioinformatics.sdstate.edu/go/) (Ge *et al.* 2020). These platforms offer exclusive functionalities for gene set enrichment and pathway analysis across diverse databases. Moreover, protein network analysis is implemented through the well-established online tool, STRING (https://string-db.org/) (Szklarczyk *et al.* 2023). Link to each of this platform is provided in the “Functional Annotation Analysis” section of the “Differential Expression” component.

## 3 FlexStat case studies

To showcase the functionalities of FlexStat, we tested it in our recent study of breast cancer cell lines (Kosok *et al.* 2020). FlexStat enabled a thorough downstream analysis, including combinatory, use-defined DEA across the disease groups, and produced visualization within minutes (Supplementary Fig. S1, Supplementary Data S1 and S2). Additionally, we assessed DEA performance of FlexStat using two ground truth datasets: a spiked-in proteomics dataset (Ramus *et al.* 2016) and a ubiquitin interactor affinity-enriched dataset (Zhang *et al.* 2018). This analysis unveiled a 100% overlap between the DEPs identified with the original protein lists within a few seconds (Supplementary Figs S2 and S3, Supplementary Data S3 and S4). Notably, analysis of the ubiquitin interactor dataset, comprising four conditions, was also completed within seconds (Supplementary Data S5 and S6).

Moreover, we evaluated FlexStat using two additional cancer datasets: Clear cell renal carcinoma data (Guo *et al.* 2015) (Supplementary Data S7 and S8), and the CPTAC ovarian cancer dataset (Zhang *et al.* 2016) (Supplementary Data S9 and S10). Analysis of these datasets showcased the linear execution time durations for differential expression and combinatory DEA processes. A comprehensive summary of the execution time durations for each FlexStat function on the case studies is included in Supplementary Fig. S3. Comprehensive documentation that outlines the functionalities of FlexStat is presented as tutorials (Supplementary Fig. S4). These step-by-step tutorials offer systematic guidance to learn the tool with the use of the provided example datasets and suggested analysis parameters. Alternatively, the intuitive graphical user interface ensures easy learning, assisting swift and seamless data exploration and analysis. A comparison of FlexStat's features with currently available tools is depicted in Table 1.

**Table 1.** Comparison of FlexStat’s features with available tools.^a^

	Feature^b^	Perseus	LFQ-Analyst	ProtExA	Protigy	Amica	MS Stats Shiny	FlexStat
	**Input**							
1	Generic file input	x			x	x	x	**x**
2	Easy data preparation	x					x	**x**
	**Data visualization**							
3	Multiple data visualizations (boxplot, heatmap, volcano plots)	x				x	x	**x**
4	Customizable Data visualizations	x	(x)	x	x	x	(x)	**x**
	**Differential expression (DE)**					x	x	**x**
5	Proteomic-specific differential expression	x	x			x	x	**x**
6	Multiple comparisons							
7	Grouped multiple comparisons							
	**Post DE Analysis/Other functions**							
8	Consensus clustering							**x**
9	Biological network analysis	x		x	x	x		**x**
10	Functional enrichment analysis	x		x				**x**
11	Protein subgroup-based analysis							**x**
	**Deployment**							
12	Available at public webserver		x	x	x	x	x	**x**
13	Support large proteomics datasets (coverage > 10 000 proteins)					(x)		**x**
14	Local environment deployment	x				x		**x**
	**Usability**							
15	Less complex data analysis procedure for researchers with less or no programming experience	x					x	**x**

a x, support; (x), limited/partial support.

b The main steps of the downstream proteomics data analysis workflow are highlighted.

## 4 Conclusion

We have developed an open-source package called FlexStat that can handle analysis of MS-based proteomics datasets from multiple conditions using both combinatory and user-defined conditions-based approaches. It offers a seamless experience for researchers with limited bioinformatics expertise, from data preprocessing to generating publication-ready visualizations and conducting downstream functional enrichment analysis. In all, FlexStat is an easy-to-use robust tool for comprehensive exploration of complex proteomics datasets featuring multiple conditions.

## Supplementary Material

vbae056_Supplementary_Data
