# Supplementary material for: FlexStat: combinatory differentially expressed protein extraction
Source: Bioinform Adv. 2024 Apr 11;4(1):vbae056. doi: 10.1093/bioadv/vbae056 (PMC11055397; doi:10.1093/bioadv/vbae056)
Supplement: vbae056_Supplementary_Data [file vbae056_supplementary_data.zip › Supplementary_Figures.pdf]

S1.

A.

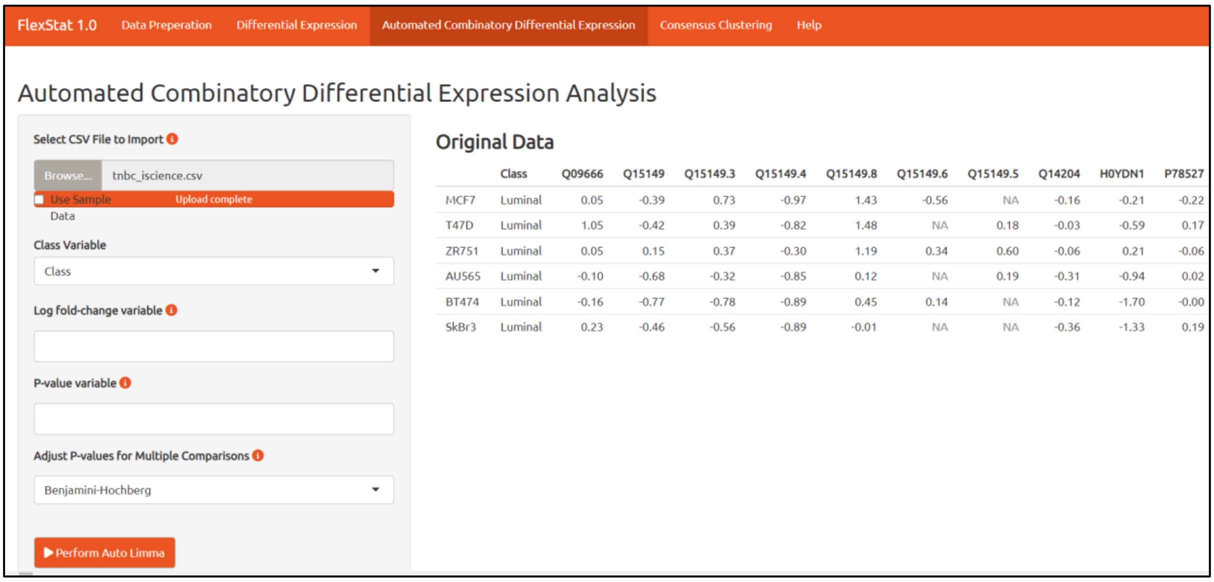

B.

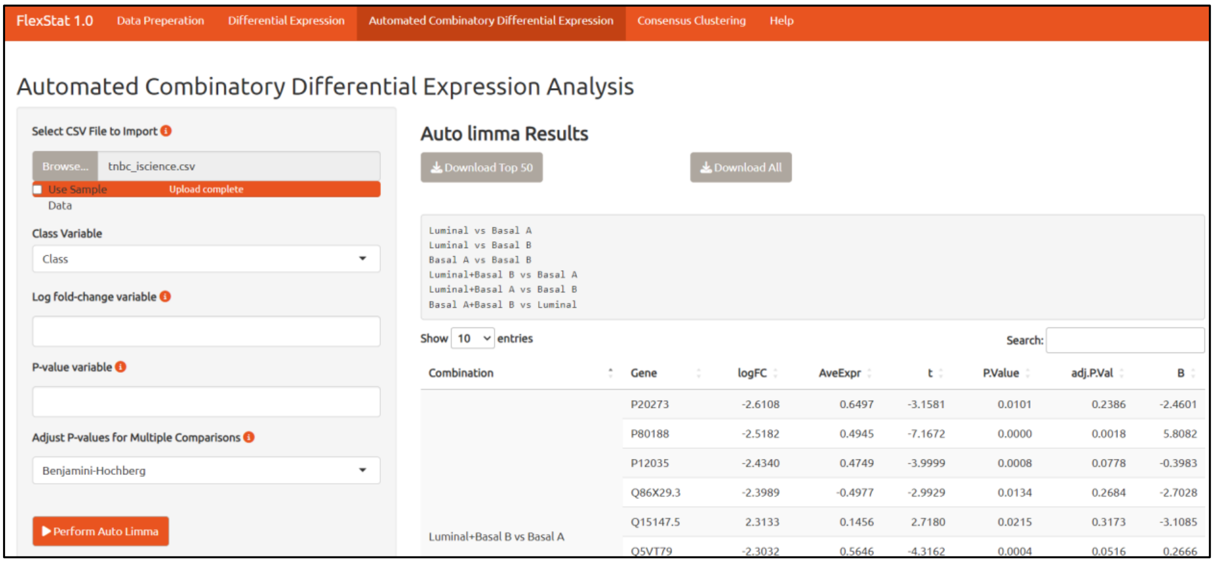

Supplementary Figure 1. Overview of data Analysis of Kosok et al. 2021. (A) Exploratory data analysis of input data (B) Configured statistical parameters and combinatory differential expression analysis of the four three classes (Luminal, Basal A, Basal B)

S2.

A.

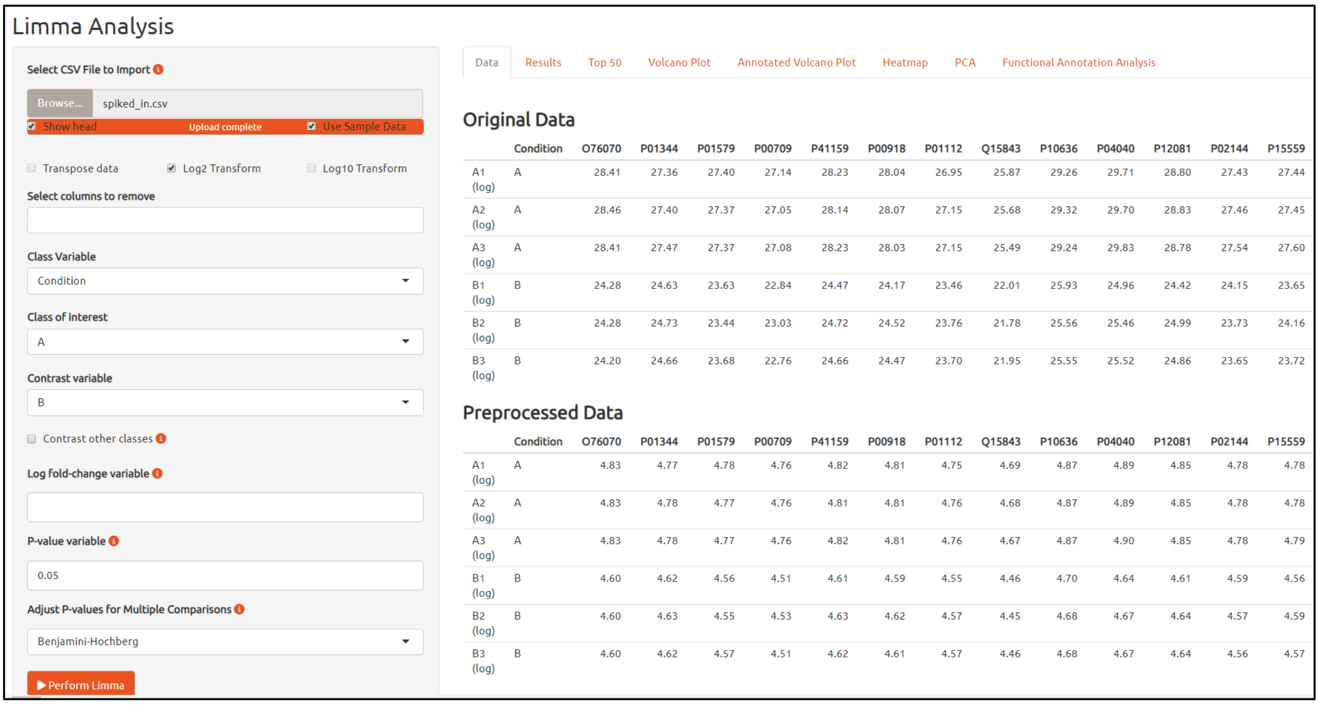

Original Data

	Condition	O76070	P01344	P01579	P00709	P41159	P00918	P01112	Q15843	P10636	P04040	P12081	P02144	P15559
A1 (log)	A	28.41	27.36	27.40	27.14	28.23	28.04	26.95	25.87	29.26	29.71	28.80	27.43	27.44
A2 (log)	A	28.46	27.40	27.37	27.05	28.14	28.07	27.15	25.68	29.32	29.70	28.83	27.46	27.45
A3 (log)	A	28.41	27.47	27.37	27.08	28.23	28.03	27.15	25.49	29.24	29.83	28.78	27.54	27.60
B1 (log)	B	24.28	24.63	23.63	22.84	24.47	24.17	23.46	22.01	25.93	24.96	24.42	24.15	23.65
B2 (log)	B	24.28	24.73	23.44	23.03	24.72	24.52	23.76	21.78	25.56	25.46	24.99	23.73	24.16
B3 (log)	B	24.20	24.66	23.68	22.76	24.66	24.47	23.70	21.95	25.55	25.52	24.86	23.65	23.72

Preprocessed Data

	Condition	O76070	P01344	P01579	P00709	P41159	P00918	P01112	Q15843	P10636	P04040	P12081	P02144	P15559
A1 (log)	A	4.83	4.77	4.78	4.76	4.82	4.81	4.75	4.69	4.87	4.89	4.85	4.78	4.78
A2 (log)	A	4.83	4.78	4.77	4.76	4.81	4.81	4.76	4.68	4.87	4.89	4.85	4.78	4.78
A3 (log)	A	4.83	4.78	4.77	4.76	4.82	4.81	4.76	4.67	4.87	4.90	4.85	4.78	4.79
B1 (log)	B	4.60	4.62	4.56	4.51	4.61	4.59	4.55	4.46	4.70	4.64	4.61	4.59	4.56
B2 (log)	B	4.60	4.63	4.55	4.53	4.63	4.62	4.57	4.45	4.68	4.67	4.64	4.57	4.59
B3 (log)	B	4.60	4.62	4.57	4.51	4.62	4.61	4.57	4.46	4.68	4.67	4.64	4.56	4.57

B.

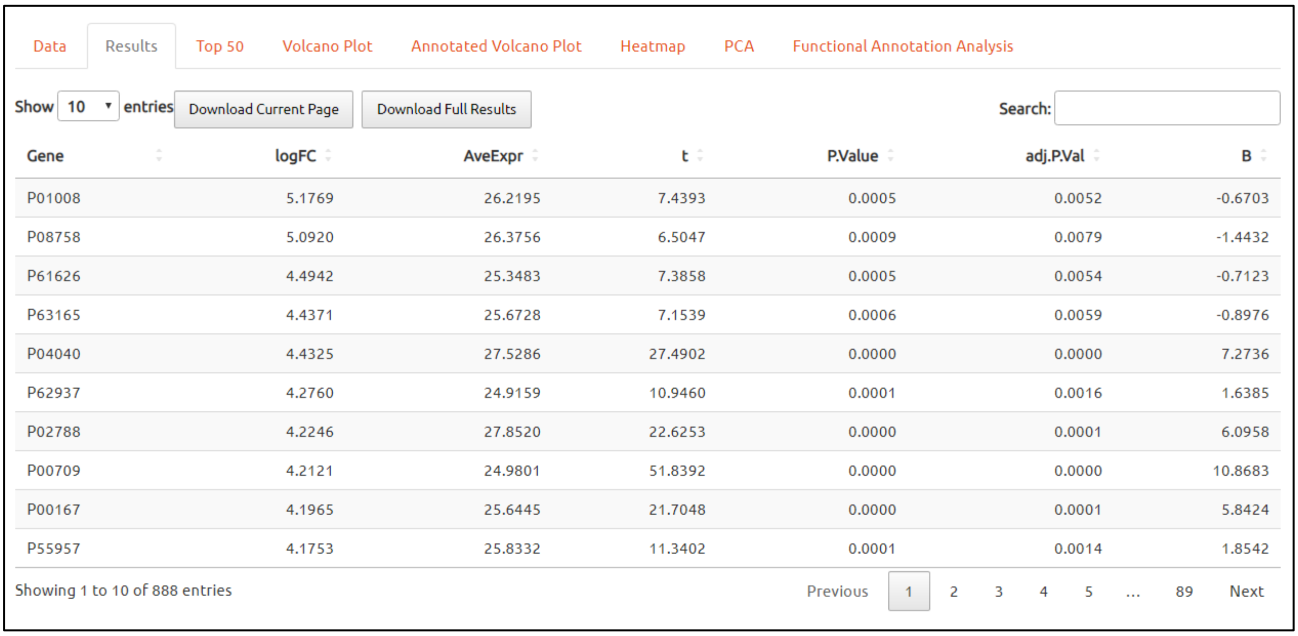

Gene	logFC	AveExpr	t	P.Value	adj.P.Val	B
P01008	5.1769	26.2195	7.4393	0.0005	0.0052	-0.6703
P08758	5.0920	26.3756	6.5047	0.0009	0.0079	-1.4432
P61626	4.4942	25.3483	7.3858	0.0005	0.0054	-0.7123
P63165	4.4371	25.6728	7.1539	0.0006	0.0059	-0.8976
P04040	4.4325	27.5286	27.4902	0.0000	0.0000	7.2736
P62937	4.2760	24.9159	10.9460	0.0001	0.0016	1.6385
P02788	4.2246	27.8520	22.6253	0.0000	0.0001	6.0958
P00709	4.2121	24.9801	51.8392	0.0000	0.0000	10.8683
P00167	4.1965	25.6445	21.7048	0.0000	0.0001	5.8424
P55957	4.1753	25.8332	11.3402	0.0001	0.0014	1.8542

C.

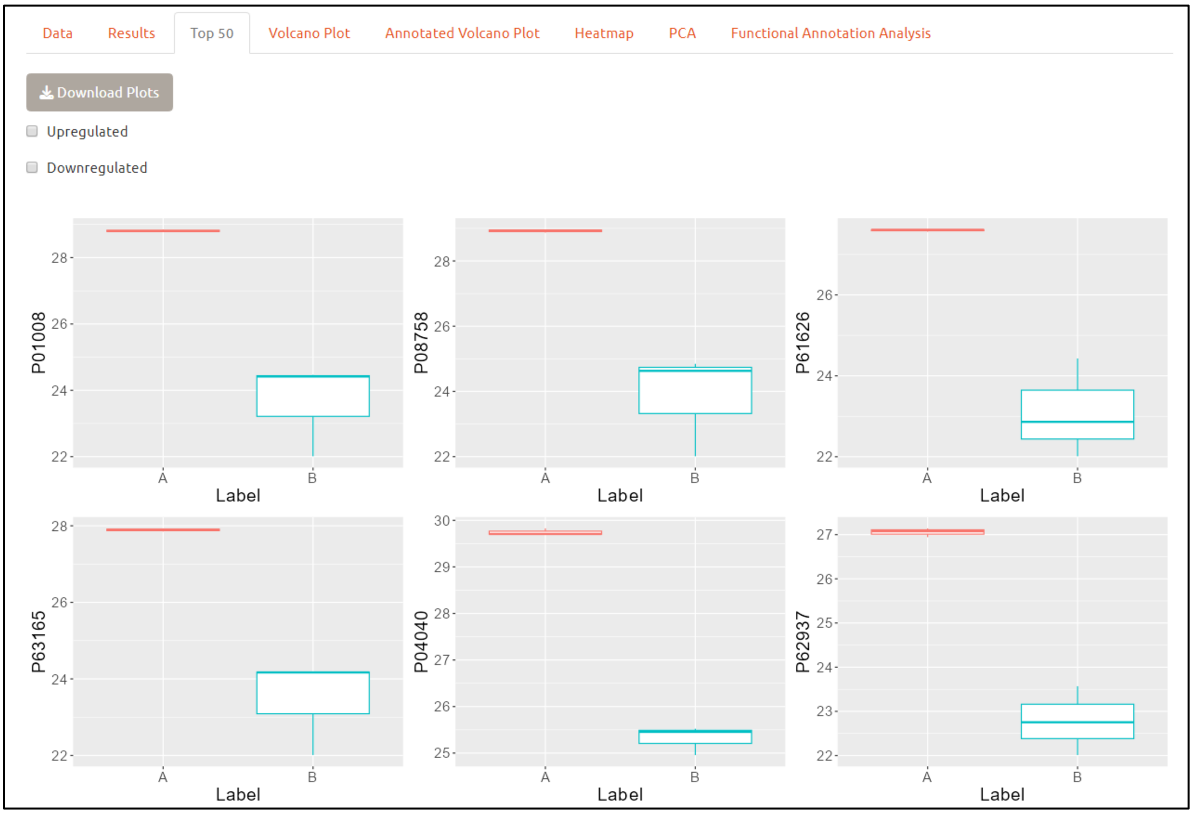
Data
Results
Top 50
Volcano Plot
Annotated Volcano Plot
Heatmap
PCA
Functional Annotation Analysis
Download Plots
Upregulated
Downregulated
P01008
P08758
P61626
P63165
P04040
P62937
Label
A
B

D.

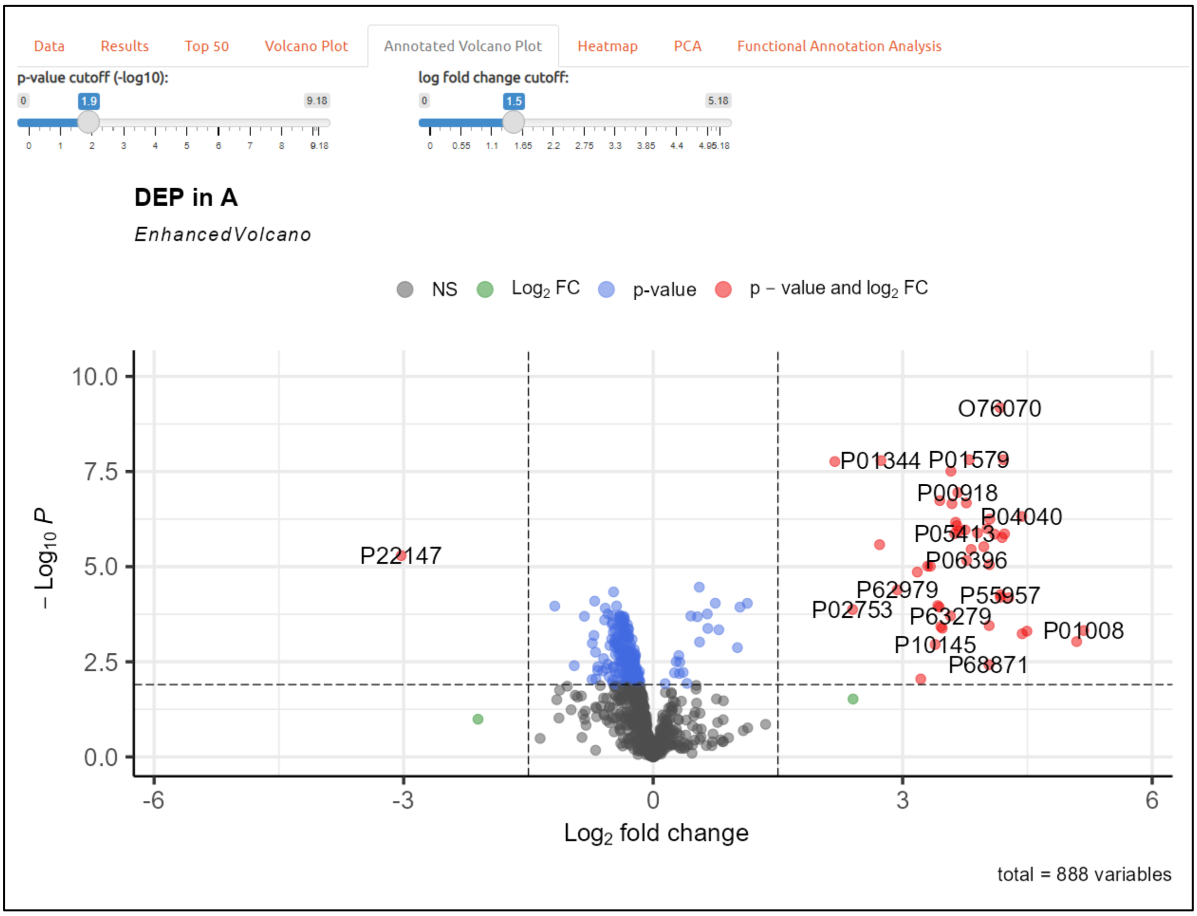
Data
Results
Top 50
Volcano Plot
Annotated Volcano Plot
Heatmap
PCA
Functional Annotation Analysis
p-value cutoff (-log10):
log fold change cutoff:
DEP in A
EnhancedVolcano
NS
Log2 FC
p-value
p – value and log2 FC
– Log10 P
Log2 fold change
O76070
P01344 P01579
P00918
P04040
P05413
P06396
P22147
P62979
P55957
P02753
P63279
P01008
P10145
P68871
total = 888 variables

E.

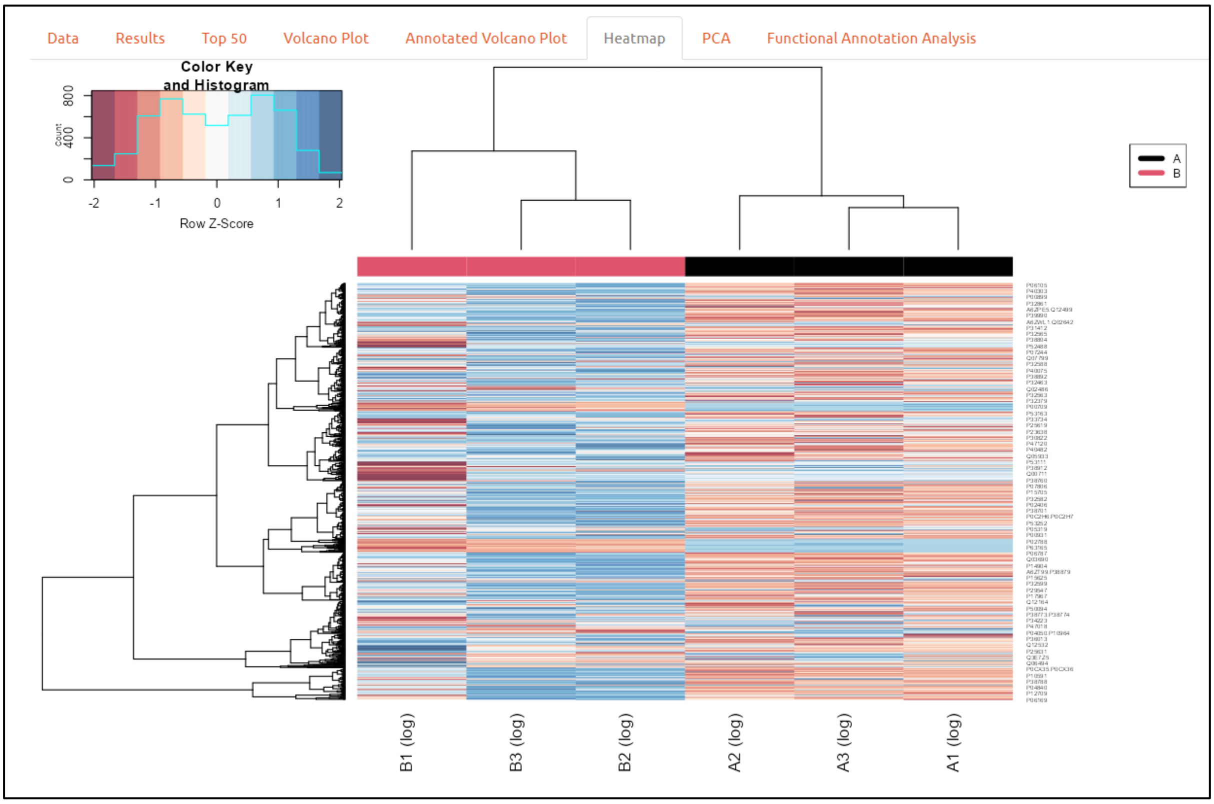

Supplementary Figure 2. Overview of Spiked-in Data Analysis of Ramus et al. 2016. (A) Exploratory data analysis; log-transformed input data (B) Configure statistical parameters and run differential expression analysis. (C) Boxplot visualization of the dysregulated proteins across conditions A and B. (D) Volcano plot depicting the upregulated set of proteins. Green-colored proteins are verified to be the spiked-in proteins E. Heatmap showing clear distinction among conditions A and B.

S3.

No.	Study	Acquisition	Labelling	No. of Proteins	No. of samples	No. of classes	Upload (in sec)	Differential expression (in sec)	Combinatory DEP (in sec)	Consensus clustering (in sec)
1	UPS1 spiked-in yeast data (Ramus et al., 2016)	DDA	LFQ	888	6	2	3.63	10.02	4.00	2.80
2	Ubiquitin interactor affinity enriched data (Zhang et al., 2018)	DDA	LFQ	1899	12	4	7.49	14.66	11.17	4.38
3	TNBC cellline data (Kosok et al. 2021)	DDA	TMT	11709	18	3	60.18	73.75	36.50	5.46
4	Clear cell renal carcinoma data (Guo et al., 2015)	SWATH		2375	24	2	12.63	48.80	3.67	5.95
5	Ovarian cancer data (Zhang et al., 2016)	DIA	TMT	8074	103	2	20.36	271.93	24.35	9.67

Supplementary Figure 3. Overview of mean execution elapsed times of limma-based differential expression analysis and combinatory differential expression analysis, and consensus clustering on test datasets on 100 iterations of execution.

S4.

A.

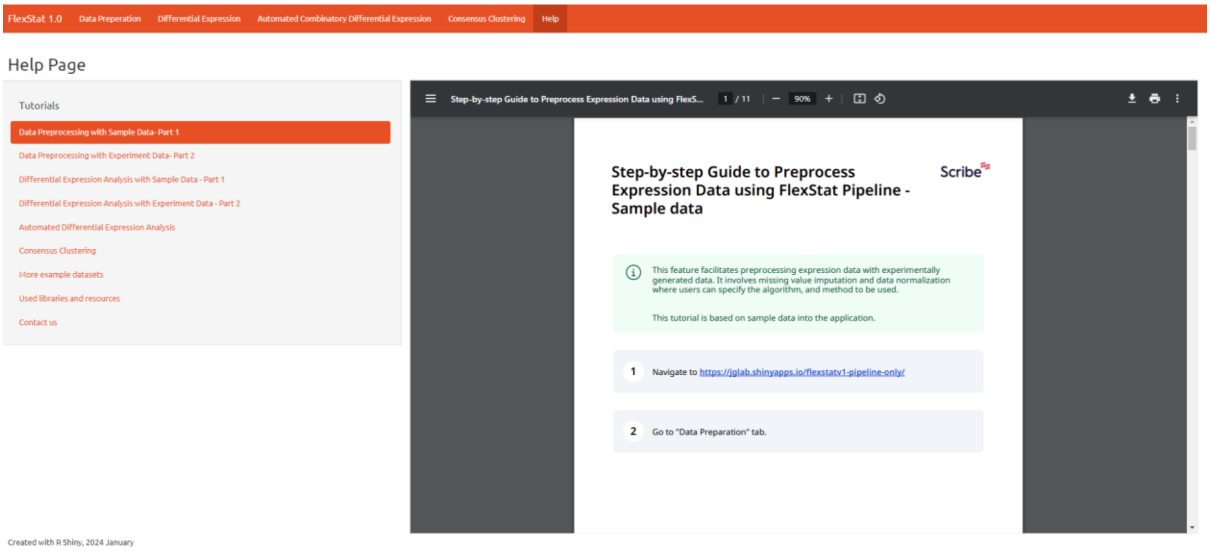
FlexStat 1.0
Data Preperation
Differential Expression
Automated Combinatory Differential Expression
Consensus Clustering
Help
Help Page
Tutorials
Data Preprocessing with Sample Data- Part 1
Data Preprocessing with Experiment Data- Part 2
Differential Expression Analysis with Sample Data - Part 1
Differential Expression Analysis with Experiment Data - Part 2
Automated Differential Expression Analysis
Consensus Clustering
More example datasets
Used libraries and resources
Contact us
Created with R Shiny, 2024 January
Step-by-step Guide to Preprocess Expression Data using FlexS...
90%
Step-by-step Guide to Preprocess Expression Data using FlexStat Pipeline - Sample data
Scribe
This feature facilitates preprocessing expression data with experimentally generated data. It involves missing value imputation and data normalization where users can specify the algorithm, and method to be used.
This tutorial is based on sample data into the application.
1 Navigate to https://jglab.shinyapps.io/flexstatv1-pipeline-only/
2 Go to "Data Preparation" tab.

B.

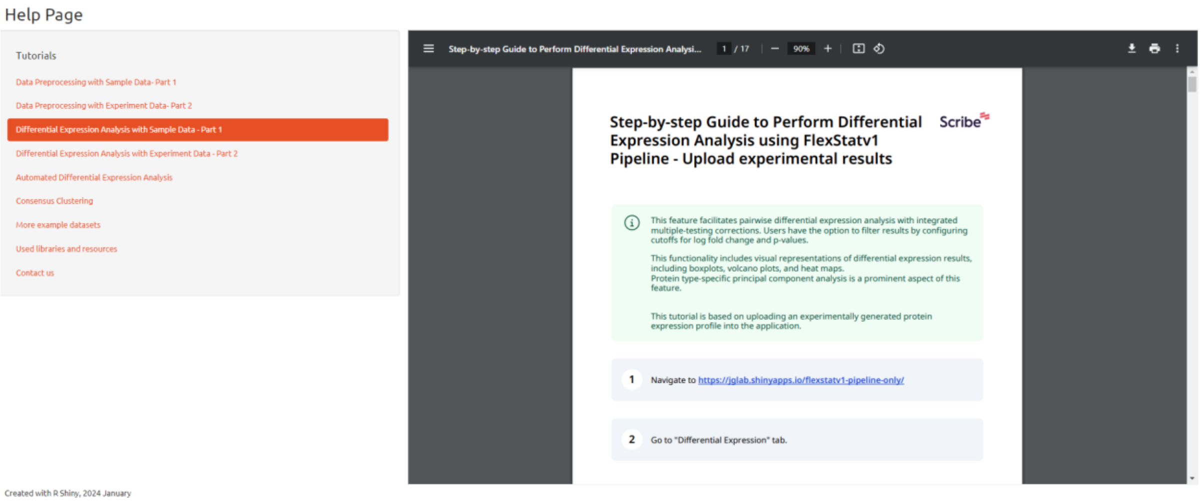
Help Page
Tutorials
Data Preprocessing with Sample Data- Part 1
Data Preprocessing with Experiment Data- Part 2
Differential Expression Analysis with Sample Data - Part 1
Differential Expression Analysis with Experiment Data - Part 2
Automated Differential Expression Analysis
Consensus Clustering
More example datasets
Used libraries and resources
Contact us
Created with R Shiny, 2024 January
Step-by-step Guide to Perform Differential Expression Analysi...
90%
Step-by-step Guide to Perform Differential Expression Analysis using FlexStatv1 Pipeline - Upload experimental results
Scribe
This feature facilitates pairwise differential expression analysis with integrated multiple-testing corrections. Users have the option to filter results by configuring cutoffs for log fold change and p-values.
This functionality includes visual representations of differential expression results, including boxplots, volcano plots, and heat maps.
Protein type-specific principal component analysis is a prominent aspect of this feature.
This tutorial is based on uploading an experimentally generated protein expression profile into the application.
1 Navigate to https://jglab.shinyapps.io/flexstatv1-pipeline-only/
2 Go to "Differential Expression" tab.

C.

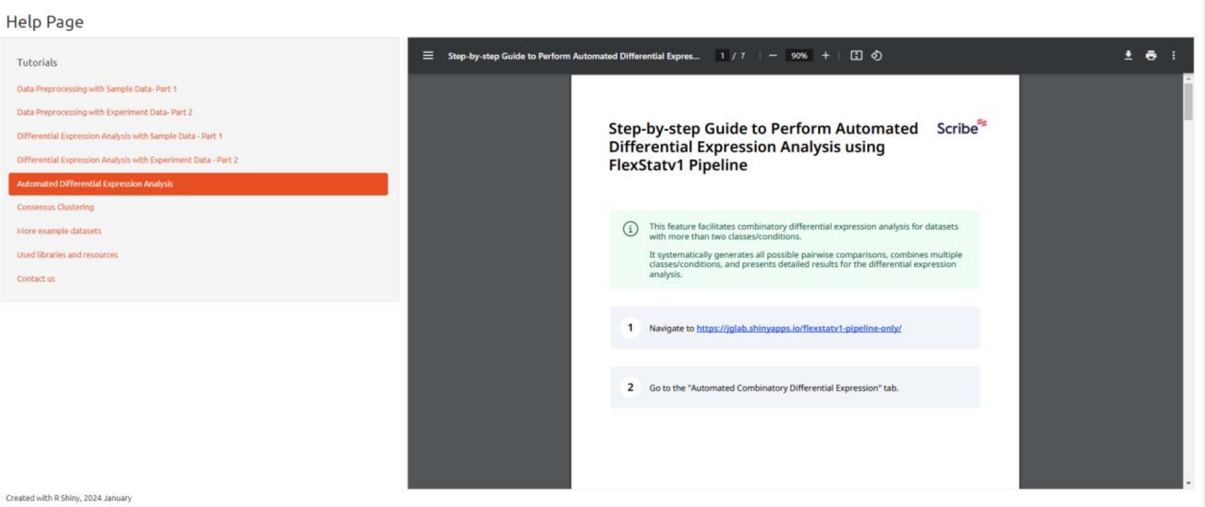
Help Page
Tutorials
Data Preprocessing with Sample Data- Part 1
Data Preprocessing with Experiment Data- Part 2
Differential Expression Analysis with Sample Data - Part 1
Differential Expression Analysis with Experiment Data - Part 2
Automated Differential Expression Analysis
Consensus Clustering
More example datasets
Used libraries and resources
Contact us
Created with R Shiny, 2024 January
Step-by-step Guide to Perform Automated Differential Expres...
90%
Step-by-step Guide to Perform Automated Differential Expression Analysis using FlexStatv1 Pipeline
Scribe
This feature facilitates combinatory differential expression analysis for datasets with more than two classes/conditions.
It systematically generates all possible pairwise comparisons, combines multiple classes/conditions, and presents detailed results for the differential expression analysis.
1 Navigate to https://jglab.shinyapps.io/flexstatv1-pipeline-only/
2 Go to the "Automated Combinatory Differential Expression" tab.

D.

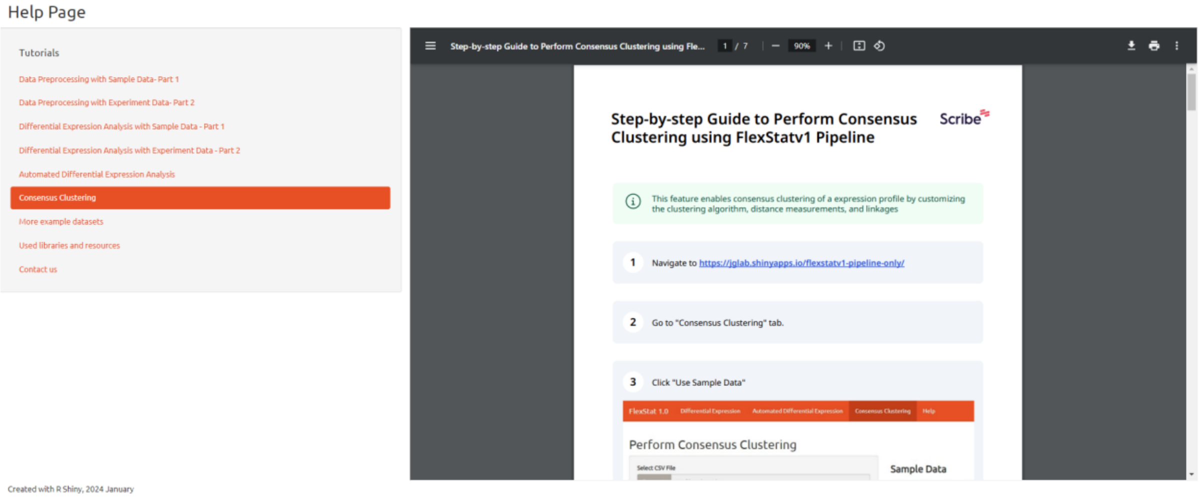

Supplementary Figure 4: FlexStat documentation and tutorials of the functionalities of A. Data Preprocessing B. Differential expression analysis, C. Automated Combinatory Differential Expression Analysis, D. Consensus Clustering.
