# Supplementary material for: FlexStat: combinatory differentially expressed protein extraction
Source: Bioinform Adv. 2024 Apr 11;4(1):vbae056. doi: 10.1093/bioadv/vbae056 (PMC11055397; doi:10.1093/bioadv/vbae056)
Supplement: vbae056_Supplementary_Data [file vbae056_supplementary_data.zip › Supplementary_User_Guide.pdf]

FlexStat User Guide

version 1.0

Senuri De Silva

Jan 19, 2024

Table of Contents

Preprocess Expression Data - Sample data 3

Preprocess Expression Data - Upload experimental results 12

Perform Differential Expression Analysis - Using Sample Data 20

Differential Expression Analysis – Upload experimental results 30

Perform Automated Differential Expression Analysis 42

Perform Consensus Clustering 49

Step-by-step Guide to Preprocess Expression Data using FlexStat Pipeline - Sample data

Scribe

This feature facilitates preprocessing expression data with experimentally generated data. It involves missing value imputation and data normalization where users can specify the algorithm, and method to be used.

This tutorial is based on sample data into the application.

1 Navigate to https://jglab.shinyapps.io/flexstatv1-pipeline-only/

2 Go to "Data Preparation" tab.

3 Click "Use Sample Data"

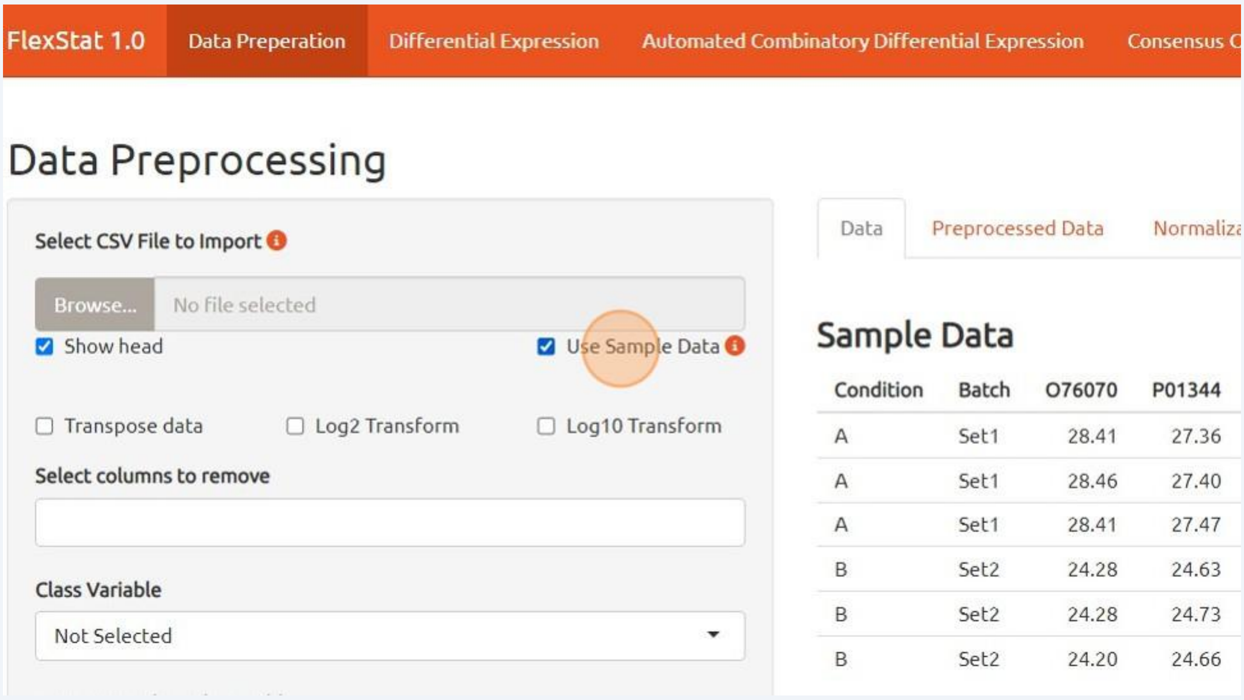

4 Select 'Class' variable to generate quality control plots.

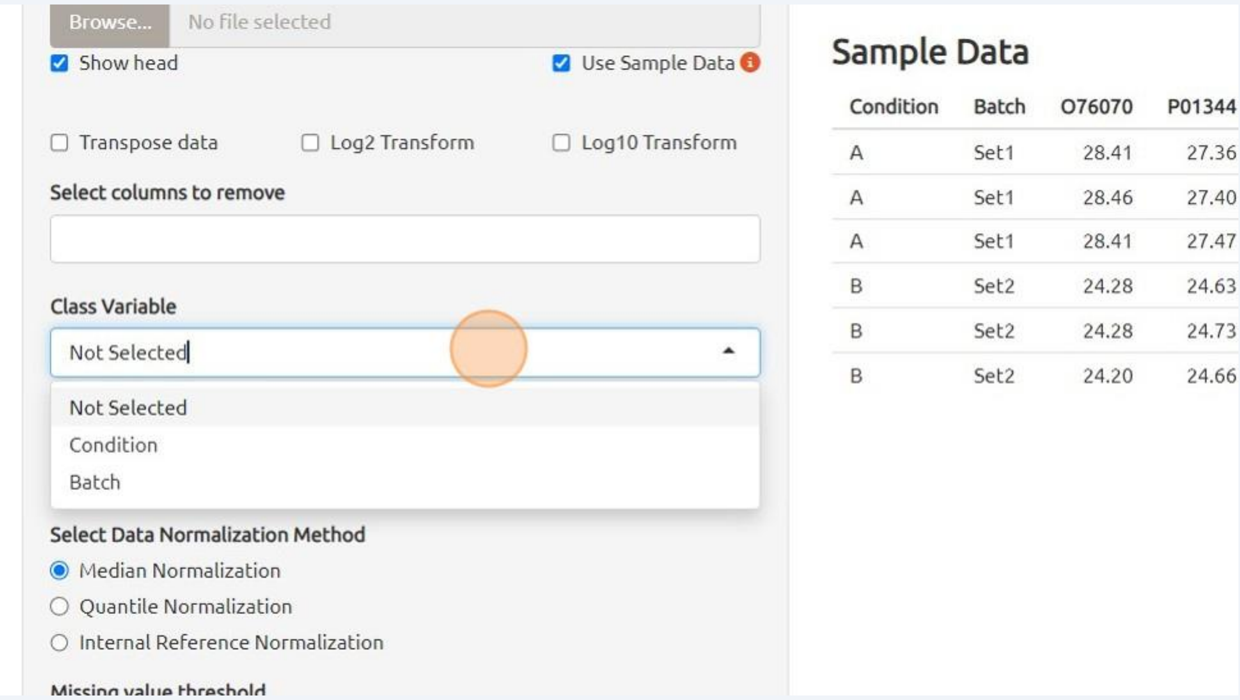

5 Select "Experimental batch variable" to be used in normalization.

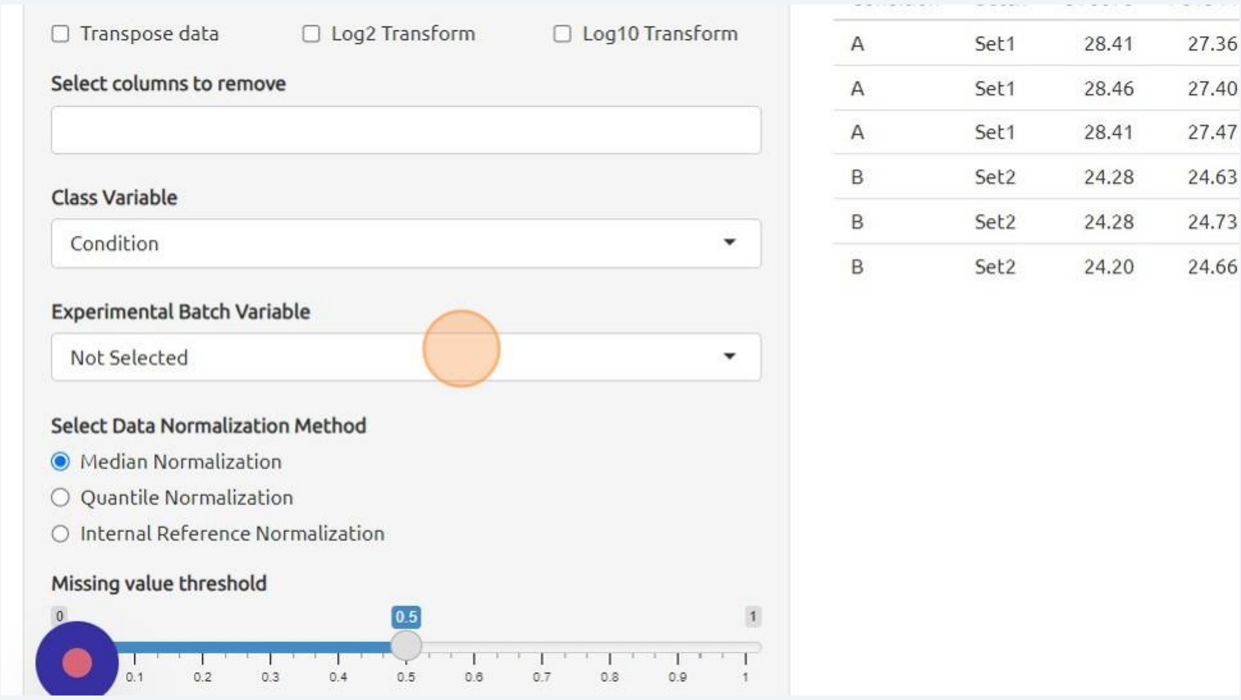

6 Select missing value threshold. Default is 0.5 which means proteins with 50% or less missing values will be considered for imputation.

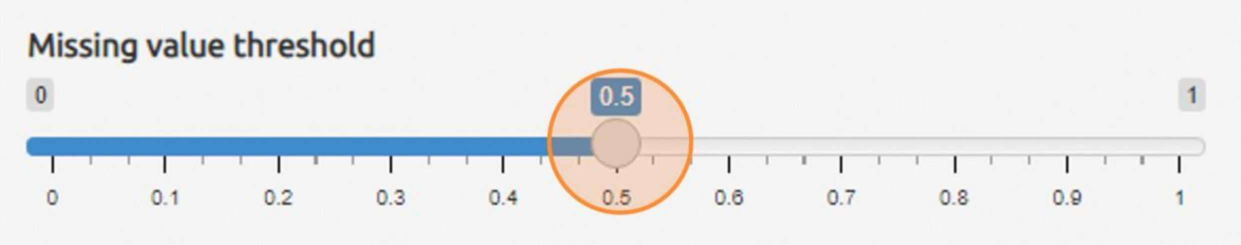

7 Select Data Normalization method

1. Median normalization
2. Quantile normalization
3. Internal Reference Normalization: Recommended to use for label-based data
4. if selected, select the corresponding internal reference to be used
5. Variance Stabilization Normalization: Recommended to use for label-free data

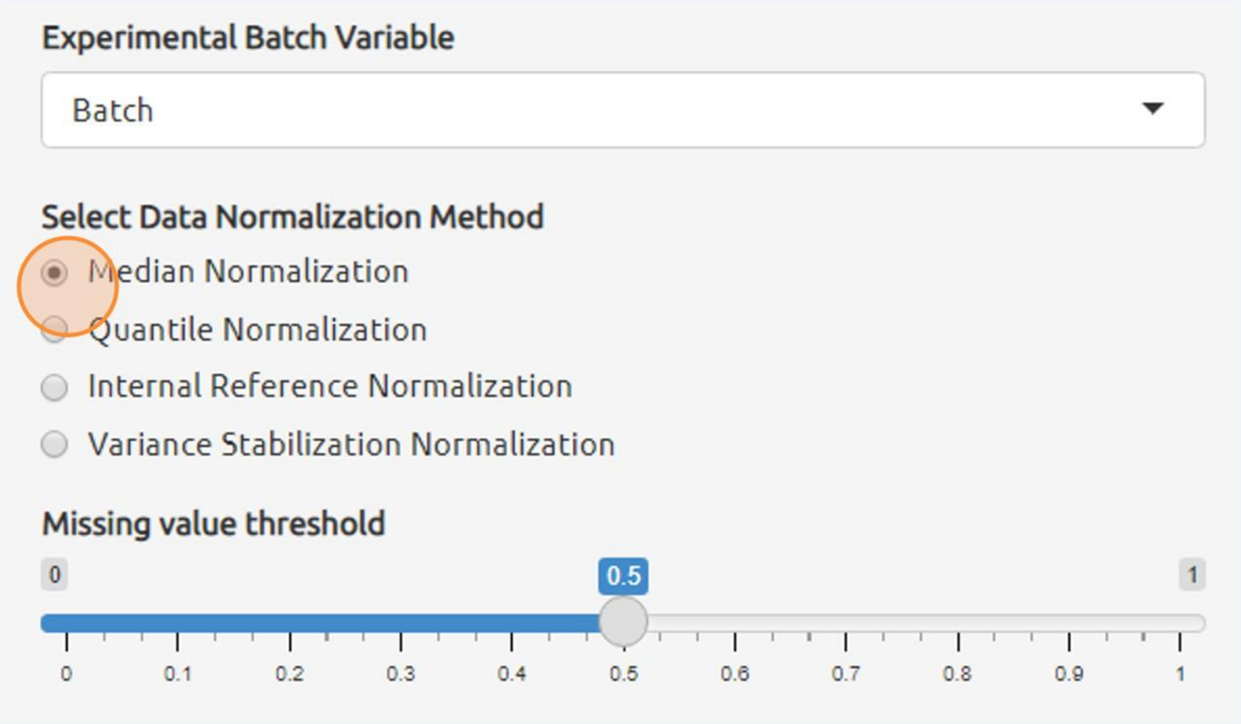

8 **Internal Reference Normalization:** if selected, then select the corresponding internal reference to be used.

Select the corresponding internal reference to be used. Here we select "A1 (log)" and "B1 (log)".

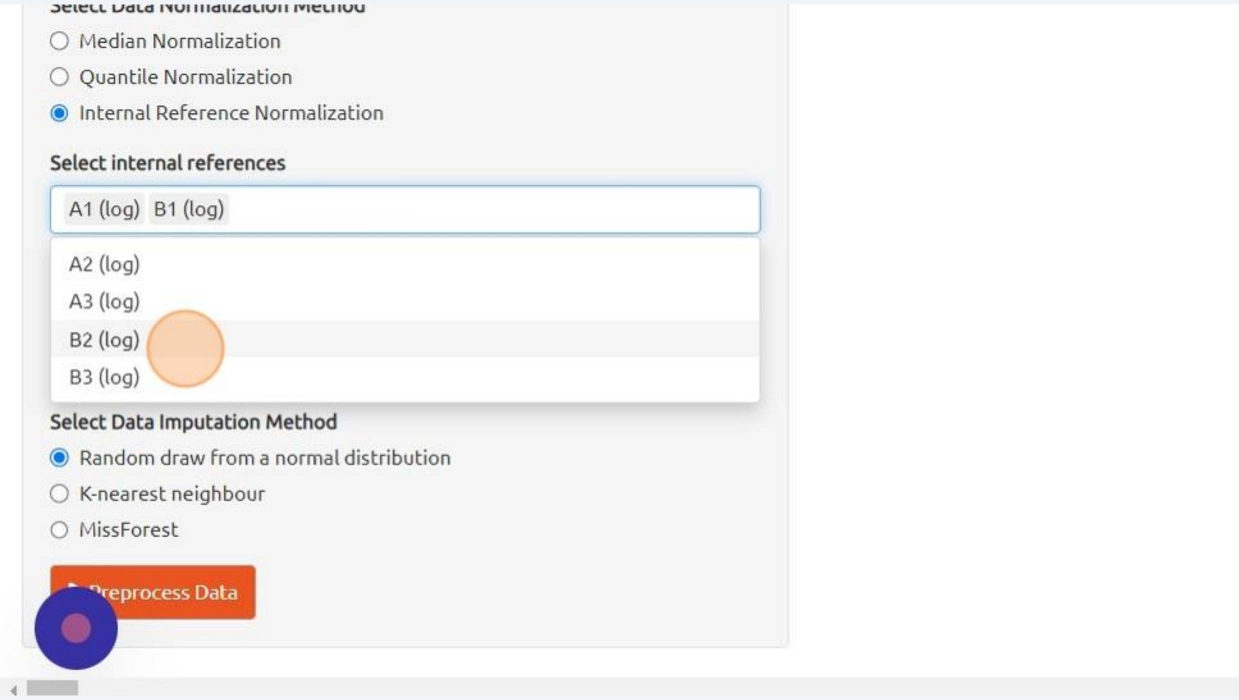

9 Click "Preprocess Data"

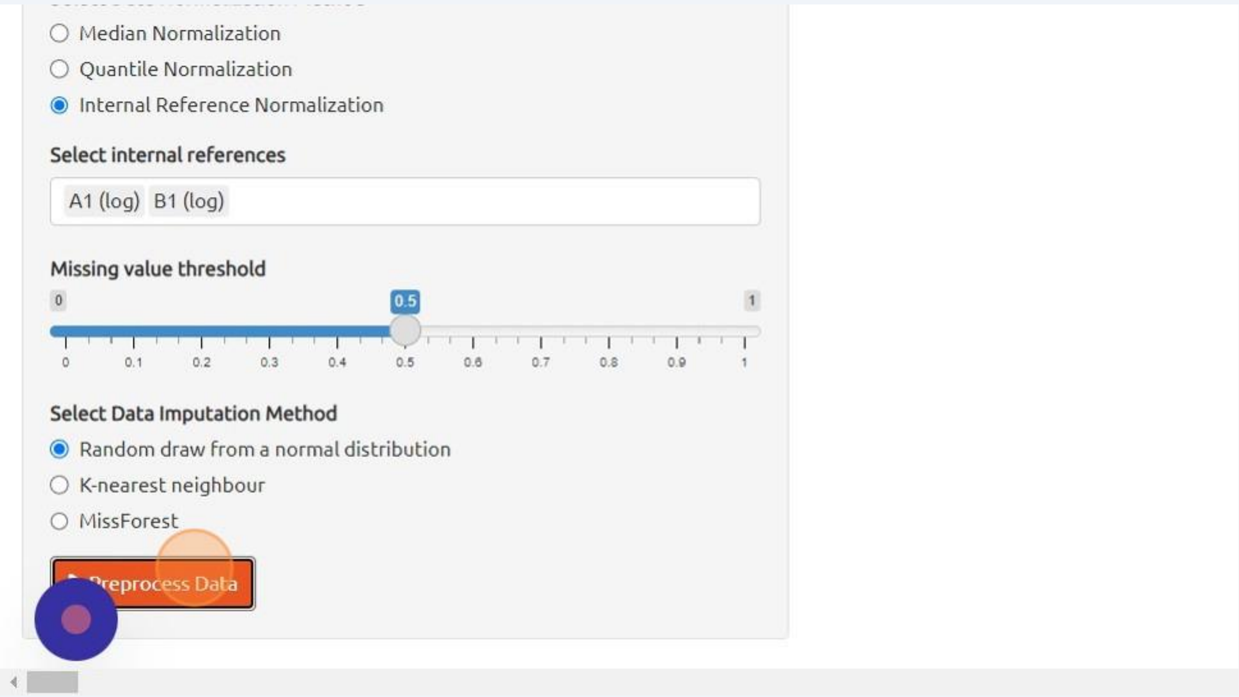

10 Click "Preprocessed Data" to check the preprocessing results.

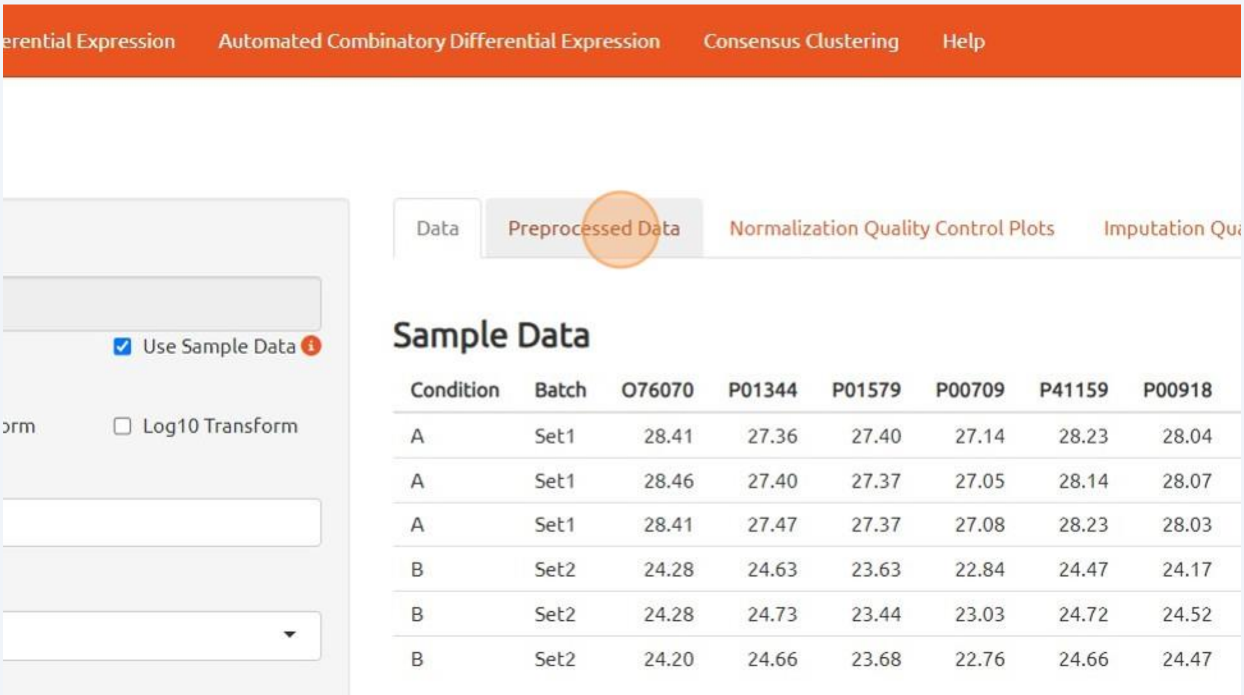

11 Click "Download Normalized Data" to download the normalized data.

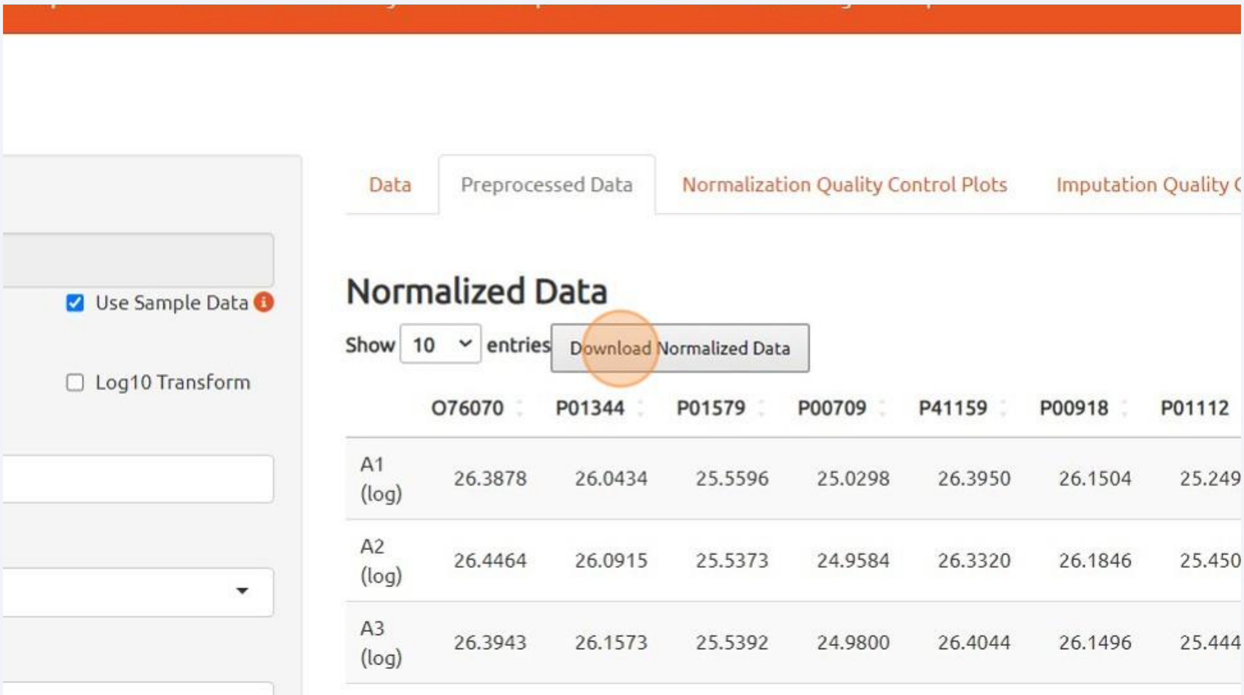

12 Click "Download Imputed Data" to download the imputed data.

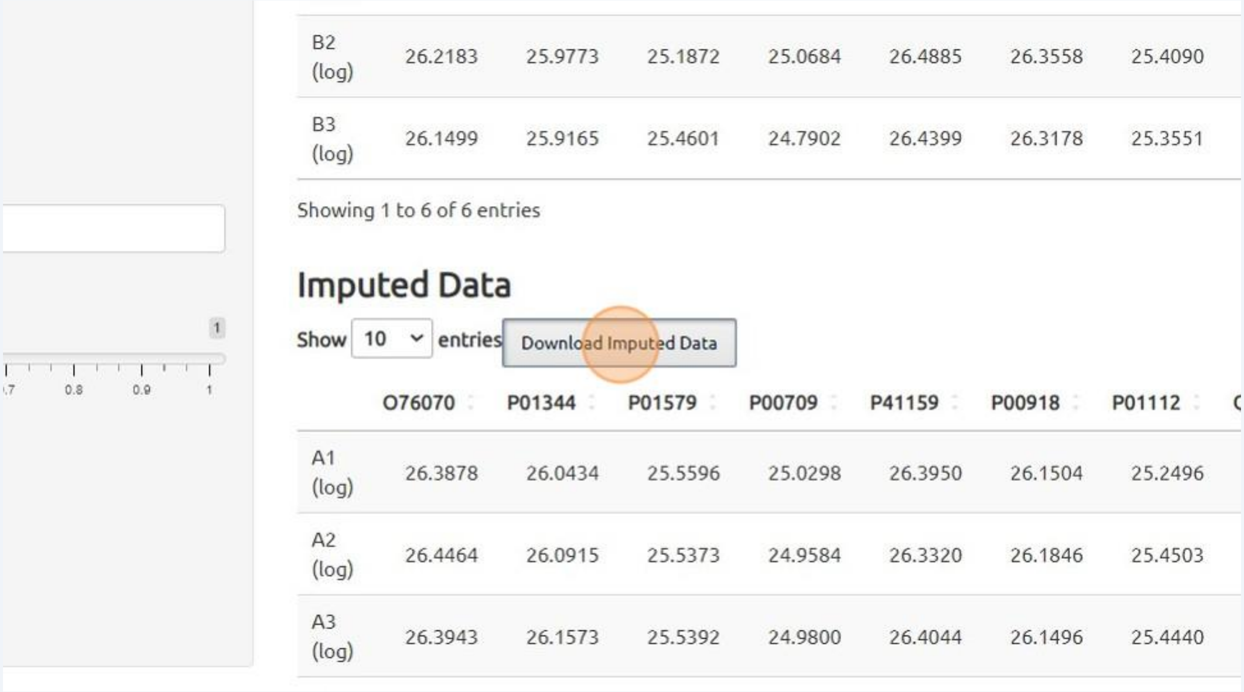

13 Click "Normalization Quality Control Plots" to view the outputs of normalization steps.

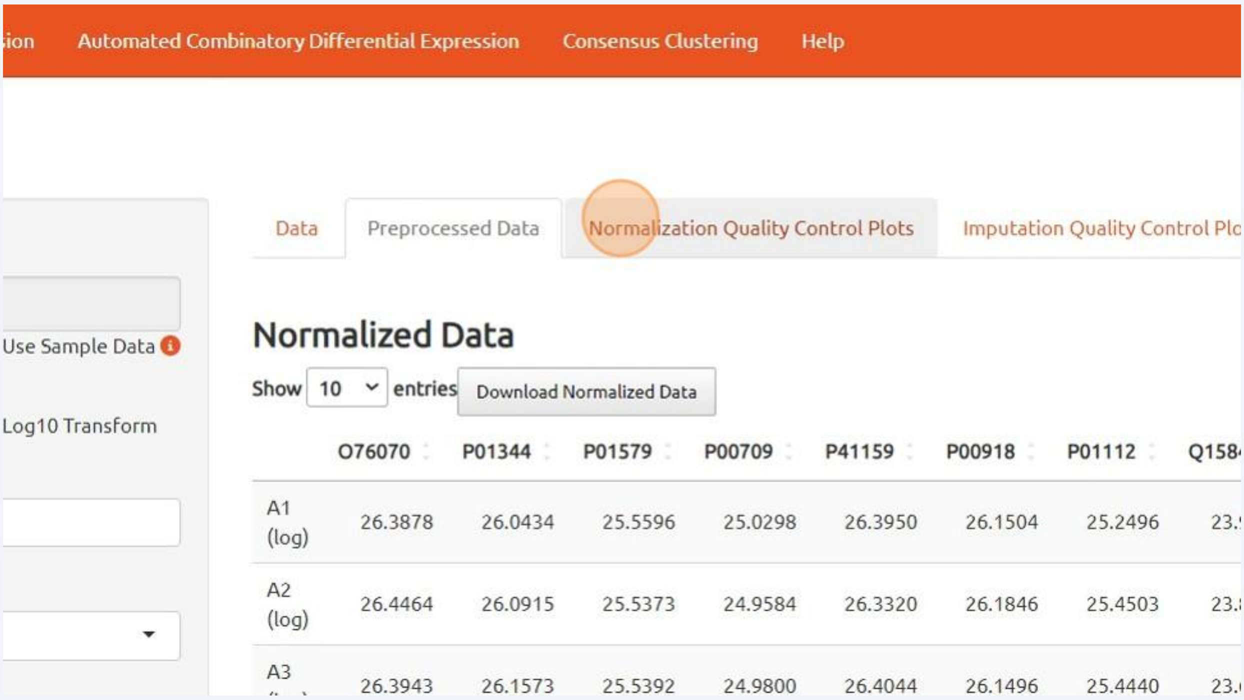

14 Click "Normalization Quality Control Plots" to view the outputs of normalization steps.

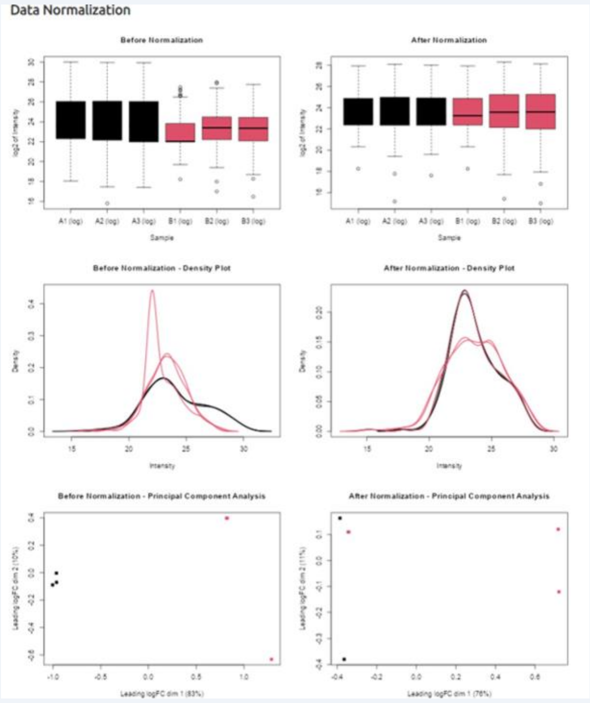

15 Click "Imputation Quality Control Plots"

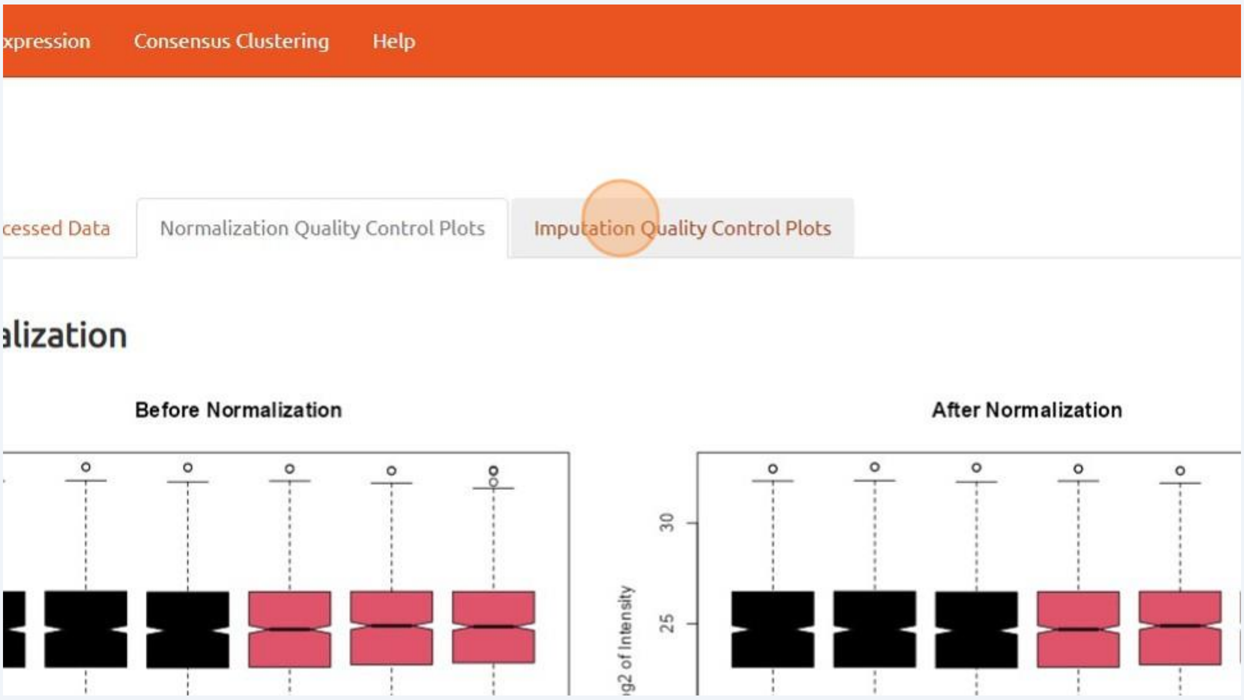

16 Click "Imputation Quality Control Plots"

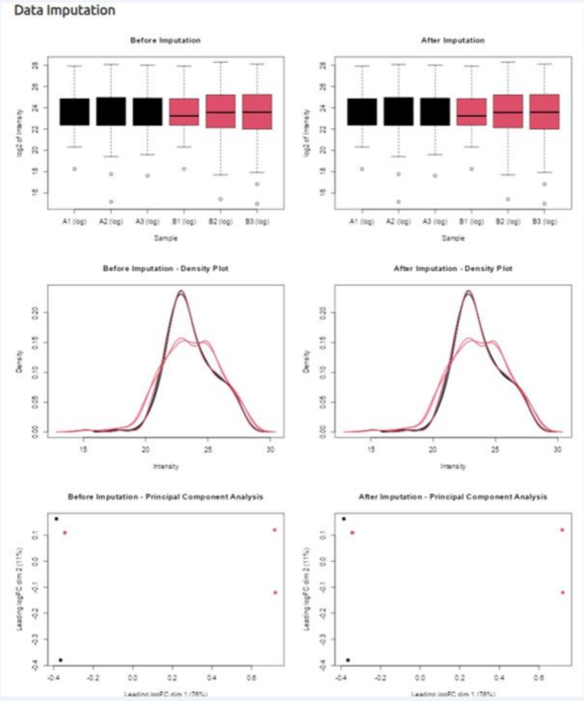

Step-by-step Guide to Preprocess Expression Data using FlexStat Pipeline - Upload experimental results

This feature facilitates preprocessing expression data with experimentally generated data. It involves missing value imputation and data normalization where users can specify the algorithm, and method to be used.

This tutorial is based on uploading an experimentally generated protein expression profile into the application.

1 Navigate to https://jglab.shinyapps.io/flexstatv1-pipeline-only/

2 Go to "Data Preparation" tab.

3 Upload the expression file having rows as the samples and protein names in the columns and having a column for experimental condition/class as shown in the right-side panel.

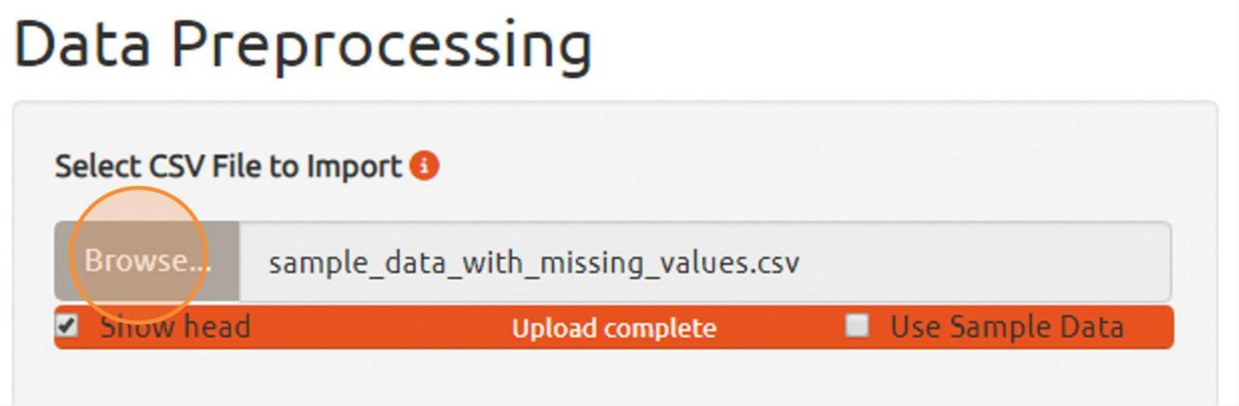

4 The uploaded data should be shown in the right-side panel.

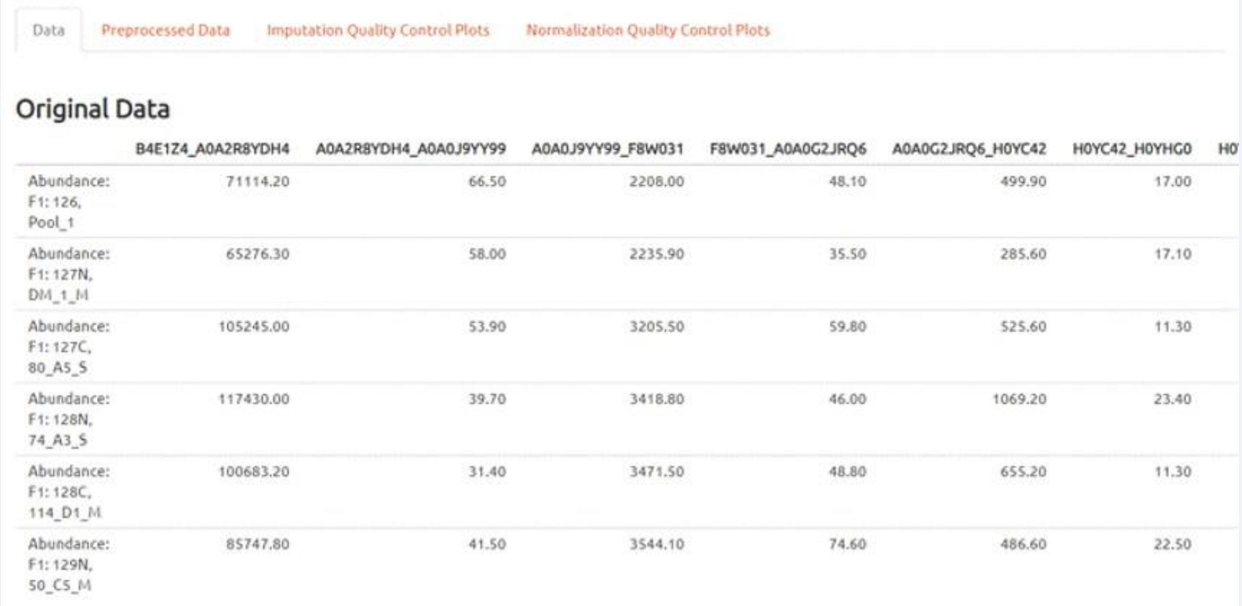

	B4E1Z4_A0A2R8YDH4	A0A2R8YDH4_A0A0J9YY99	A0A0J9YY99_F8W031	F8W031_A0A0G2JRQ6	A0A0G2JRQ6_H0YC42	H0YC42_H0YHG0
Abundance: F1: 126, Pool_1	71114.20	66.50	2208.00	48.10	499.90	17.00
Abundance: F1: 127N, DM_1_M	65276.30	58.00	2235.90	35.50	285.60	17.10
Abundance: F1: 127C, 80_A5_S	105245.00	53.90	3205.50	59.80	525.60	11.30
Abundance: F1: 128N, 74_A3_S	117430.00	39.70	3418.80	46.00	1069.20	23.40
Abundance: F1: 128C, 114_D1_M	100683.20	31.40	3471.50	48.80	655.20	11.30
Abundance: F1: 129N, 50_C5_M	85747.80	41.50	3544.10	74.60	486.60	22.50

5 [Optional] Transform data into log scale if needed.

NOTE: This example data is raw abundance values, therefore here we are applying log10 scale to transform.

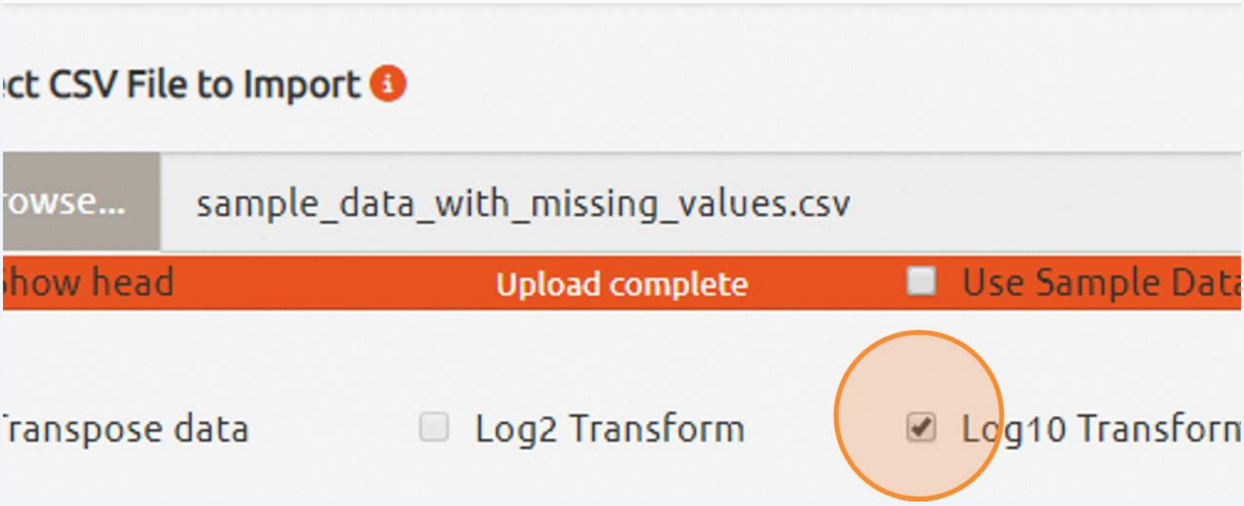

6 Transformed data is shown in the bottom right panel.

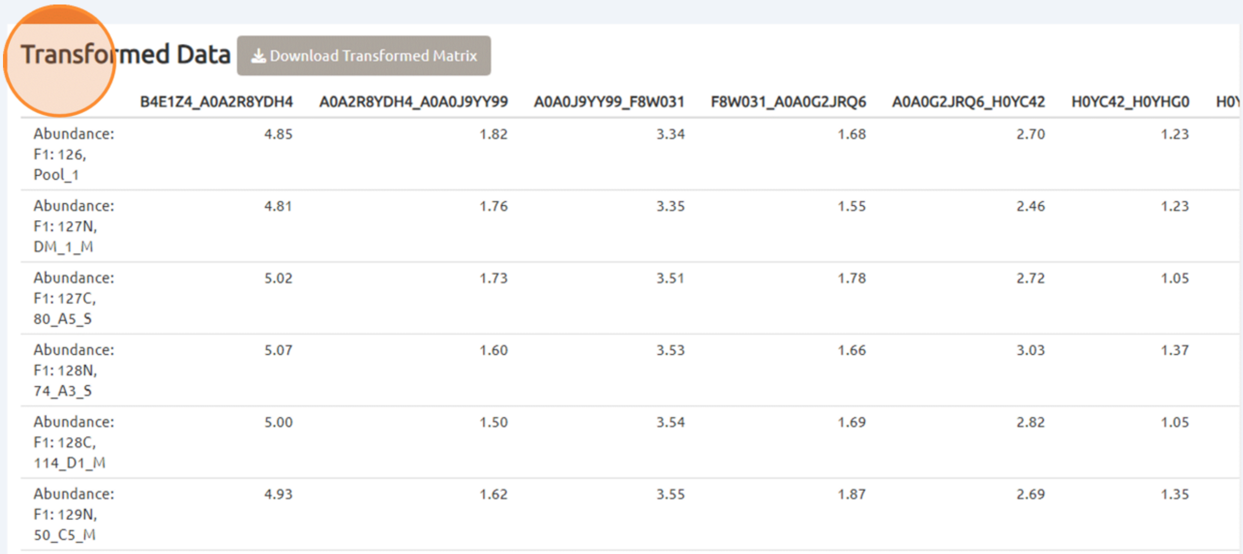

Transformed Data | Download Transformed Matrix

	B4E1Z4_A0A2R8YDH4	A0A2R8YDH4_A0A0J9YY99	A0A0J9YY99_F8W031	F8W031_A0A0G2JRQ6	A0A0G2JRQ6_H0YC42	H0YC42_H0YHG0	H0Y
Abundance: F1: 126, Pool_1	4.85	1.82	3.34	1.68	2.70	1.23	
Abundance: F1: 127N, DM_1_M	4.81	1.76	3.35	1.55	2.46	1.23	
Abundance: F1: 127C, 80_A5_S	5.02	1.73	3.51	1.78	2.72	1.05	
Abundance: F1: 128N, 74_A3_S	5.07	1.60	3.53	1.66	3.03	1.37	
Abundance: F1: 128C, 114_D1_M	5.00	1.50	3.54	1.69	2.82	1.05	
Abundance: F1: 129N, 50_C5_M	4.93	1.62	3.55	1.87	2.69	1.35	

7 Transformed data can be downloaded for future reference.

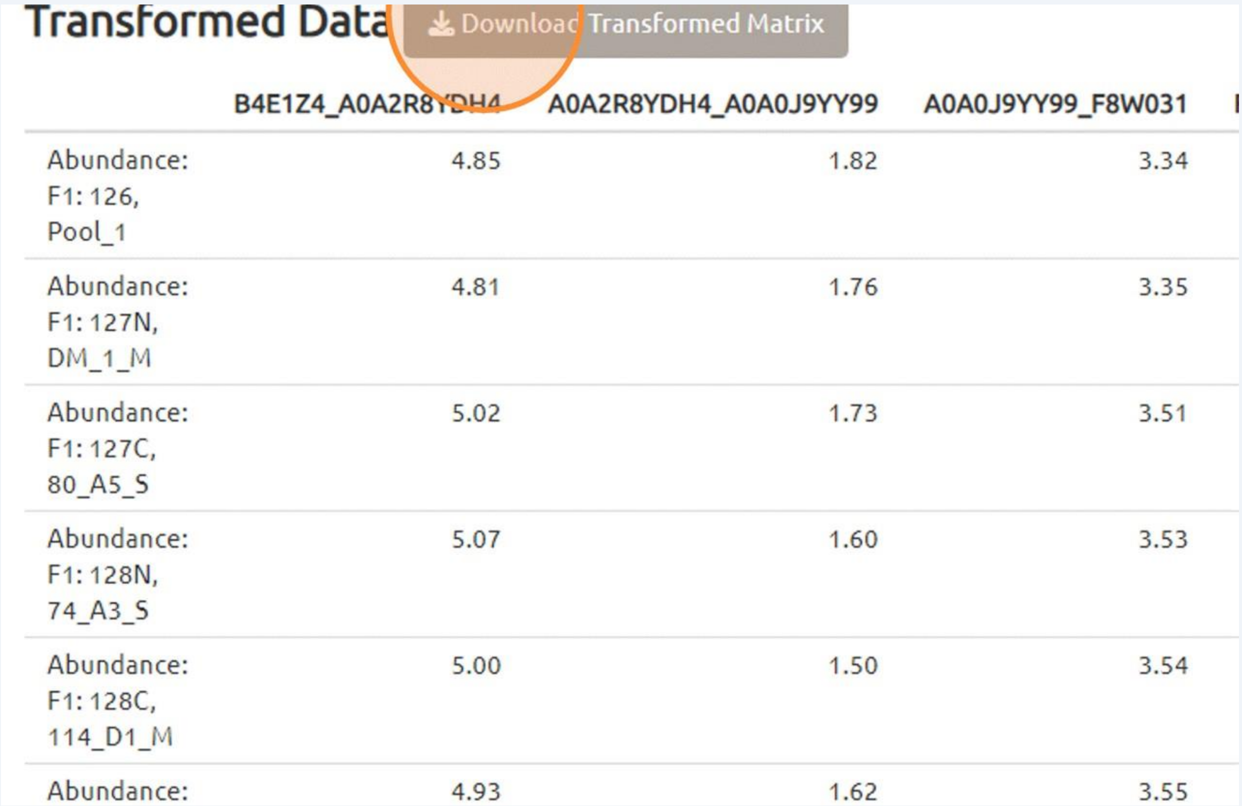

Transformed Data | Download Transformed Matrix

	B4E1Z4_A0A2R8YDH4	A0A2R8YDH4_A0A0J9YY99	A0A0J9YY99_F8W031
Abundance: F1: 126, Pool_1	4.85	1.82	3.34
Abundance: F1: 127N, DM_1_M	4.81	1.76	3.35
Abundance: F1: 127C, 80_A5_S	5.02	1.73	3.51
Abundance: F1: 128N, 74_A3_S	5.07	1.60	3.53
Abundance: F1: 128C, 114_D1_M	5.00	1.50	3.54
Abundance:	4.93	1.62	3.55

8 [Optional] Select columns to be removed.

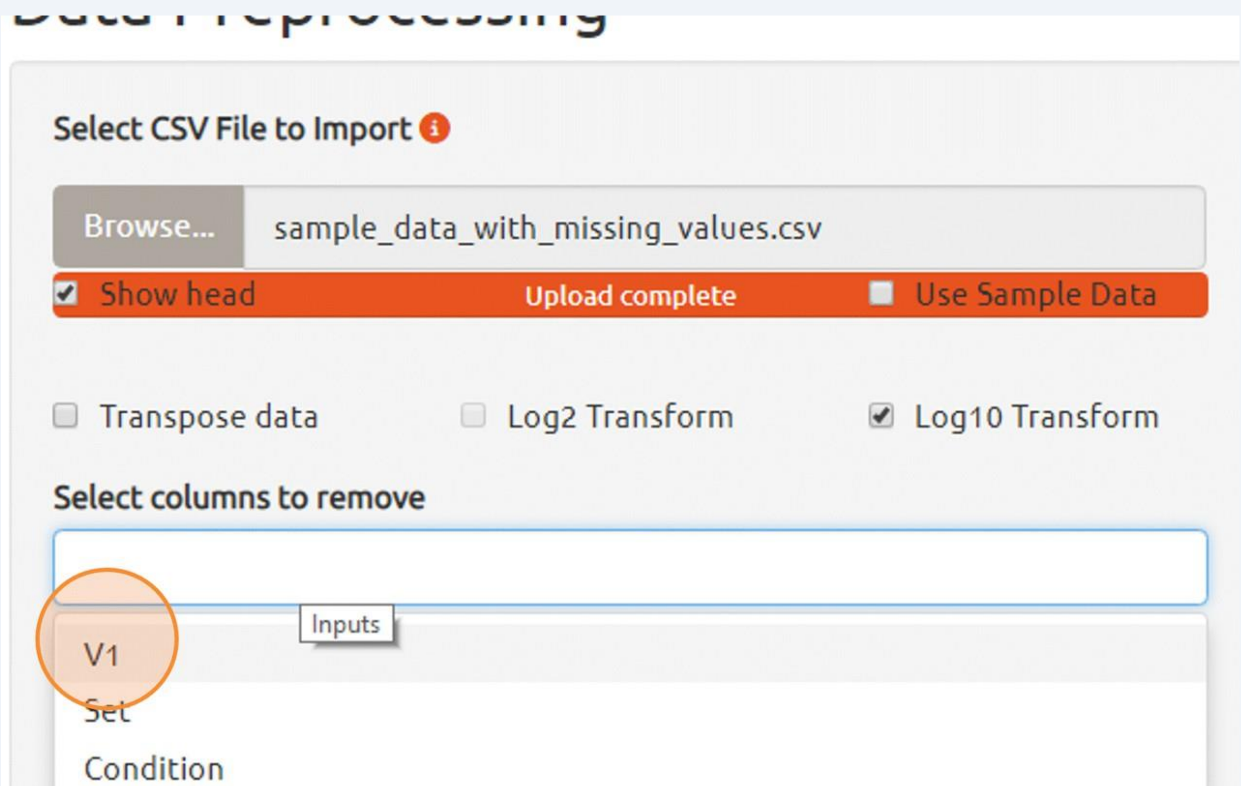

9 Select 'Class' variable to generate quality control plots and "Experimental batch variable" to be used in normalization.

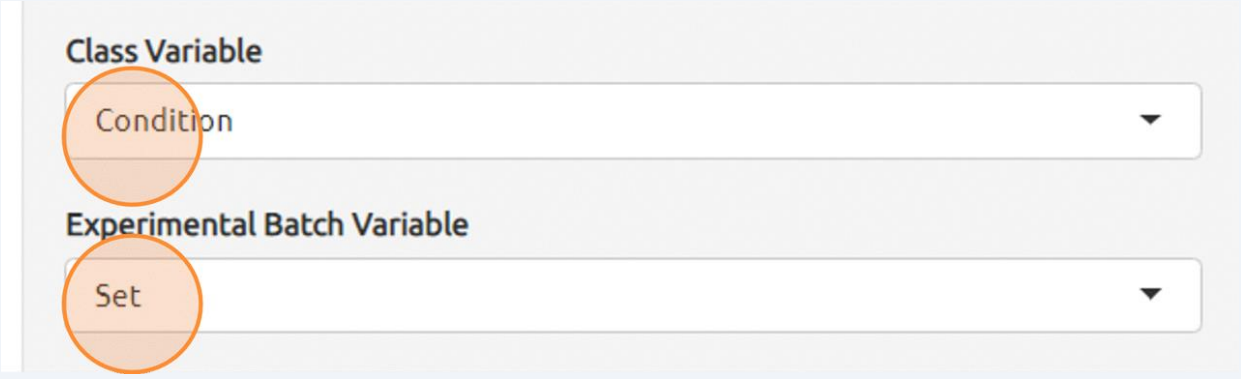

10 Select Data Normalization method

1. Median normalization
2. Quantile normalization
3. Internal Reference Normalization: if selected, select the corresponding internal reference to be used
4. Variance stabilization normalization

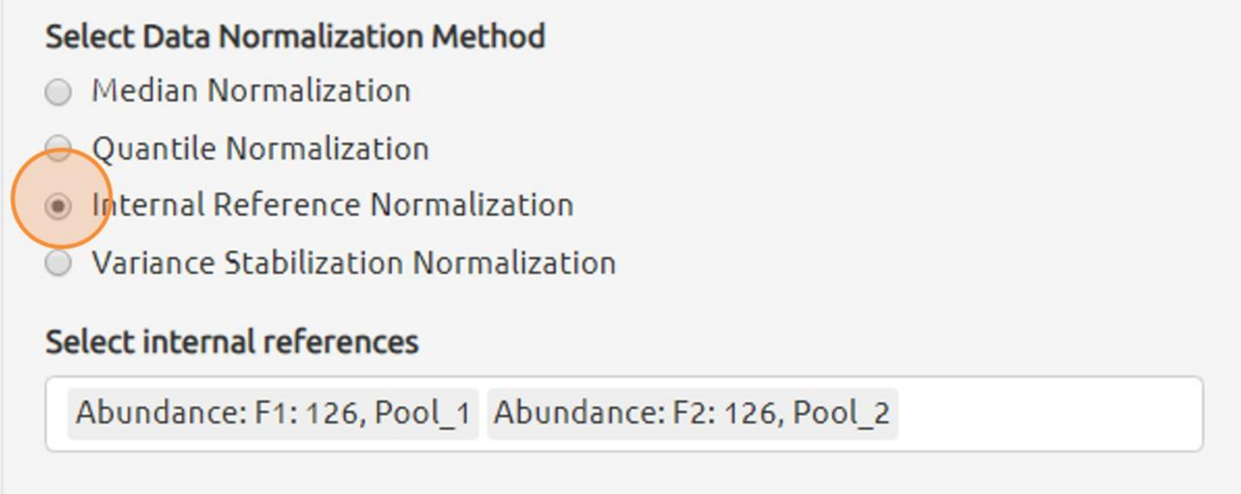

11 Select missing value threshold. Default is 0.5 which means proteins with 50% or less missing values will be considered for imputation.

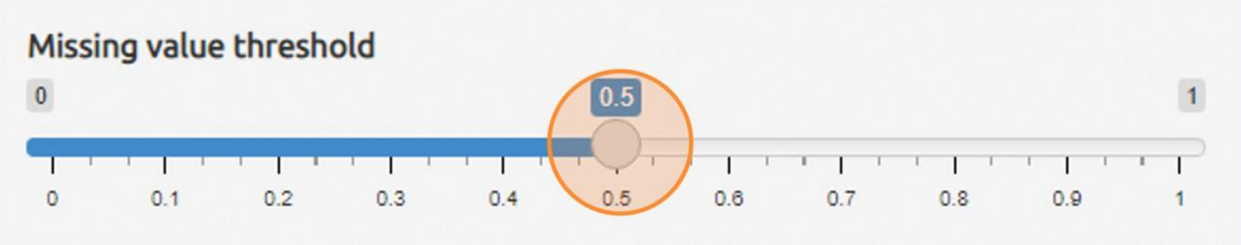

12 Select data imputation method. This include widely used imputation methods,

1. Random draw from a normal distribution
2. K-nearest neighbour imputation (**Hastie, Trevor, et al., 1999**)
3. MissForest imputation: (**Stekhoven and Peter 2012**)**https://doi.org/10.1093/bioinformatics/btr597**

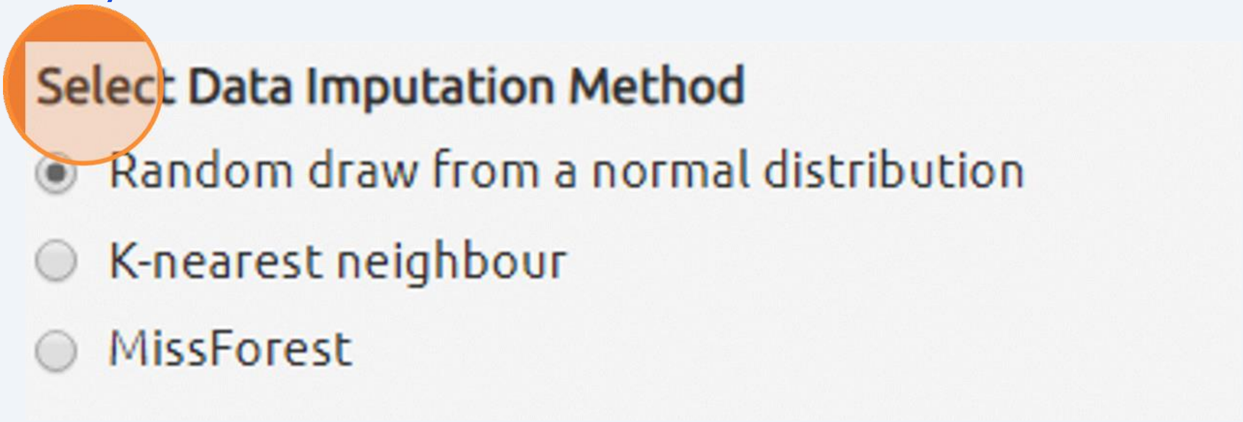

13 Click "Preprocess Data" to start preprocessing.

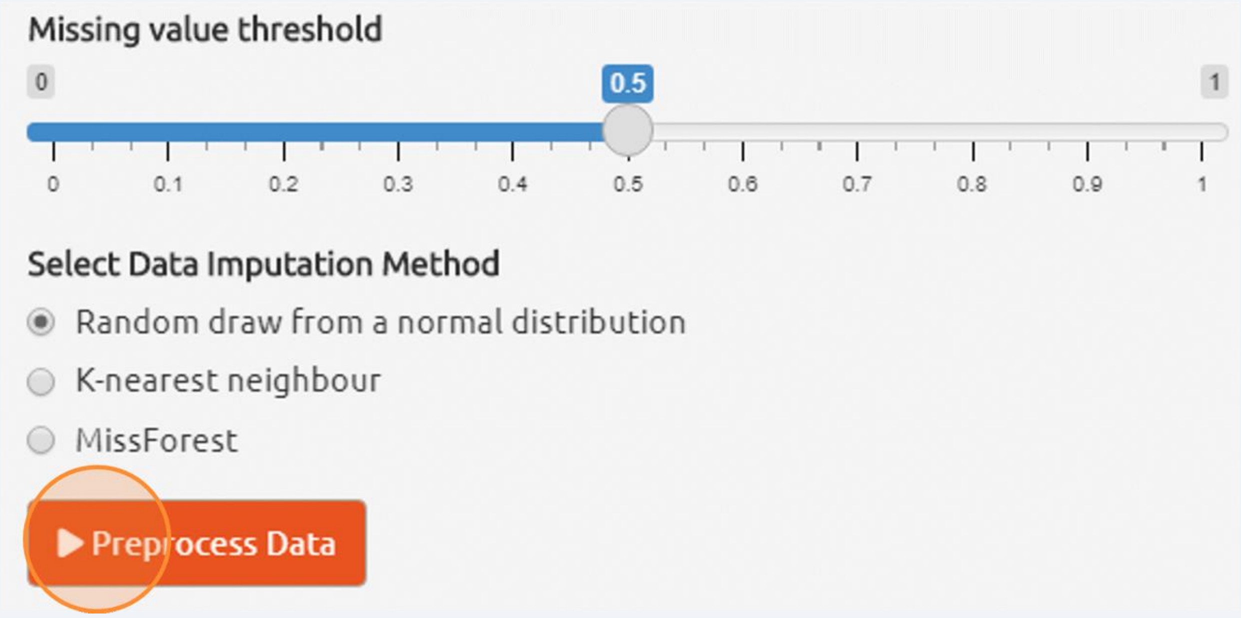

14 The imputed and normalized matrices can be checked and downloaded in "Preprocessed Data" tab.

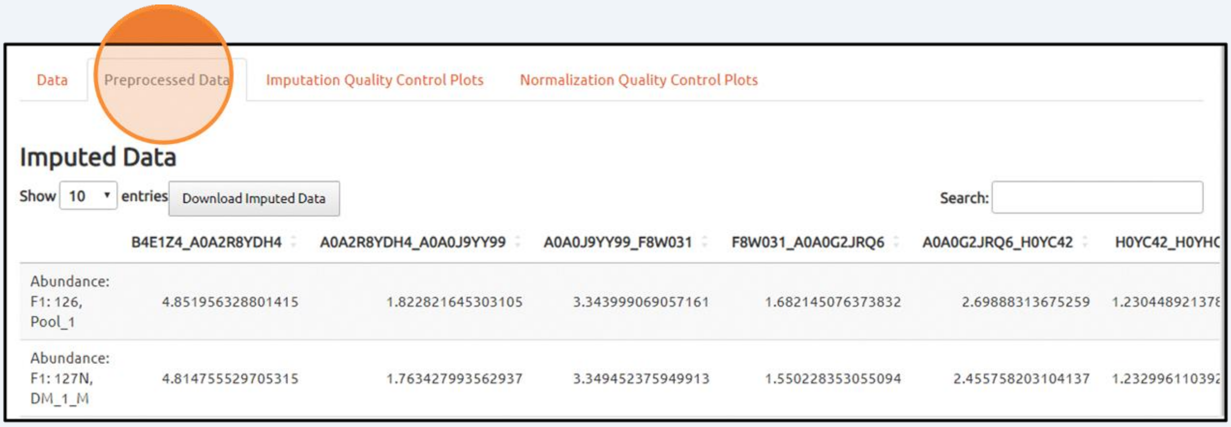

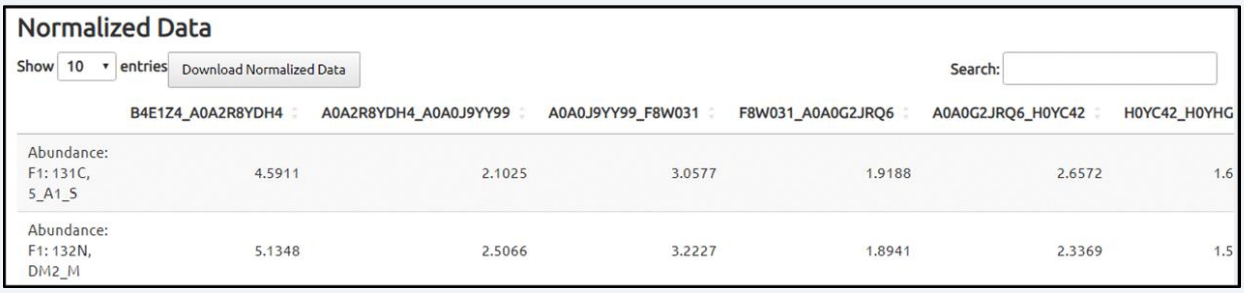

15 "Normalization Quality Control Plots" tab displays, boxplots, density plots and principal component analysis plots before and after normalization.

The coloring is based on the "Class Variable".

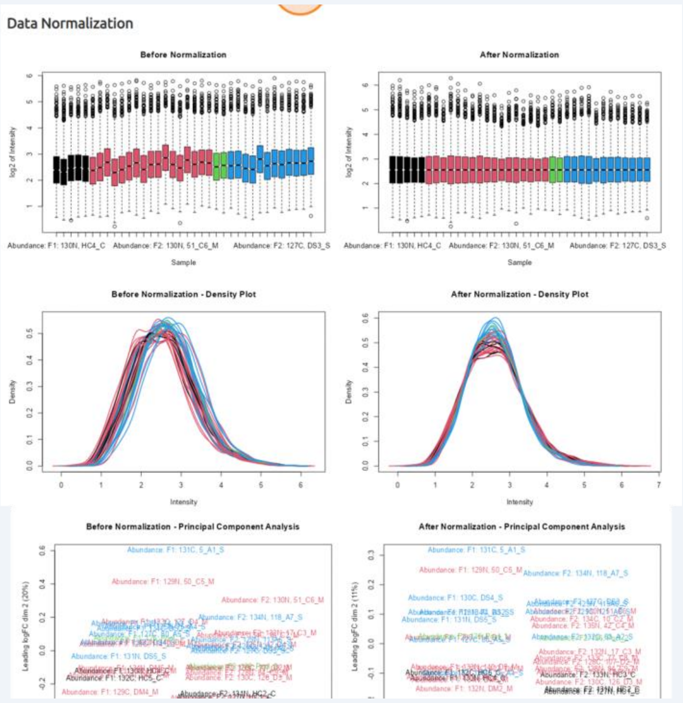

16

"Imputation Quality Control Plots" tab displays, boxplots, density plots and principal component analysis plots before and after imputation.

The coloring is based on the "Class Variable".

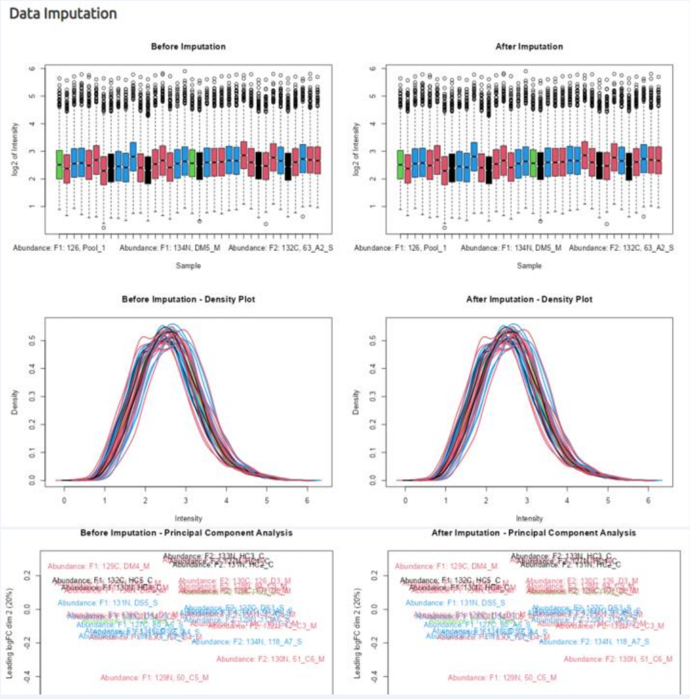

Step-by-step Guide to Perform Differential Expression Analysis using FlexStatv1 Pipeline - Using Sample Data

Scribe

This feature facilitates pairwise differential expression analysis with integrated multiple-testing corrections. Users have the option to filter results by configuring cutoffs for log fold change and p-values.

This functionality includes visual representations of differential expression results, including boxplots, volcano plots, and heat maps.

Protein type-specific principal component analysis is a prominent aspect of this feature.

This tutorial is based on the sample data provided with the application.

1 Navigate to **https://jglab.shinyapps.io/flexstatv1-pipeline-only/**

2 Go to the "Differental Expression" tab.

3 Click the "Use Sample Data" field.

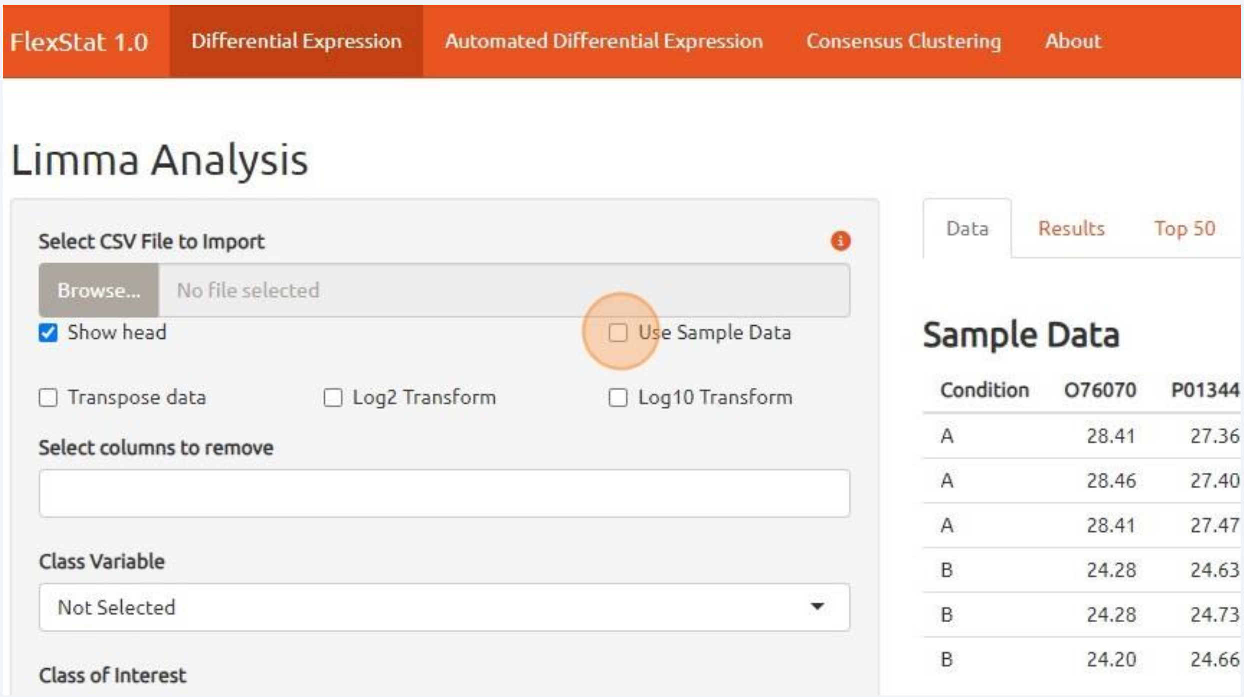

4 Select Class variable as "Condition"

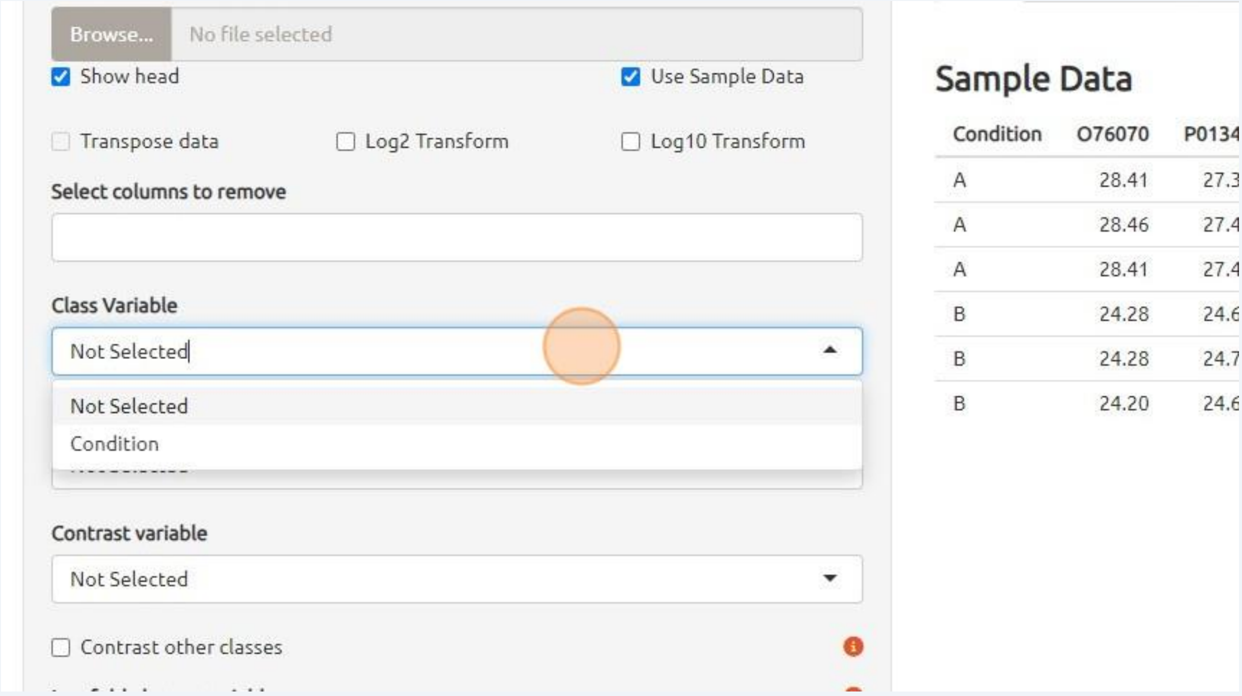

5 Select class of interest from the dropdown

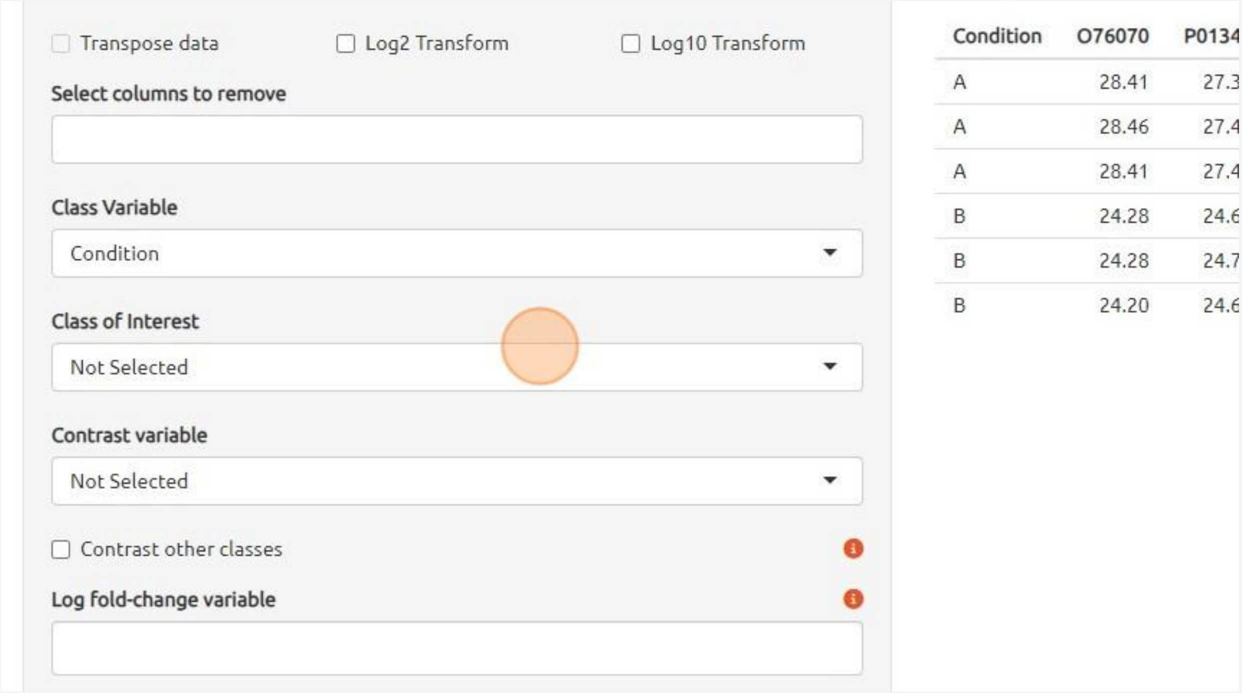

6 Select contrast variable from the dropdown

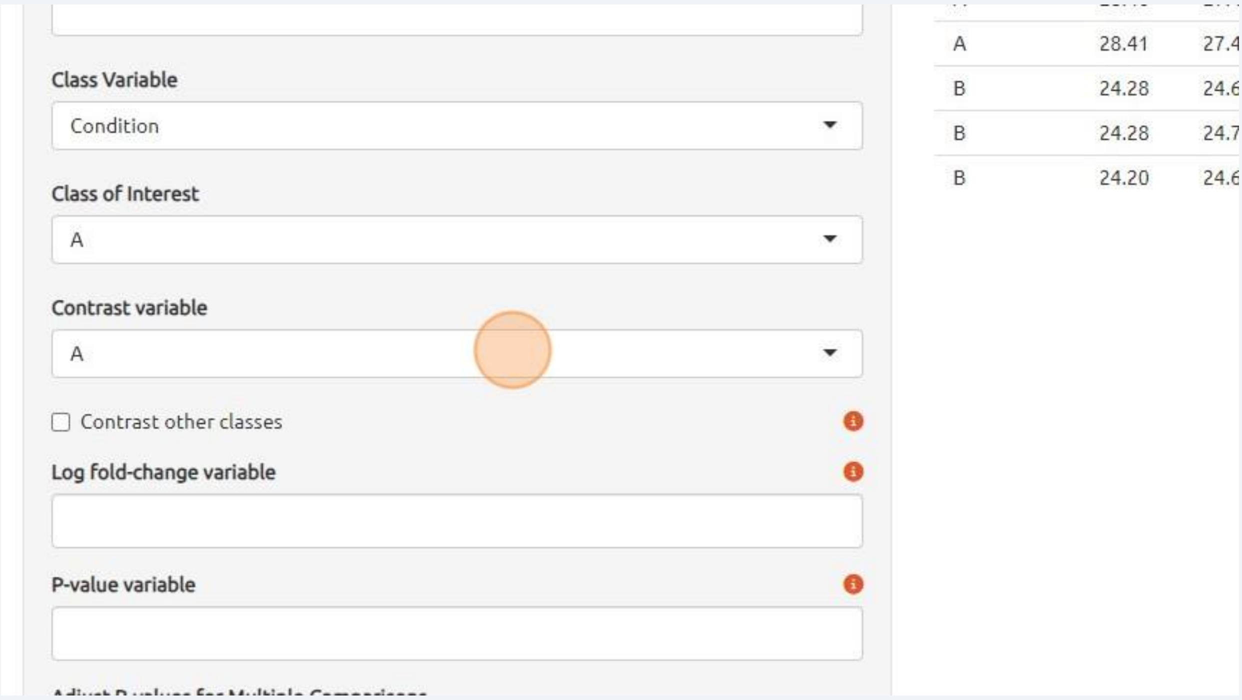

7 **[Optional]** Change log fold change cutoff and p-value cutoff

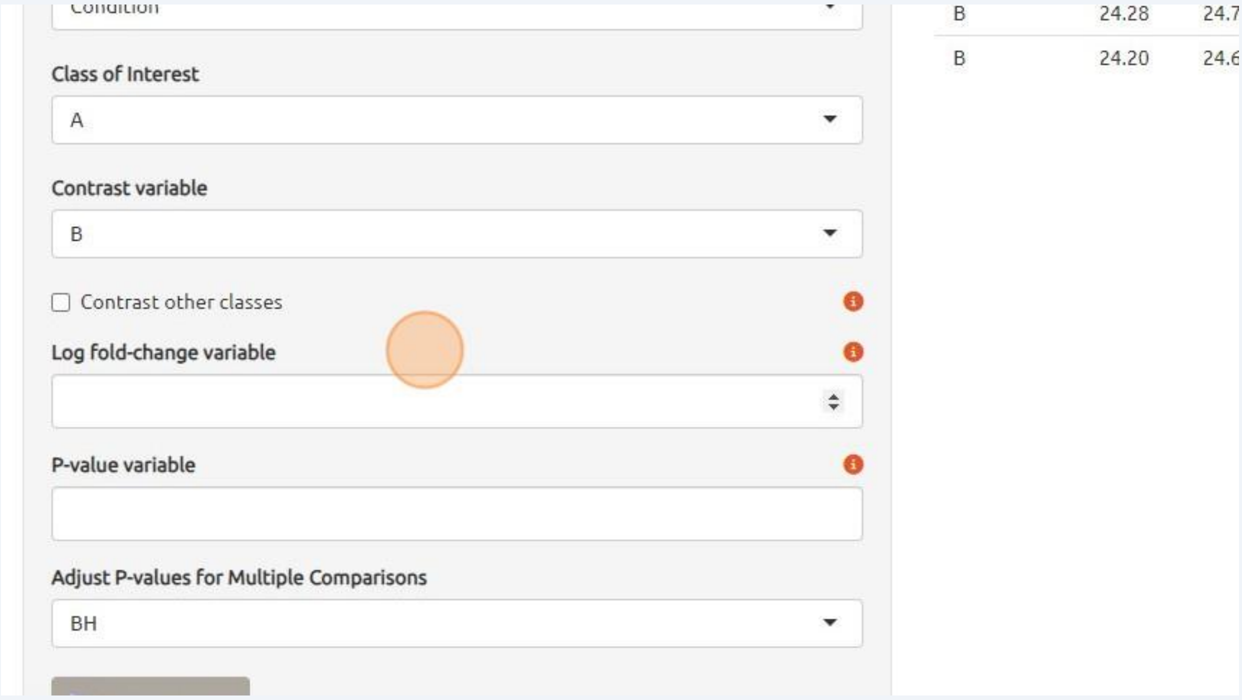

8 Click "Perform Limma"

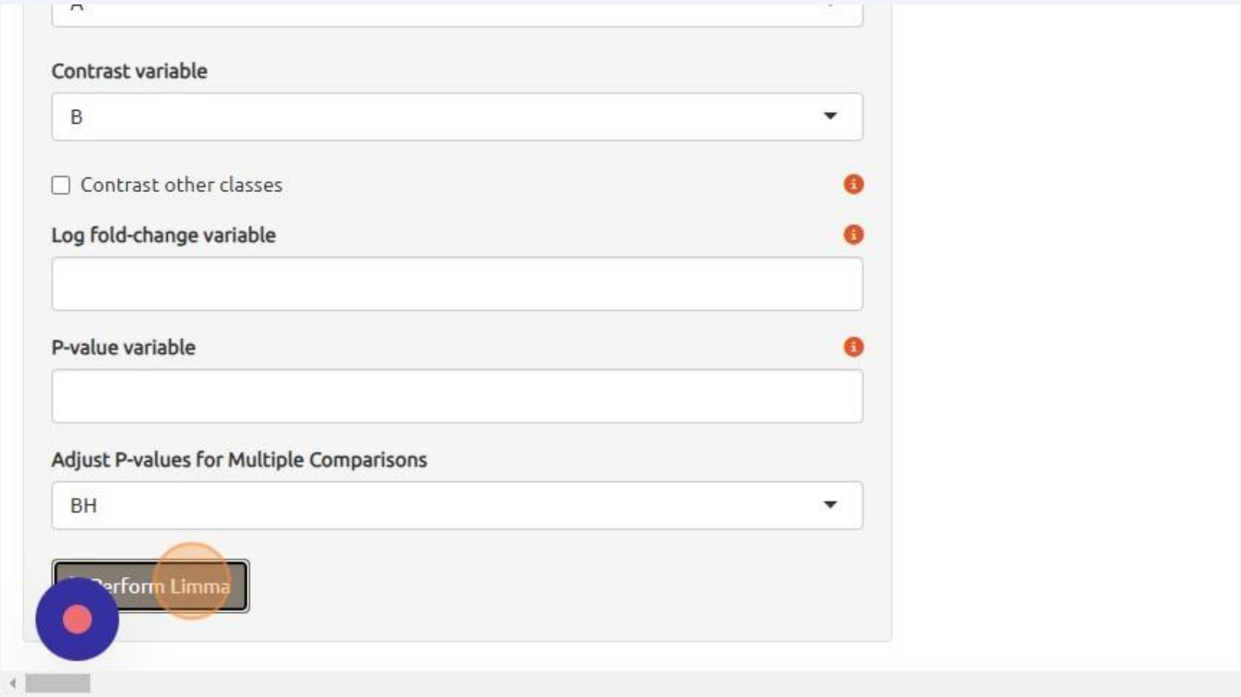

9 Click "Results" tab

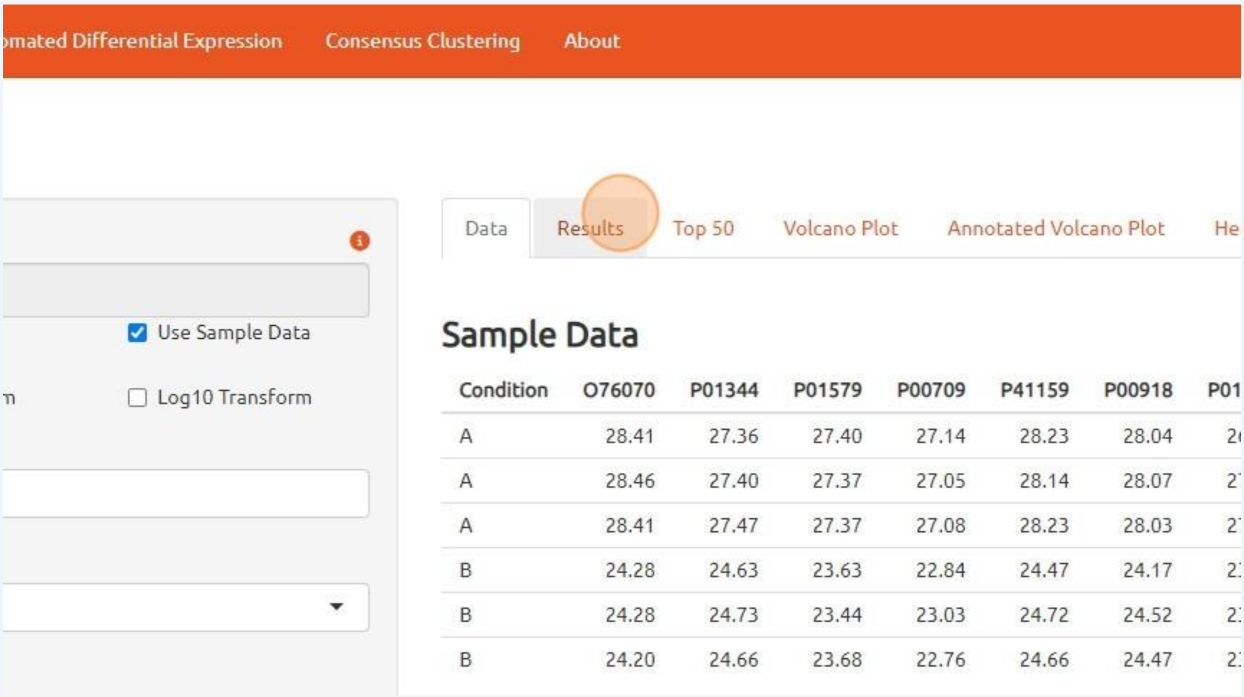

10 Click "Download Current Page" to download the visible results on the current page as a csv file.

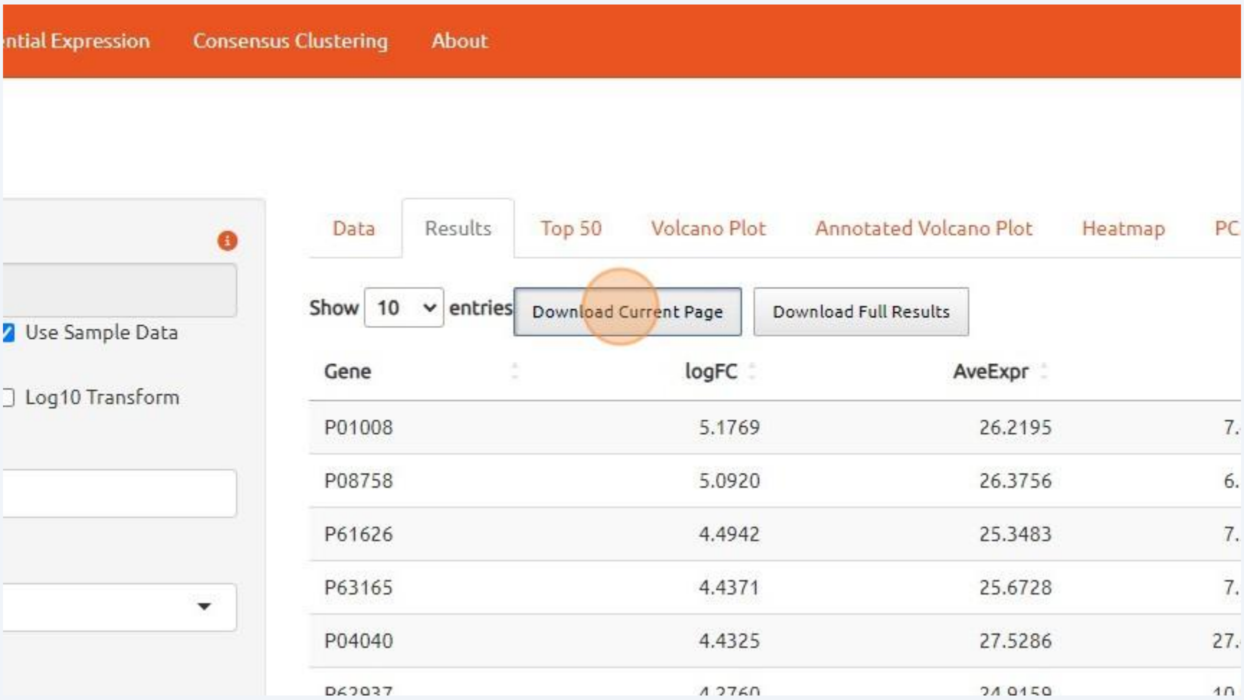

11 Click "Download Full Results" to download all results as a csv file.

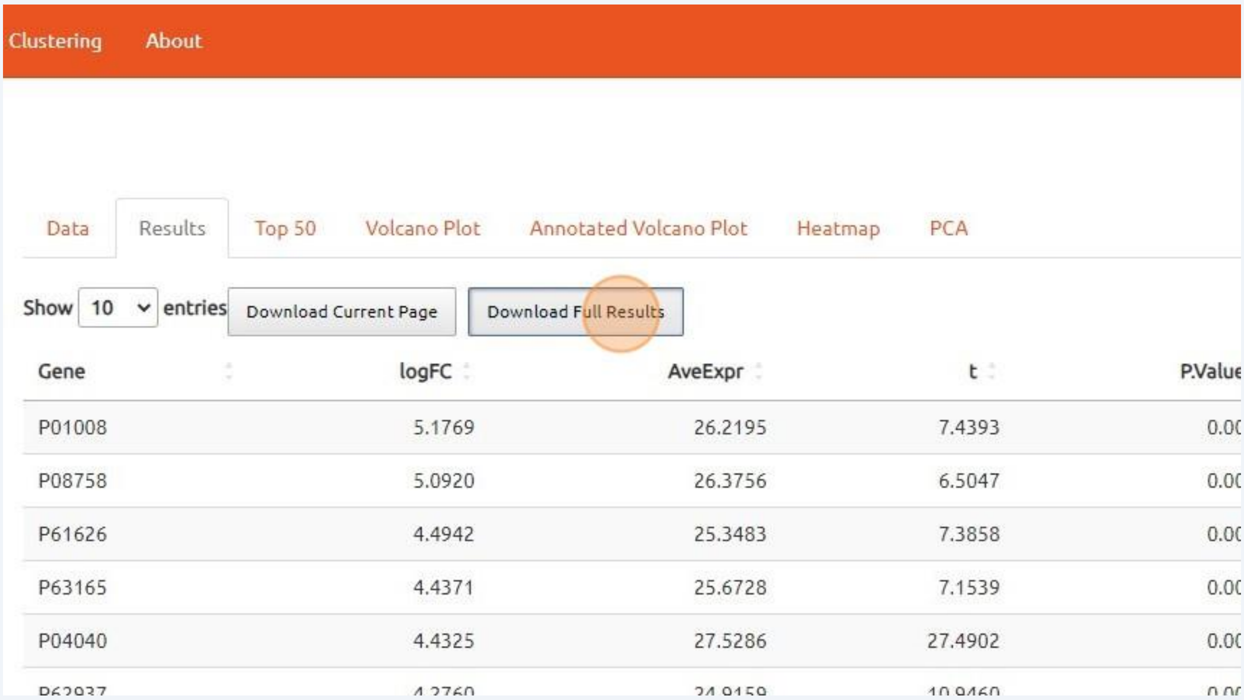

12 Click "Top 50" to see the highly varied 50 proteins/genes between the classes

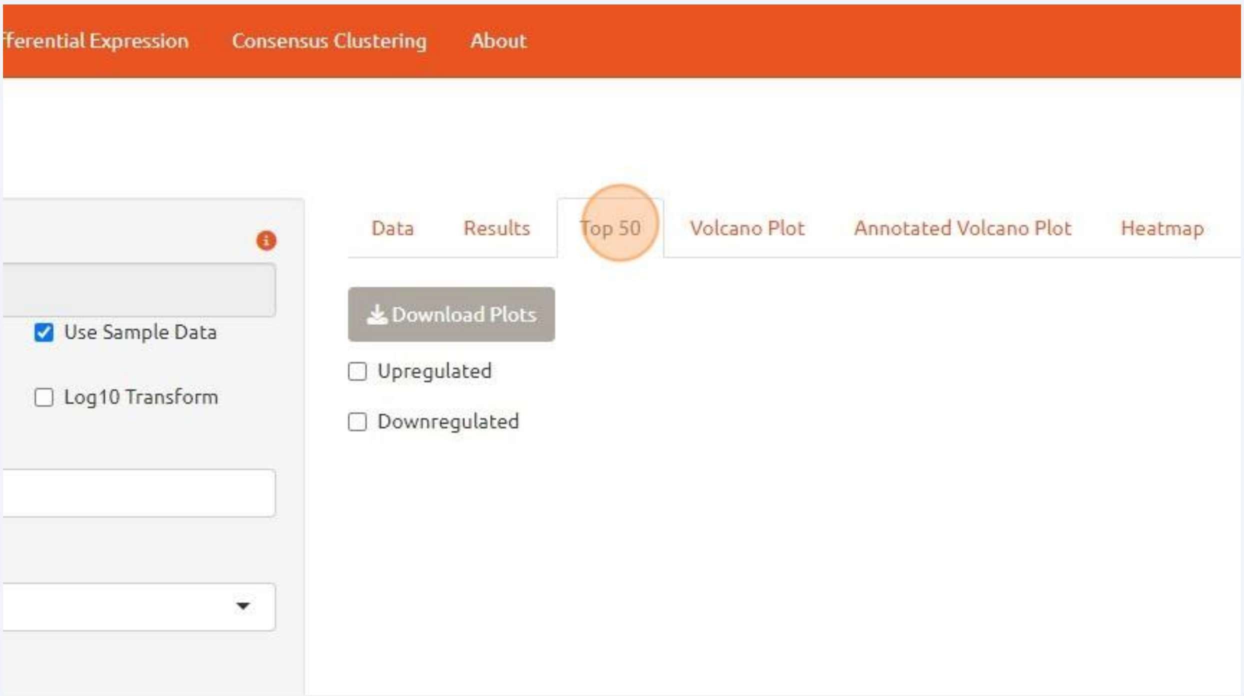

13 Click "Upregulated" to filter the boxplots of upregulated proteins/genes between the classes

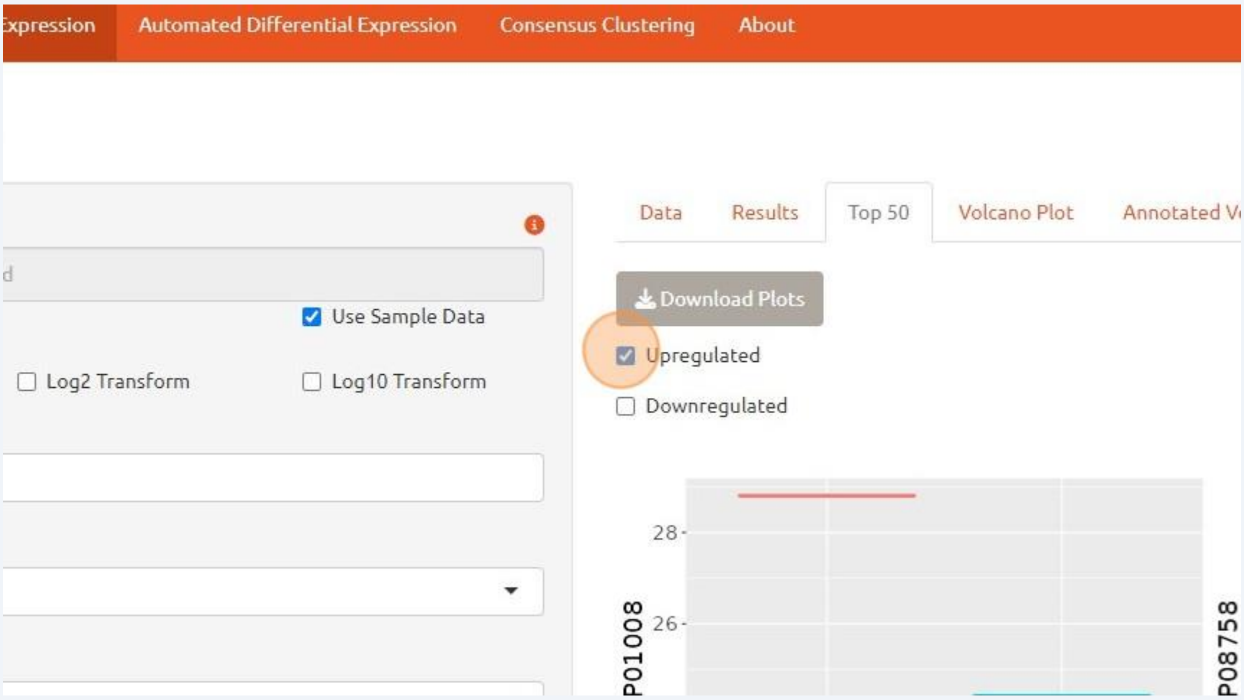

14 Click "Downregulated" to filter the boxplots of upregulated proteins/genes between the classes

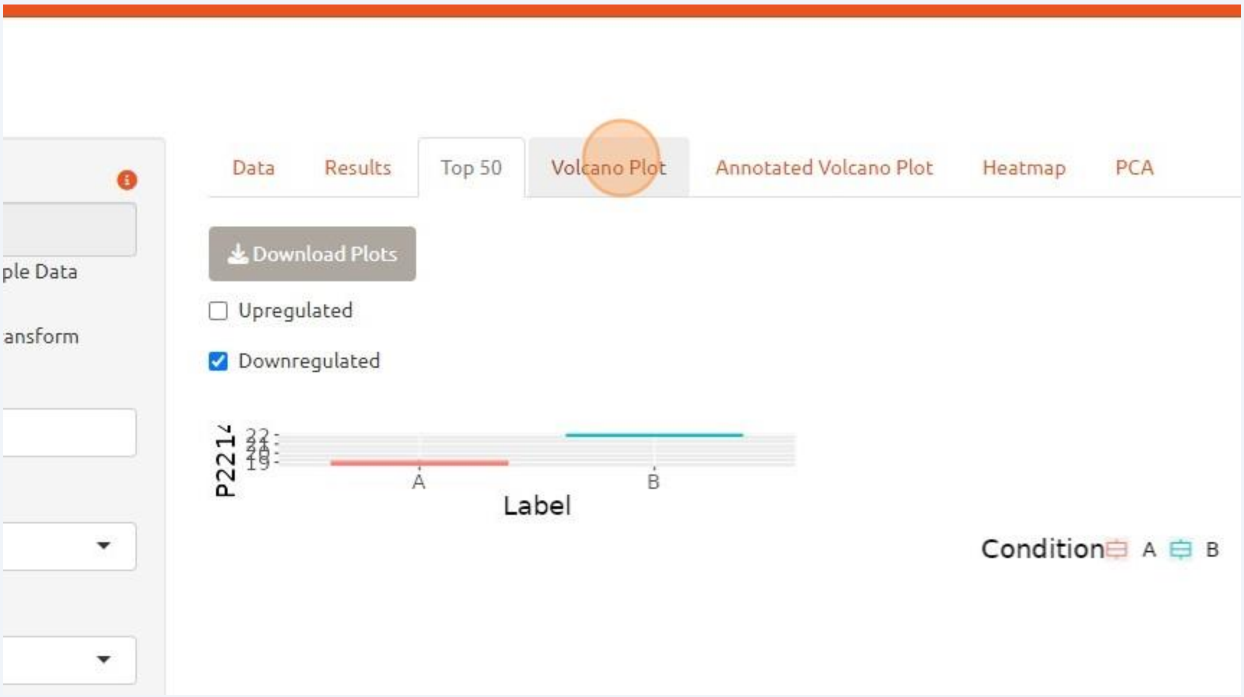

15 Click "Volcano Plot" to check the draft volcano plot

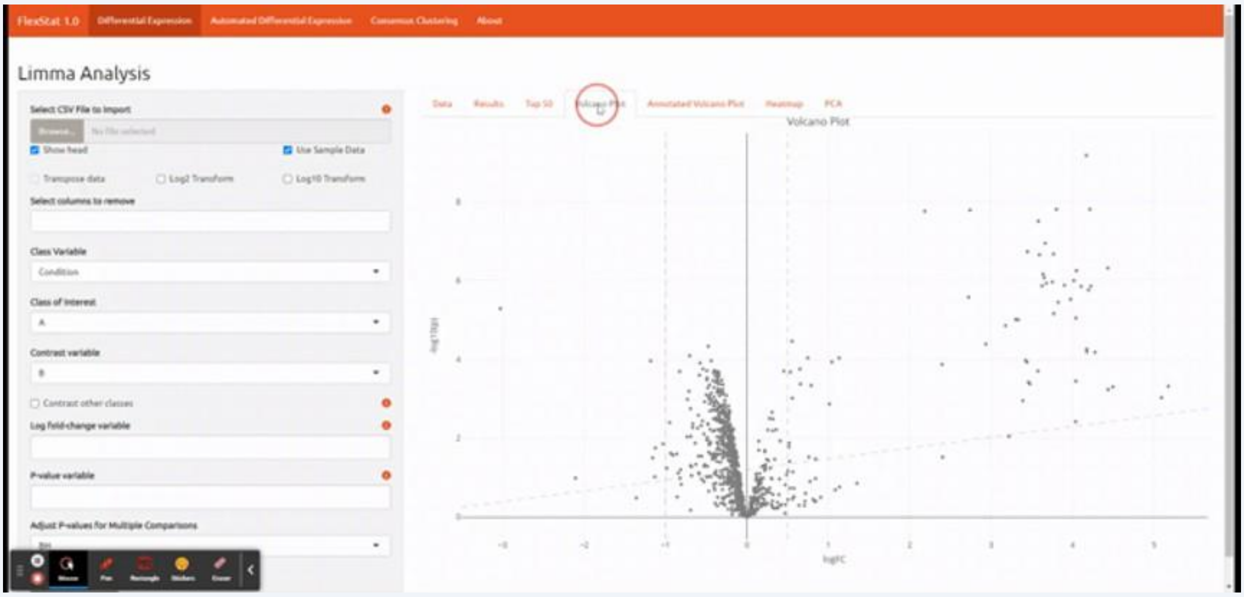

16 Click "Annotated Volcano Plot" to obtain the well-annotated points showing significant proteins/genes between the conditions.

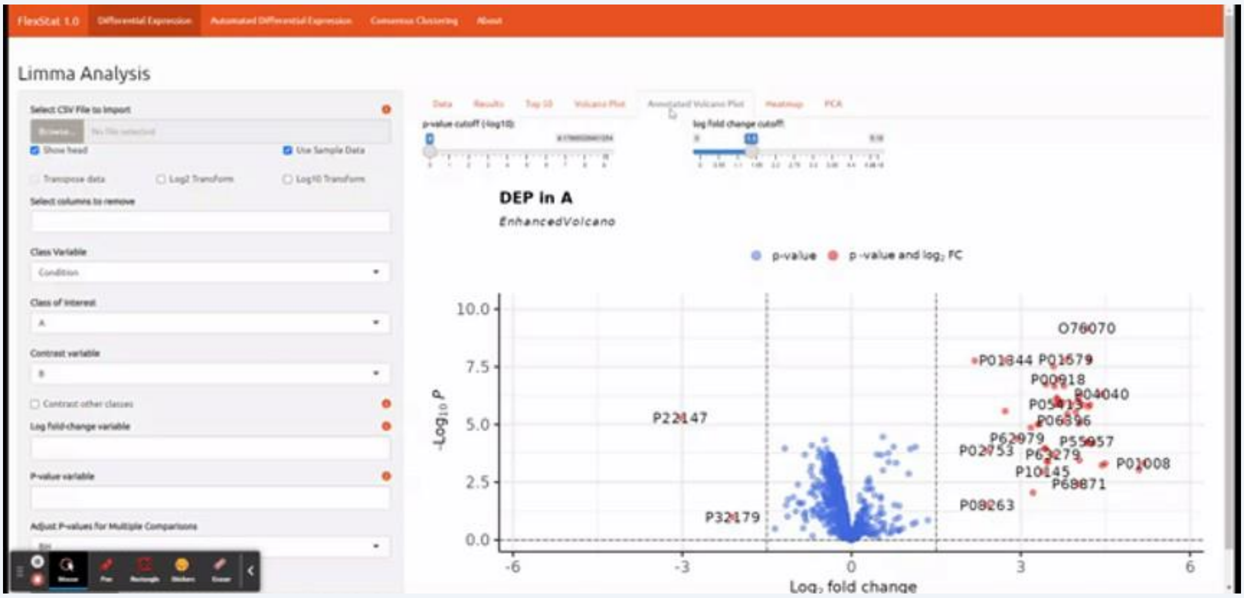

17 **[Optional]** Change p-value cutoff based on your research requirements.

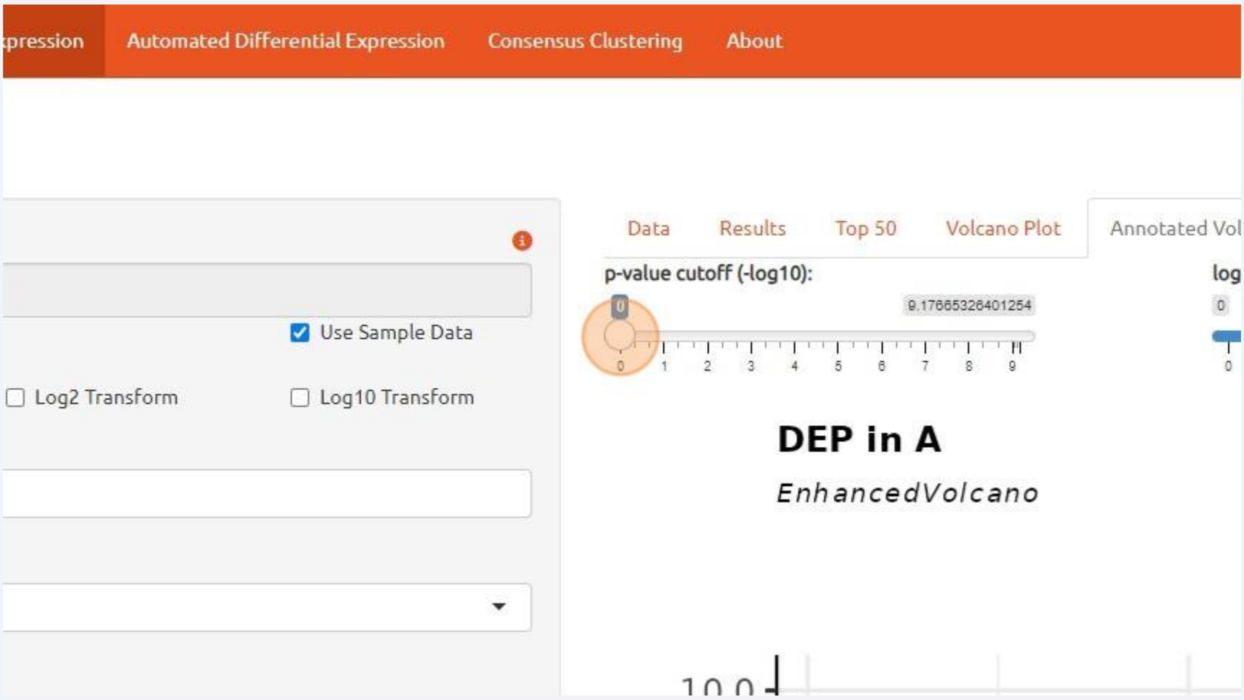

18 **[Optional]** Change log fold-change cutoff based on your research requirements.

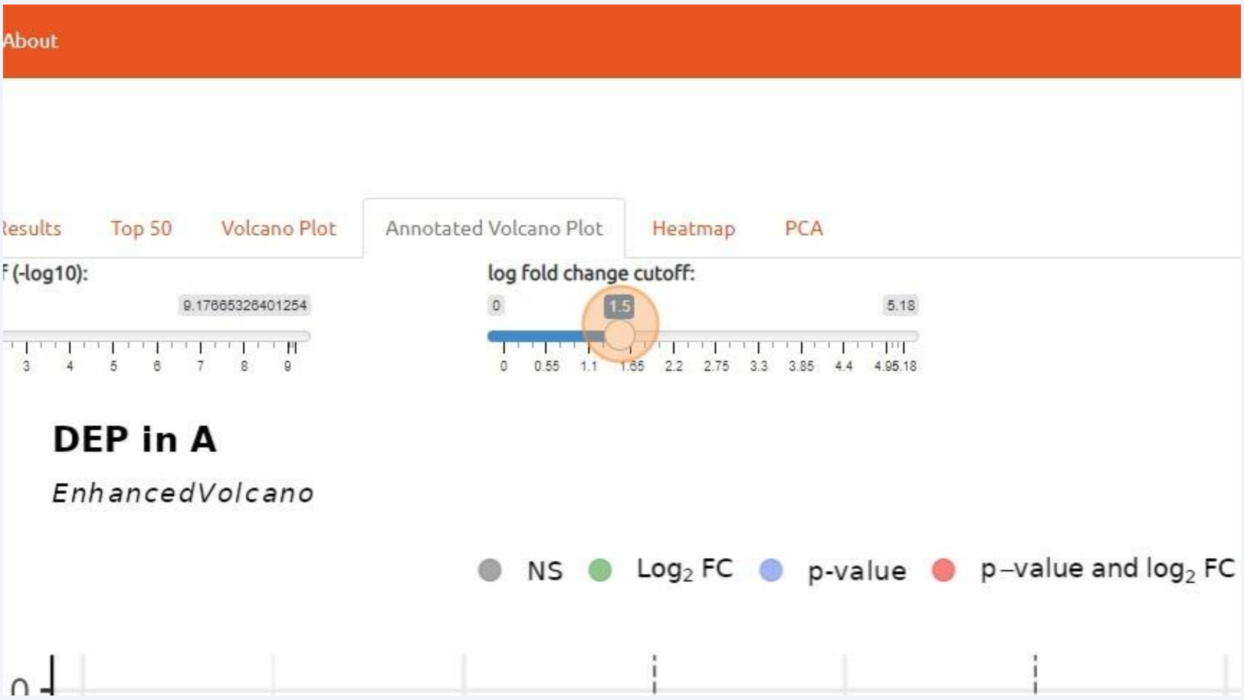

19 Click "Heatmap" to obtain the heatmap

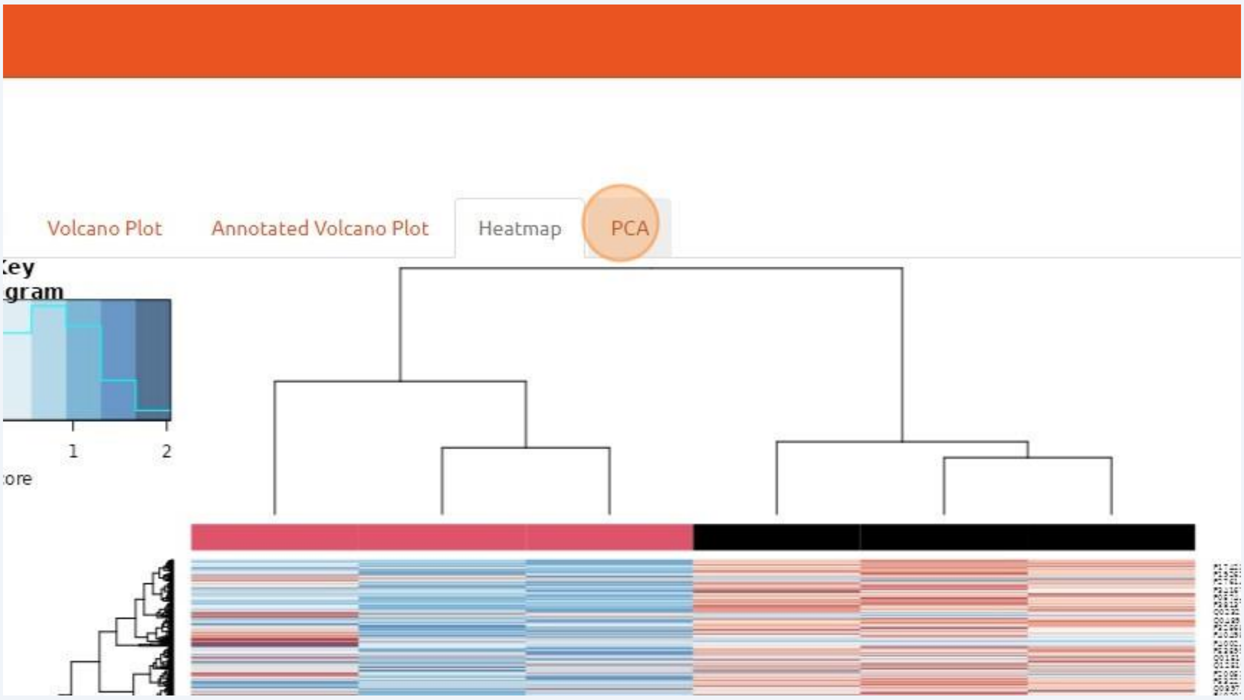

Step-by-step Guide to Perform Differential Expression Analysis using FlexStatv1 Pipeline - Upload experimental results

Scribe

This feature facilitates pairwise differential expression analysis with integrated multiple-testing corrections. Users have the option to filter results by configuring cutoffs for log fold change and p-values.

This functionality includes visual representations of differential expression results, including boxplots, volcano plots, and heat maps.
Protein type-specific principal component analysis is a prominent aspect of this feature.

This tutorial is based on uploading an experimentally generated protein expression profile into the application.

1 Navigate to https://jglab.shinyapps.io/flexstatv1-pipeline-only/

2 Go to "Differential Expression" tab.

3 Prepare data having rows as the samples and protein names in the columns and having a column for experimental condition/class as shown in the right-side panel.

Sample Data

Condition	O76070	P01344	P01579	P00709	P41159	P00918	P01112	Q15843	P10636
A	28.41	27.36	27.40	27.14	28.23	28.04	26.95	25.87	29.26
A	28.46	27.40	27.37	27.05	28.14	28.07	27.15	25.68	29.32
A	28.41	27.47	27.37	27.08	28.23	28.03	27.15	25.49	29.24
B	24.28	24.63	23.63	22.84	24.47	24.17	23.46	22.01	25.93
B	24.28	24.73	23.44	23.03	24.72	24.52	23.76	21.78	25.56
B	24.20	24.66	23.68	22.76	24.66	24.47	23.70	21.95	25.55

4 Click "Browse..." and upload your file

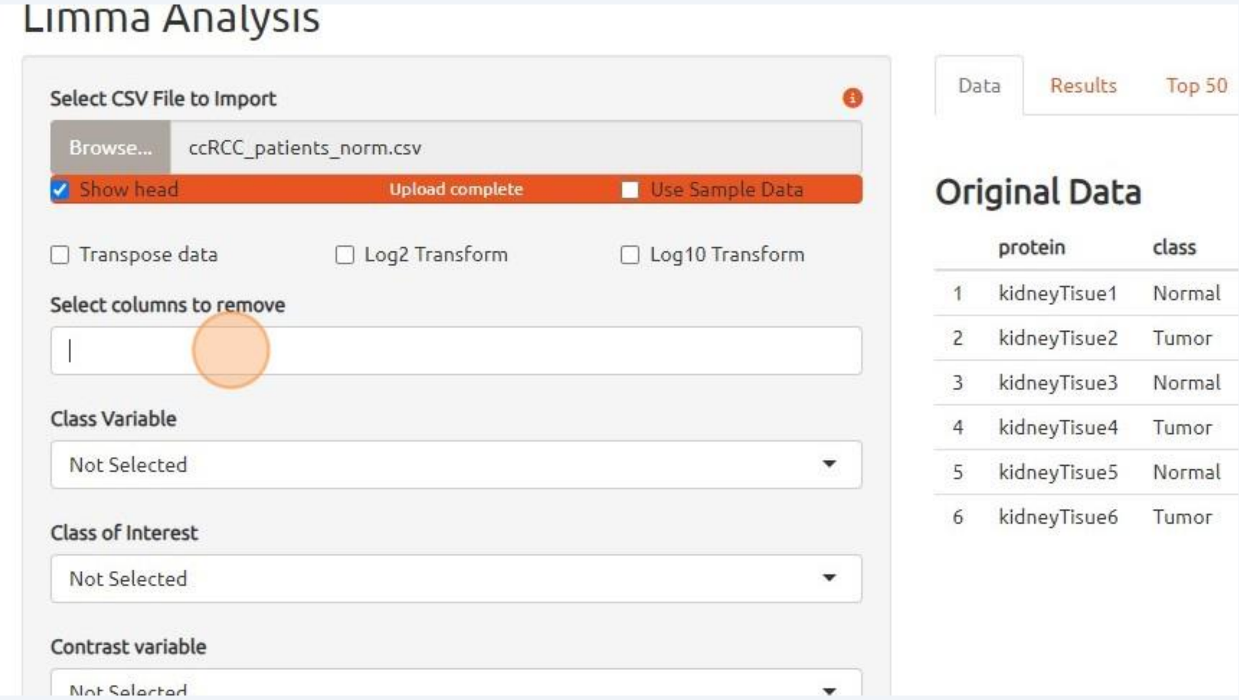

5 Select unwanted columns to be removed.

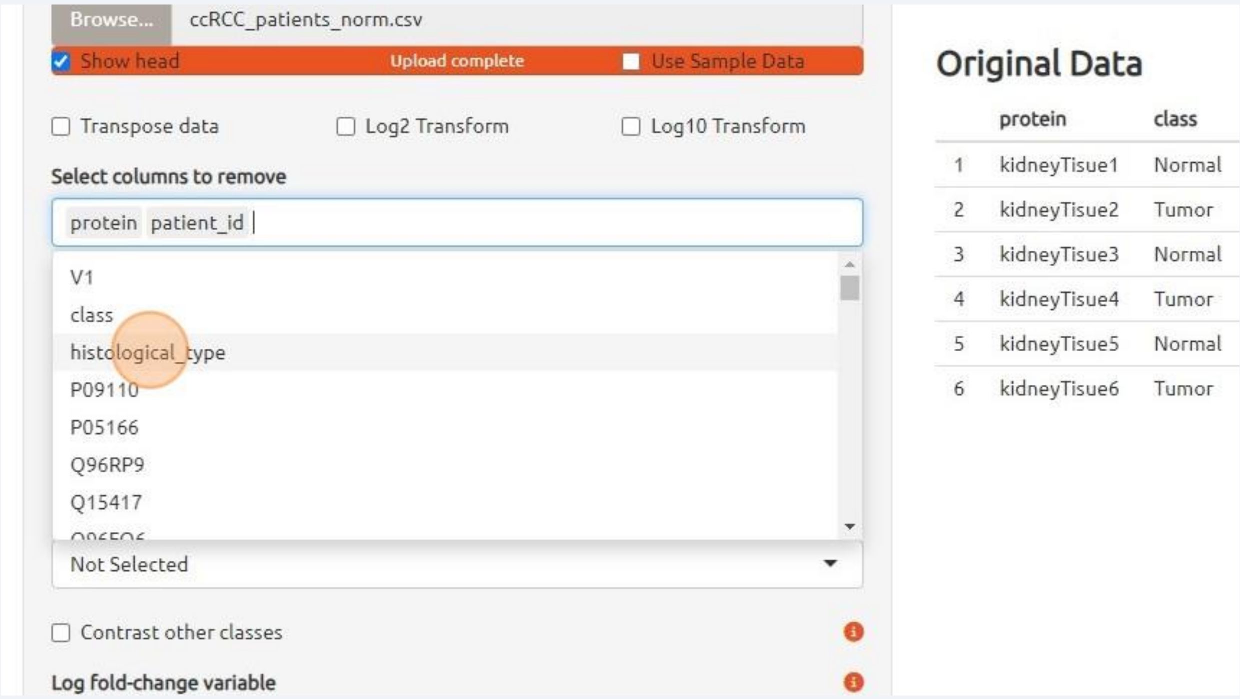

6 Select Class variable from the dropdown

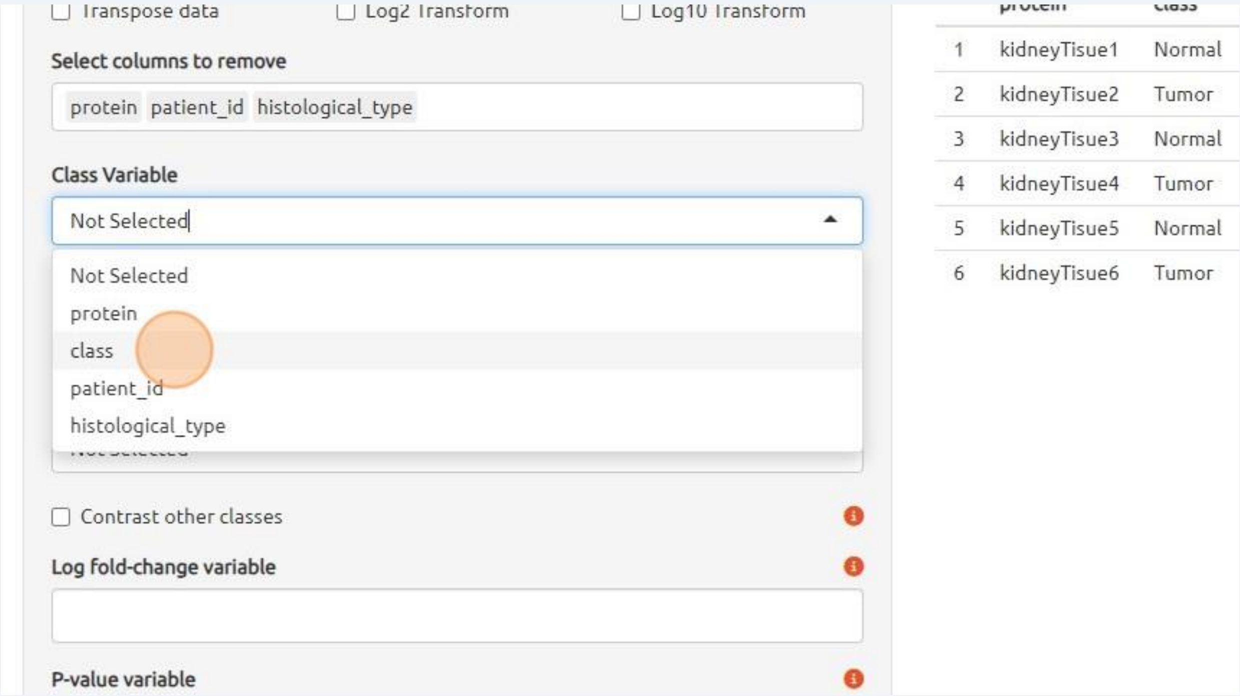

7 Select Contrast variable from the dropdown

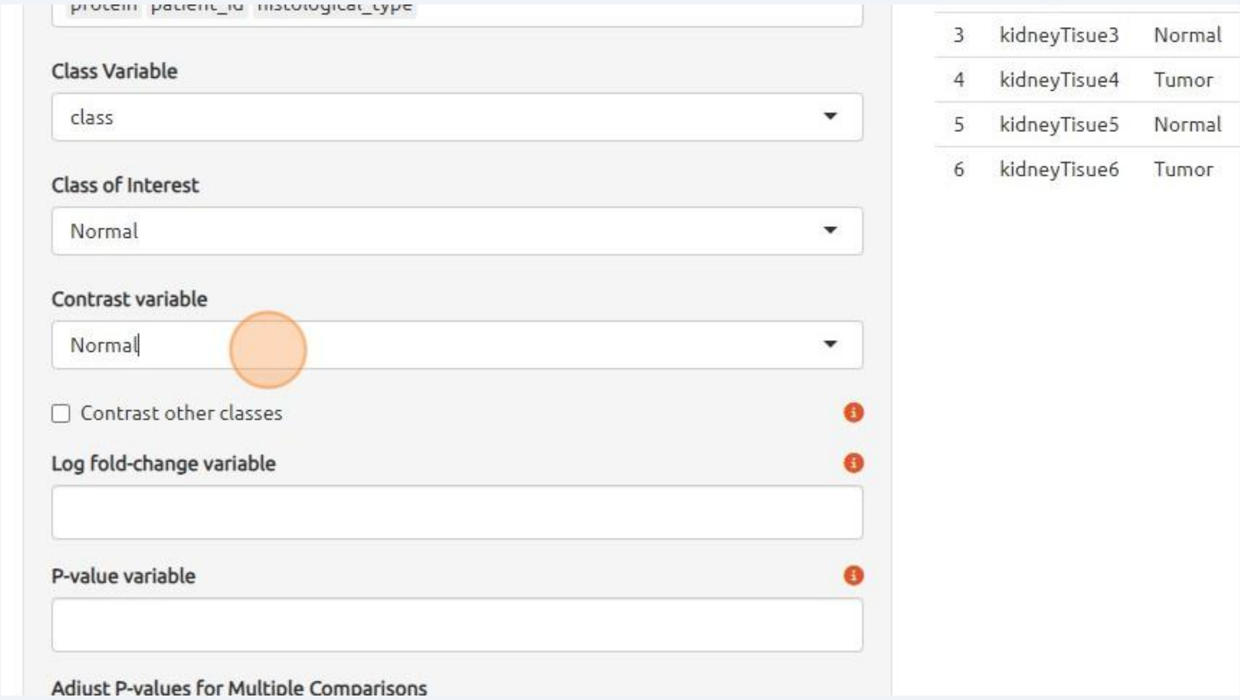

8 Click "Perform Limma"

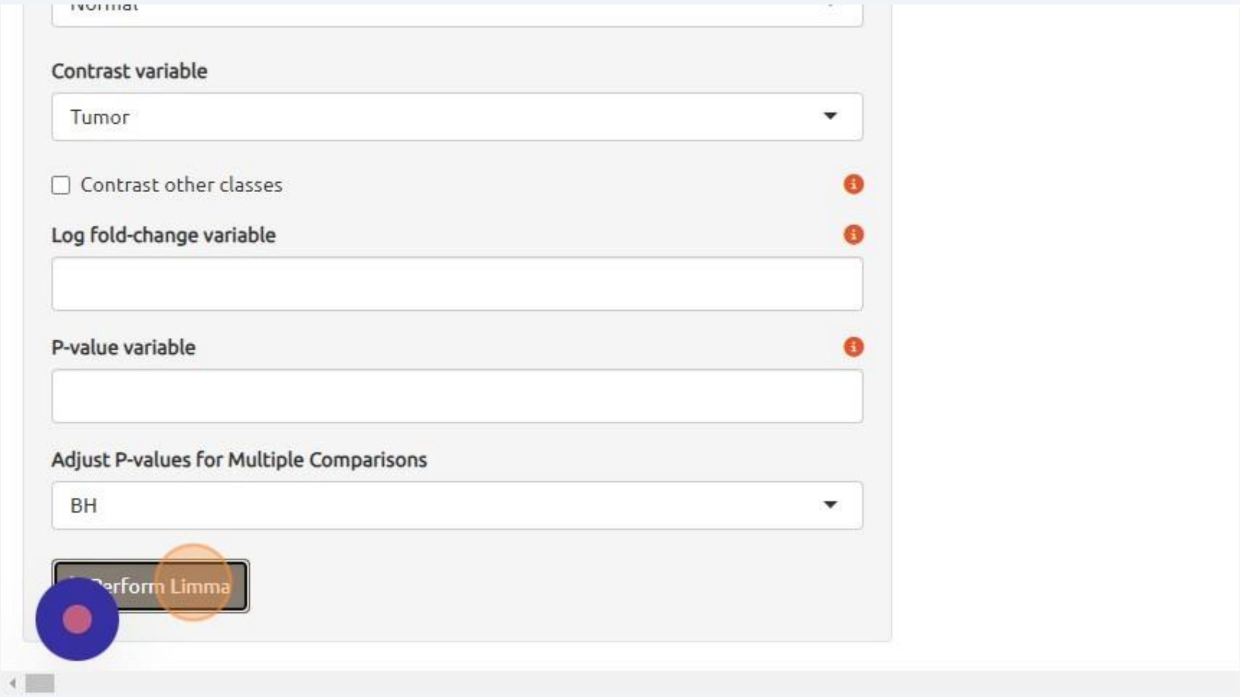

9 Click "Results"

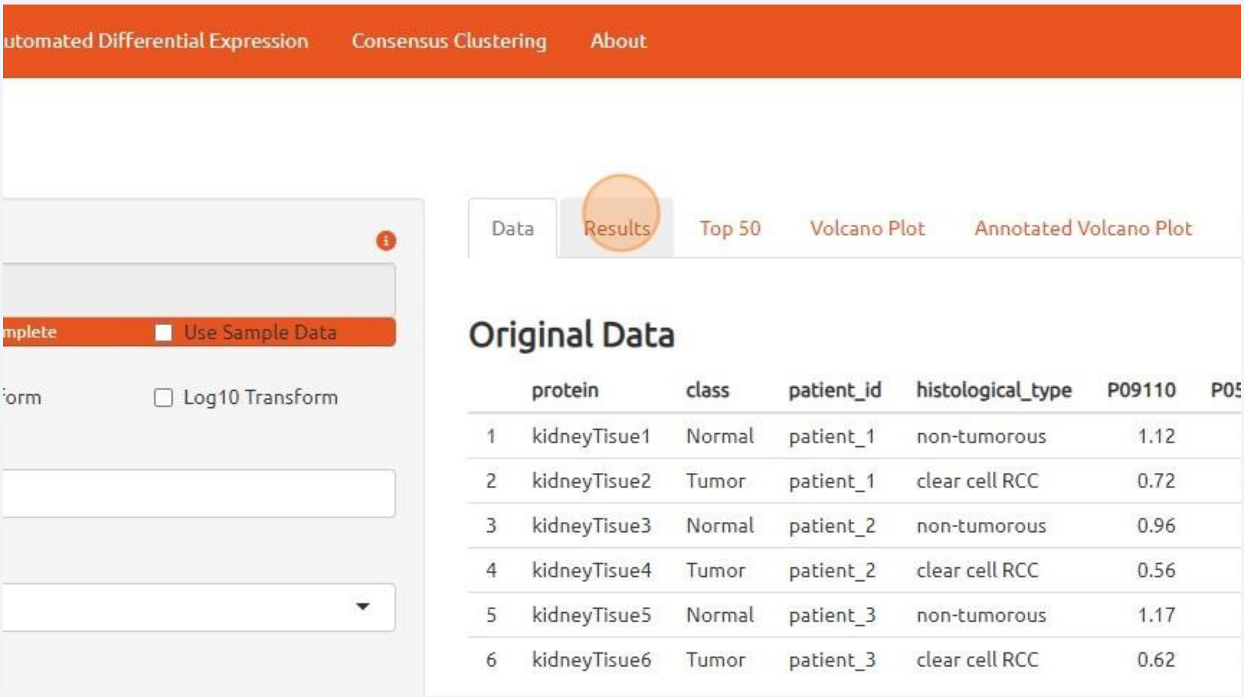

10 Obtain volcano plots from the "Volcano plot" and "Annotated Volcano plot" tabs

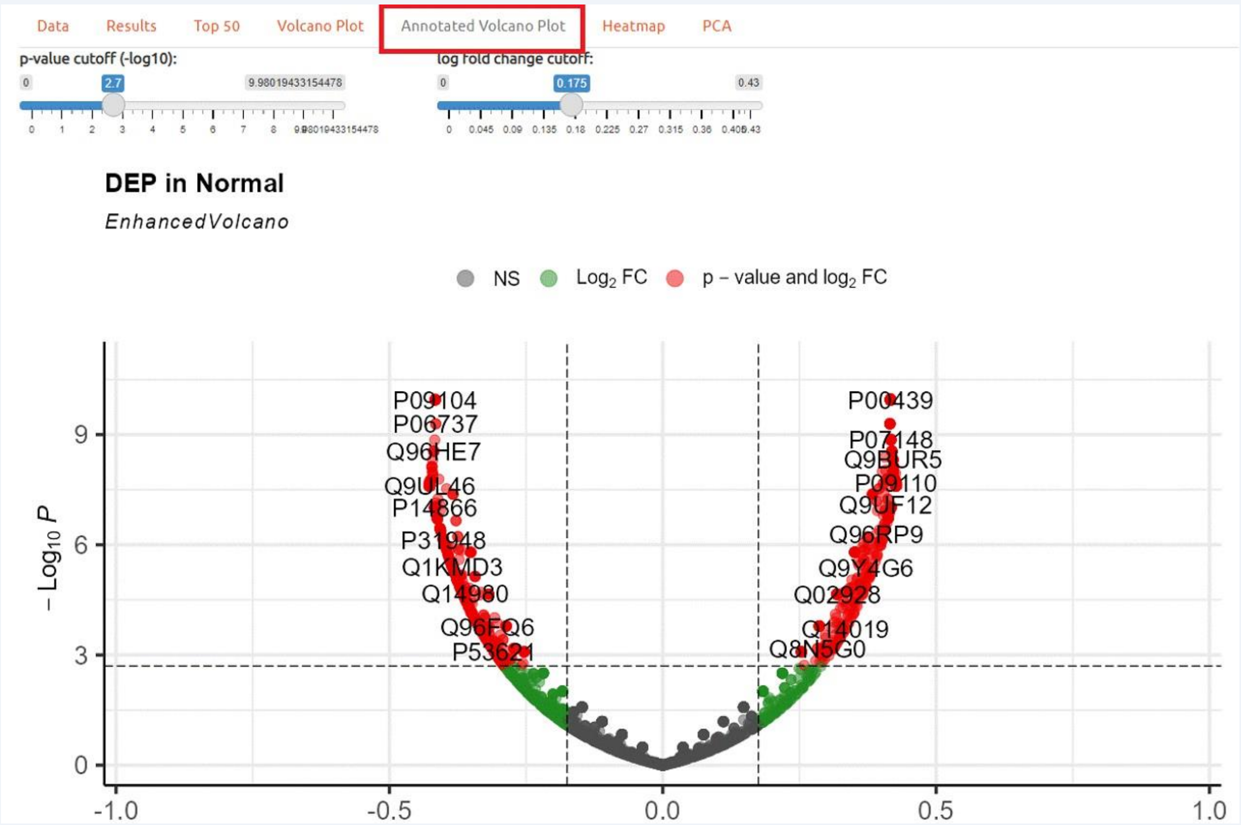

11 Obtain heatmap from the "Heatmap" tab.

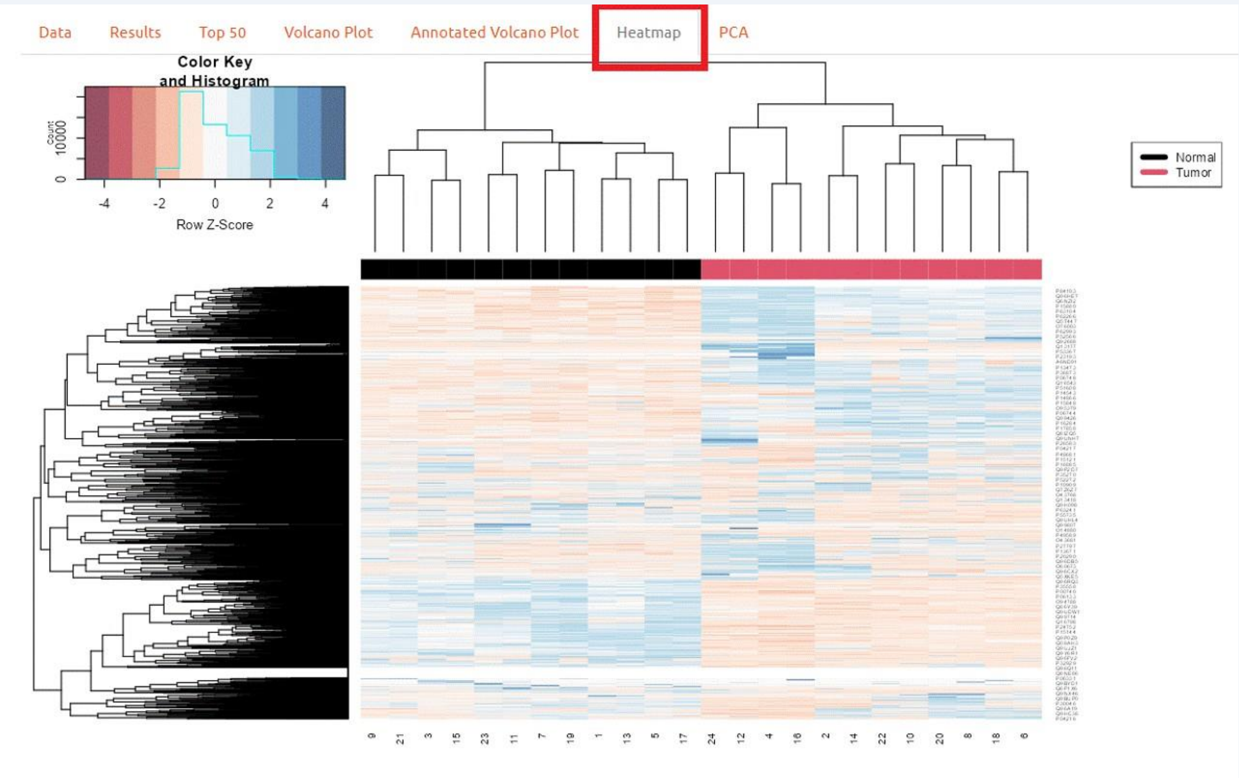

12 Click "PCA"

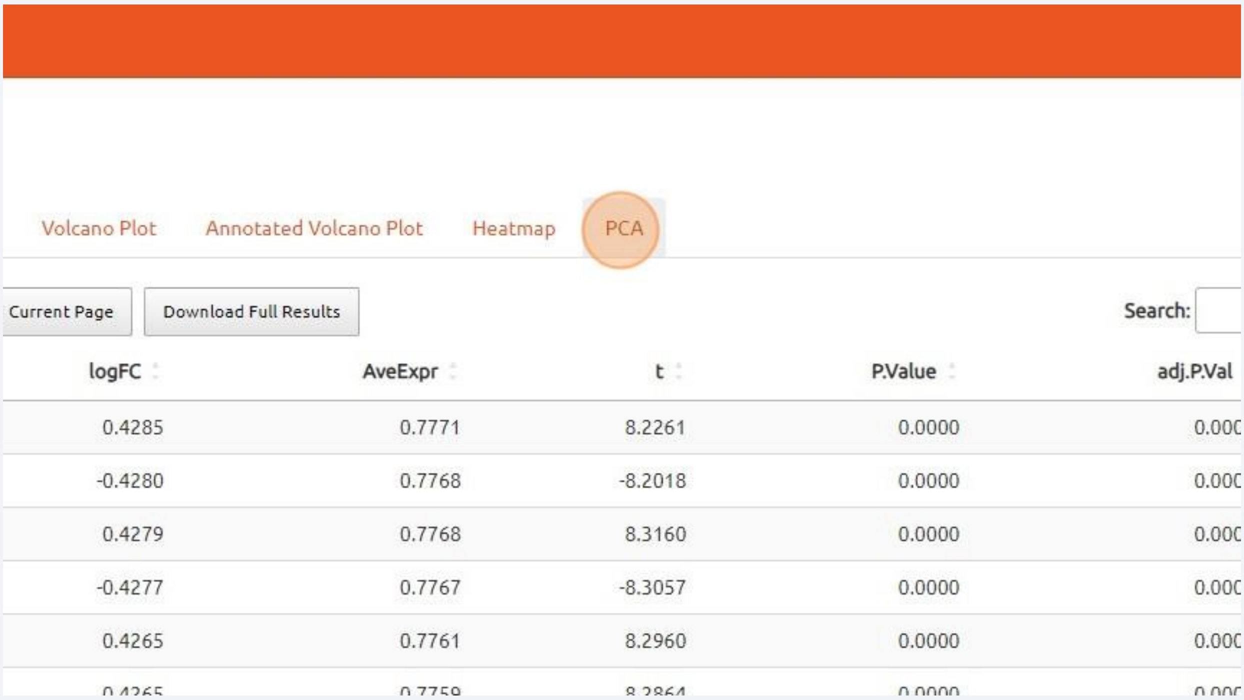

13 Click "Kinases" to filter PCA with kinases.

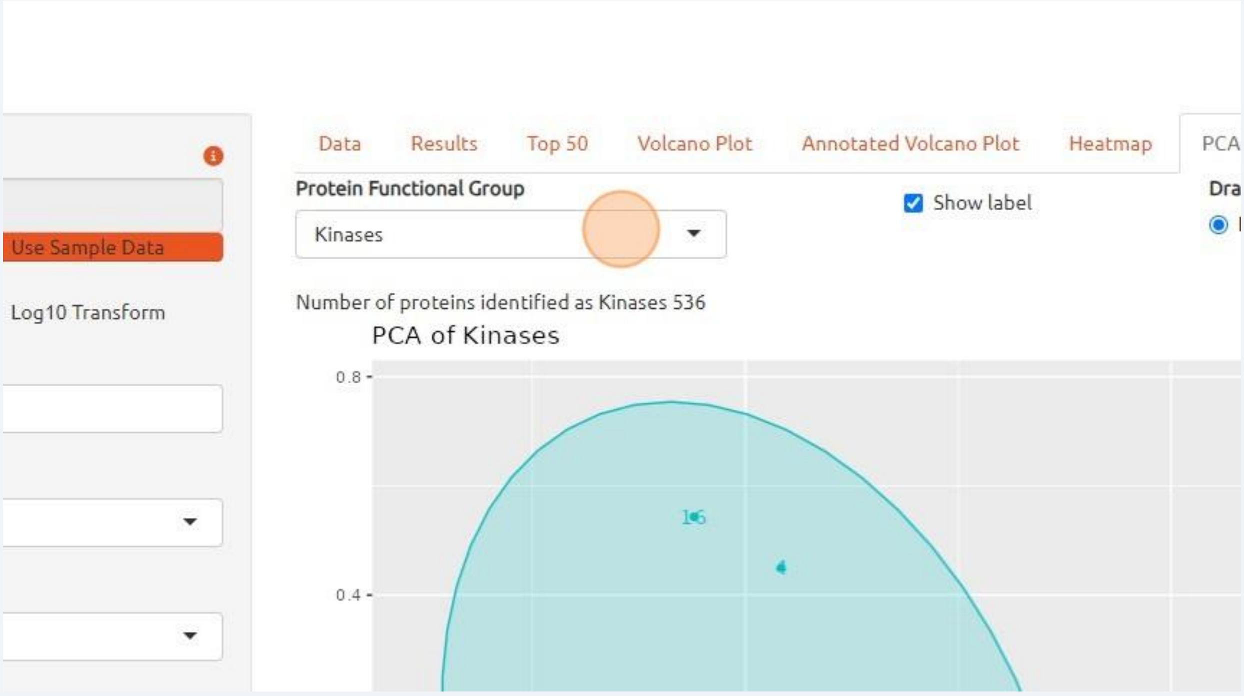

14 Filtered by kinases

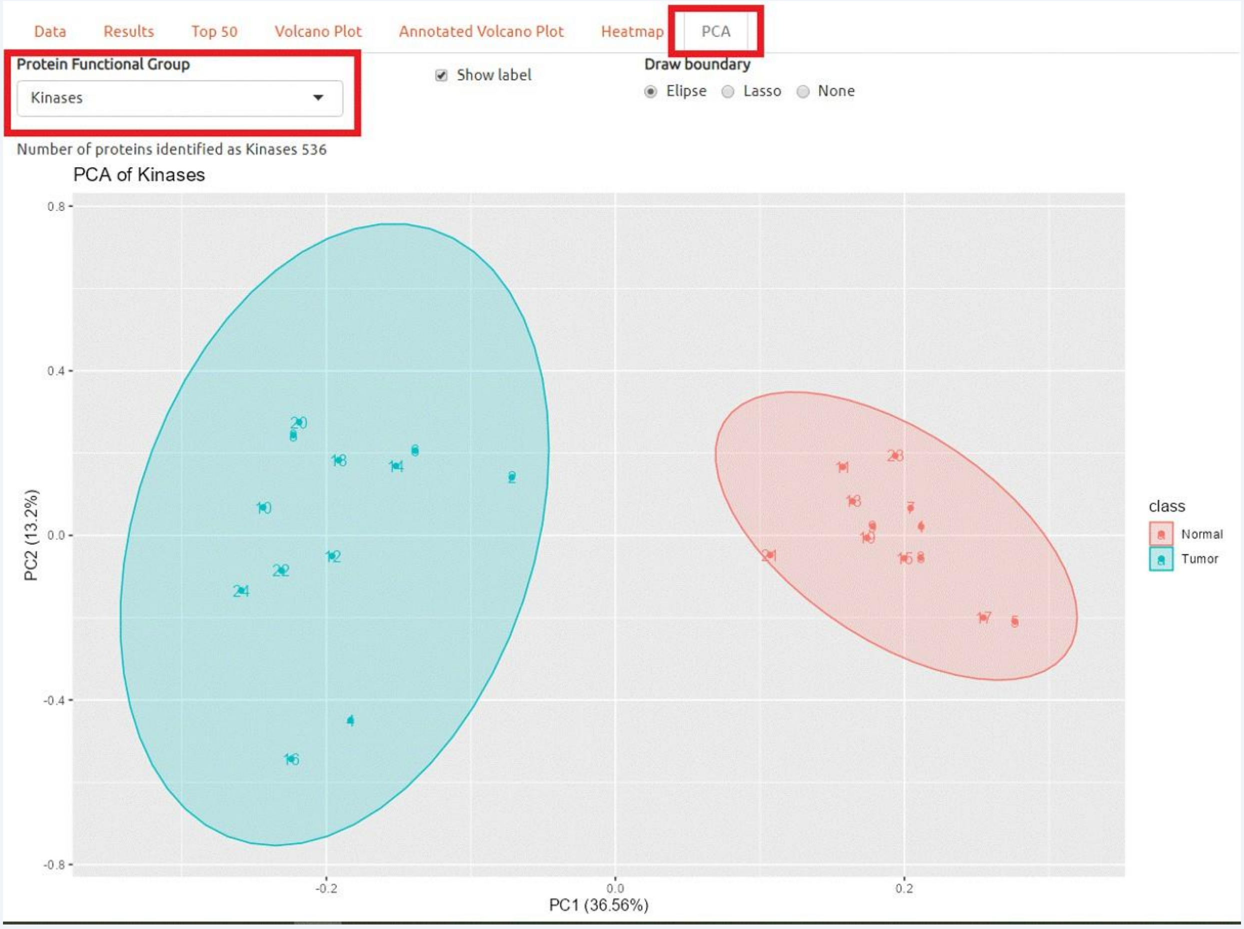

15 Click "Protoeases" to filter PCA with protoeases.

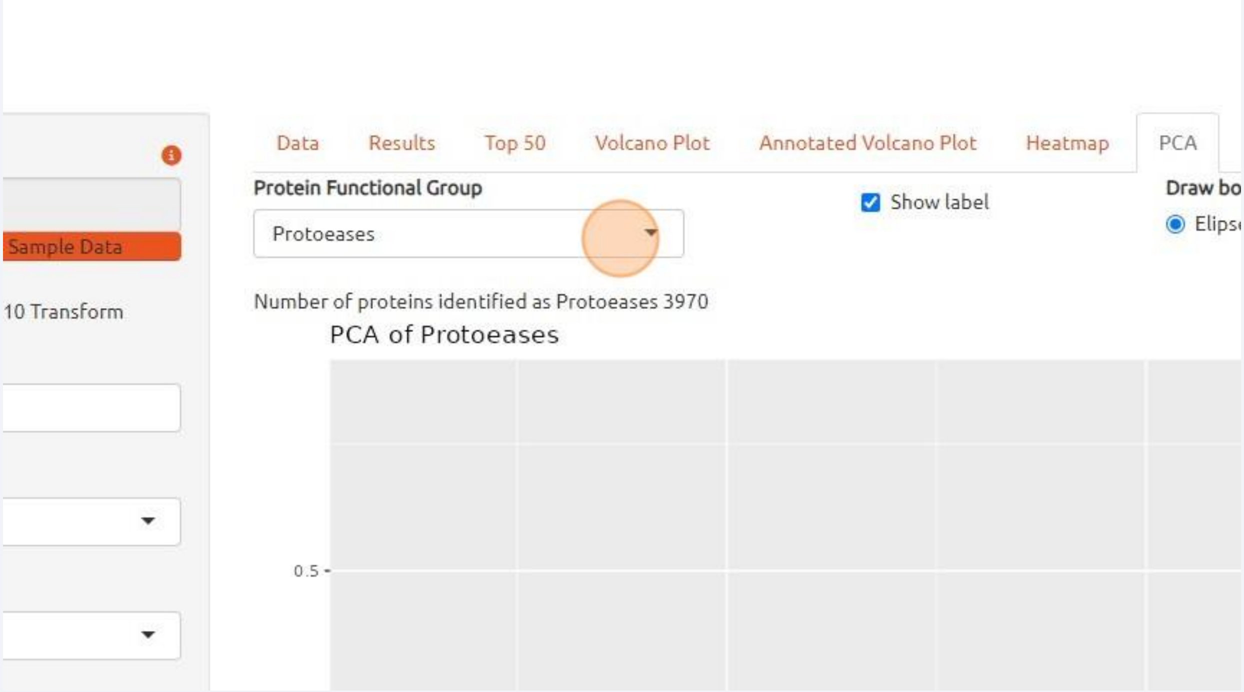

16 Filtered by protoeases

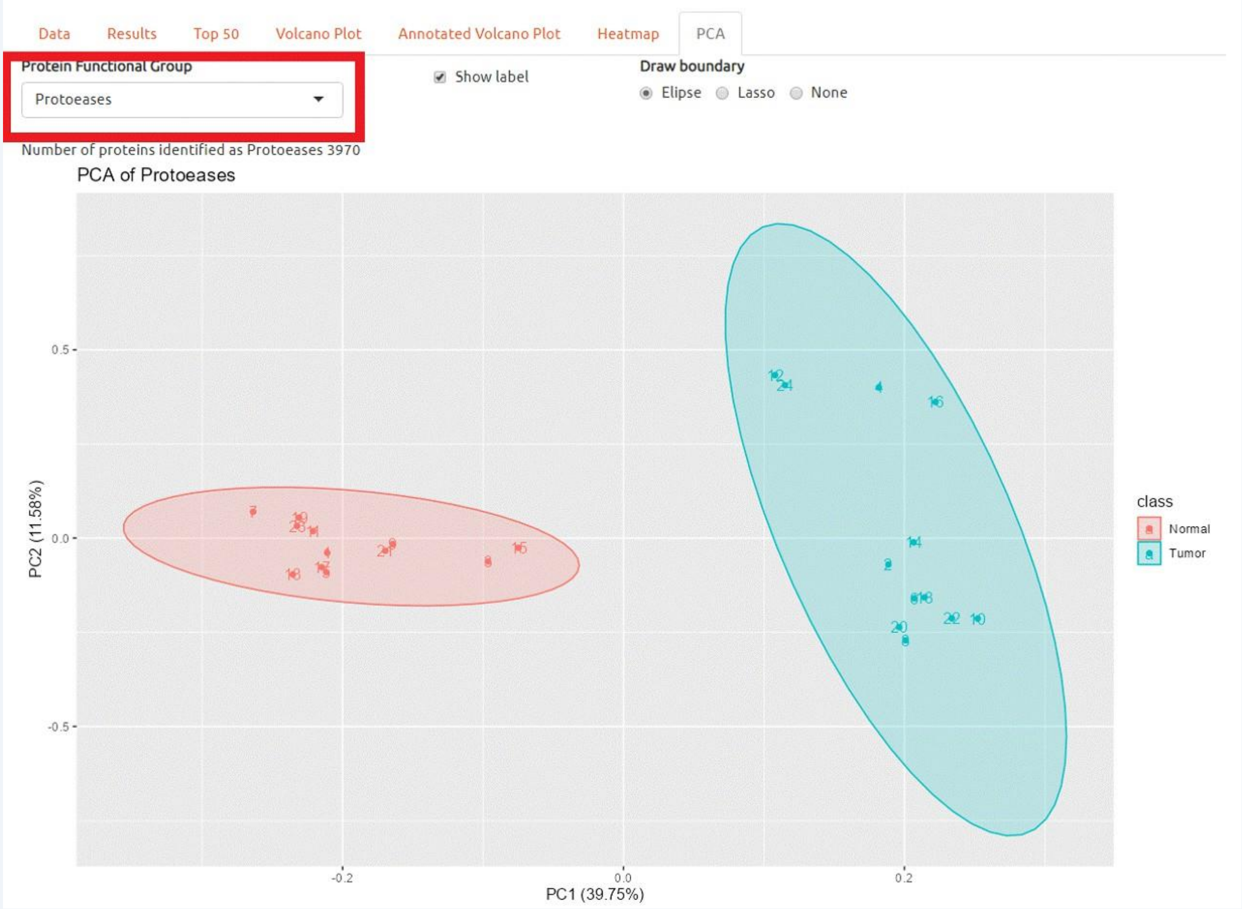

17 Click "Transcription Factors" to filter PCA with Transcription Factors.

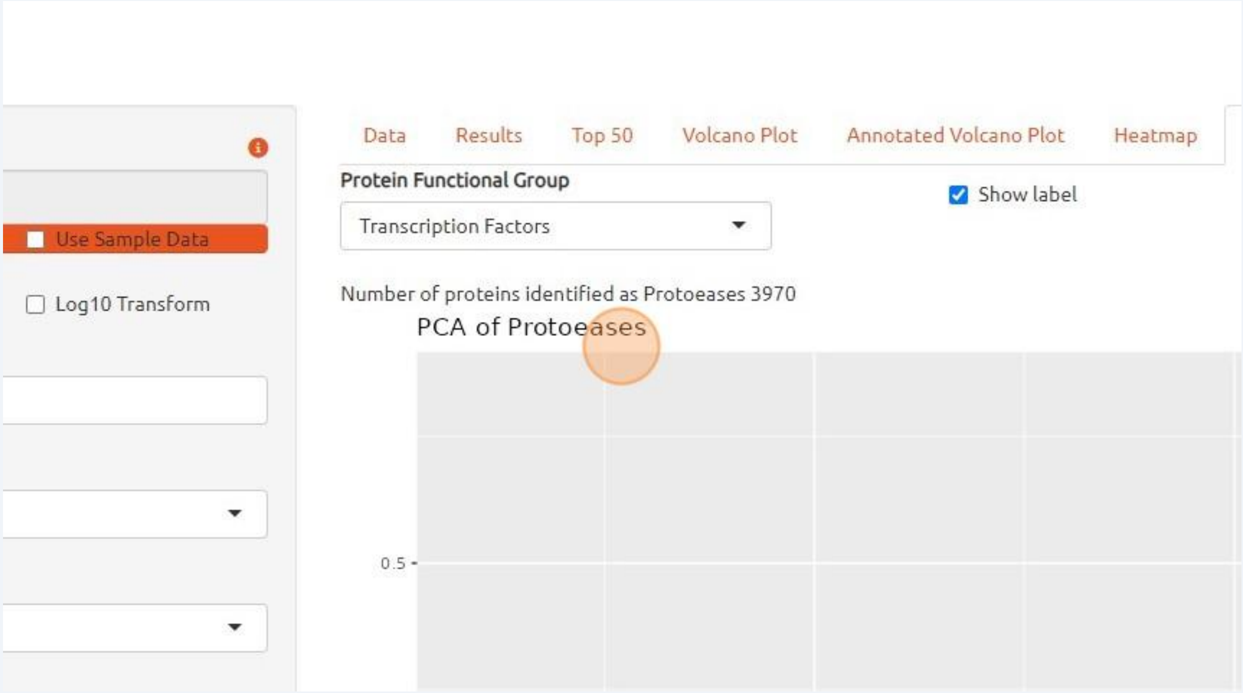

18 Filtered by transcription factors

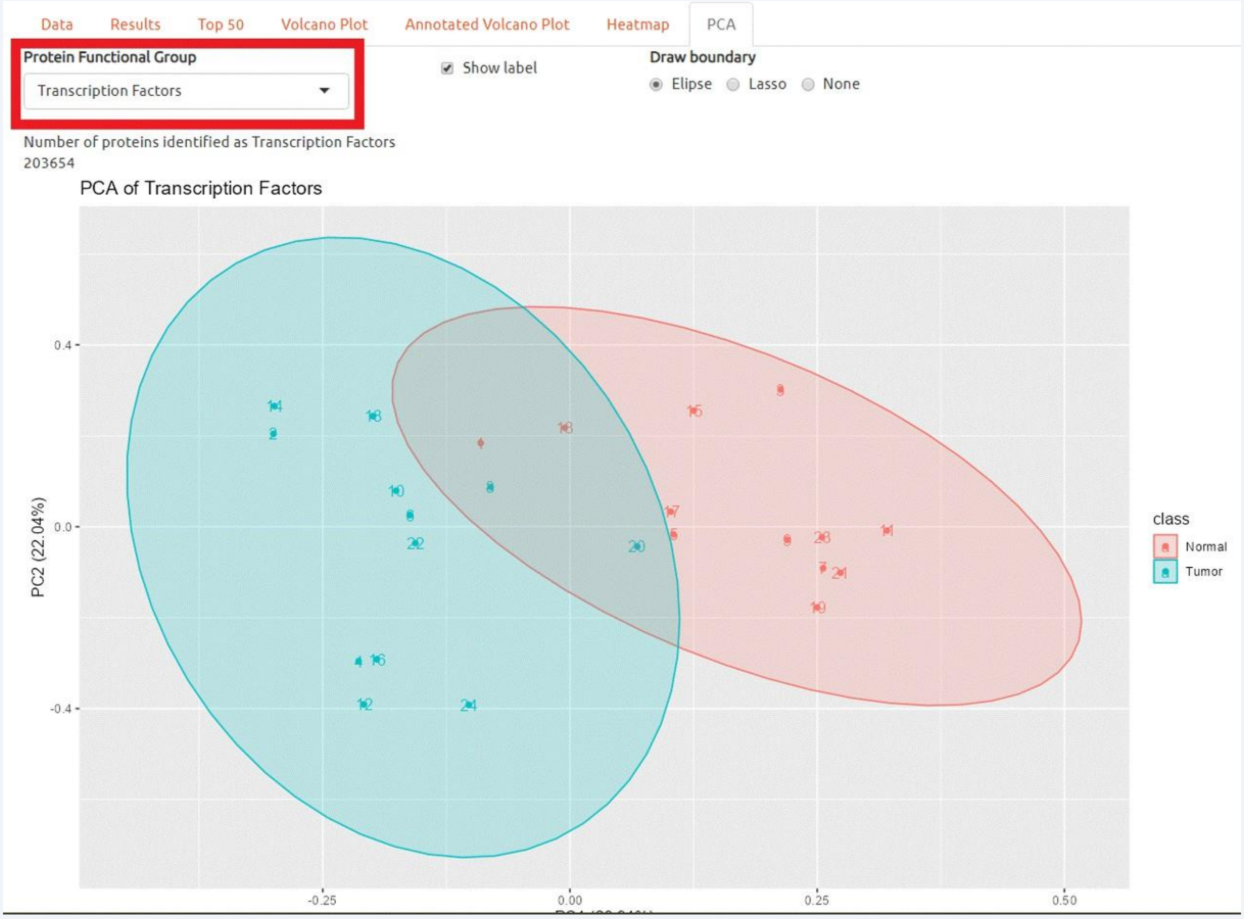

Step-by-step Guide to Perform Automated Differential Expression Analysis using FlexStatv1 Pipeline

Scribe

This feature facilitates combinatory differential expression analysis for datasets with more than two classes/conditions.

It systematically generates all possible pairwise comparisons, combines multiple classes/conditions, and presents detailed results for the differential expression analysis.

1 Navigate to **https://jglab.shinyapps.io/flexstatv1-pipeline-only/**

2 Go to the "Automated Combinatory Differential Expression" tab.

3 Click "Use Sample Data"

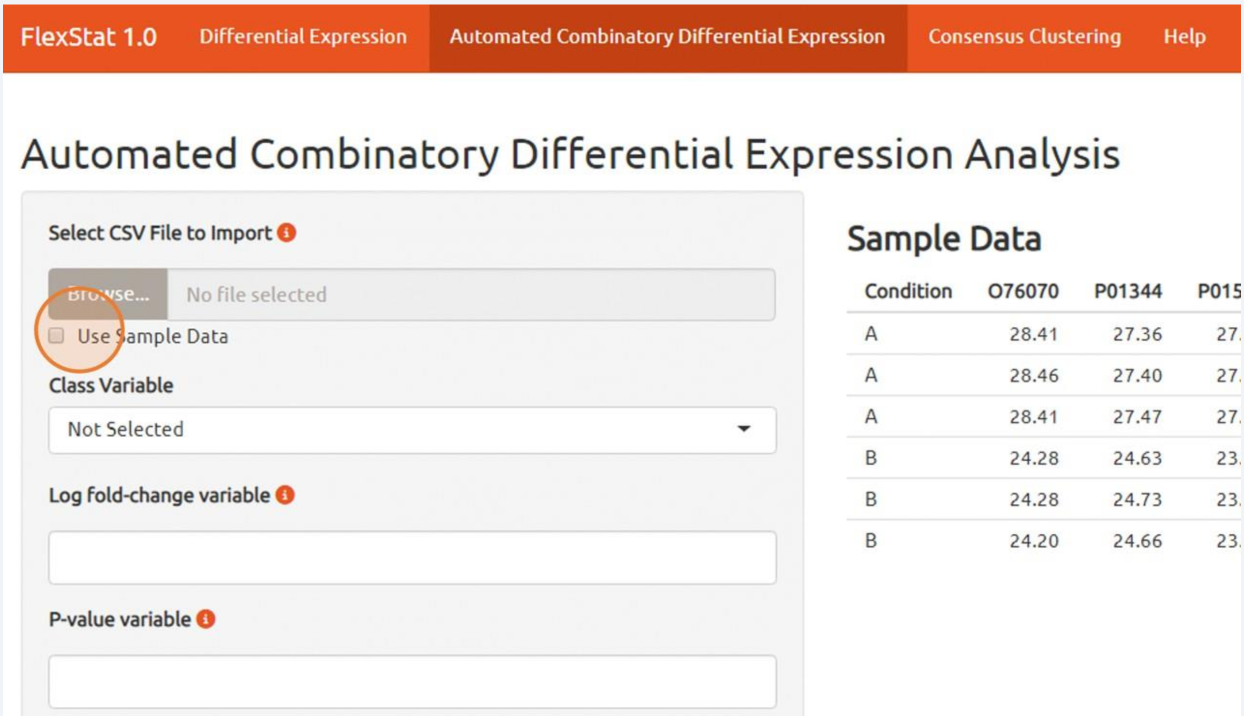

4 Click "Condition" as the Class variable and select correction for multiple comparisons.

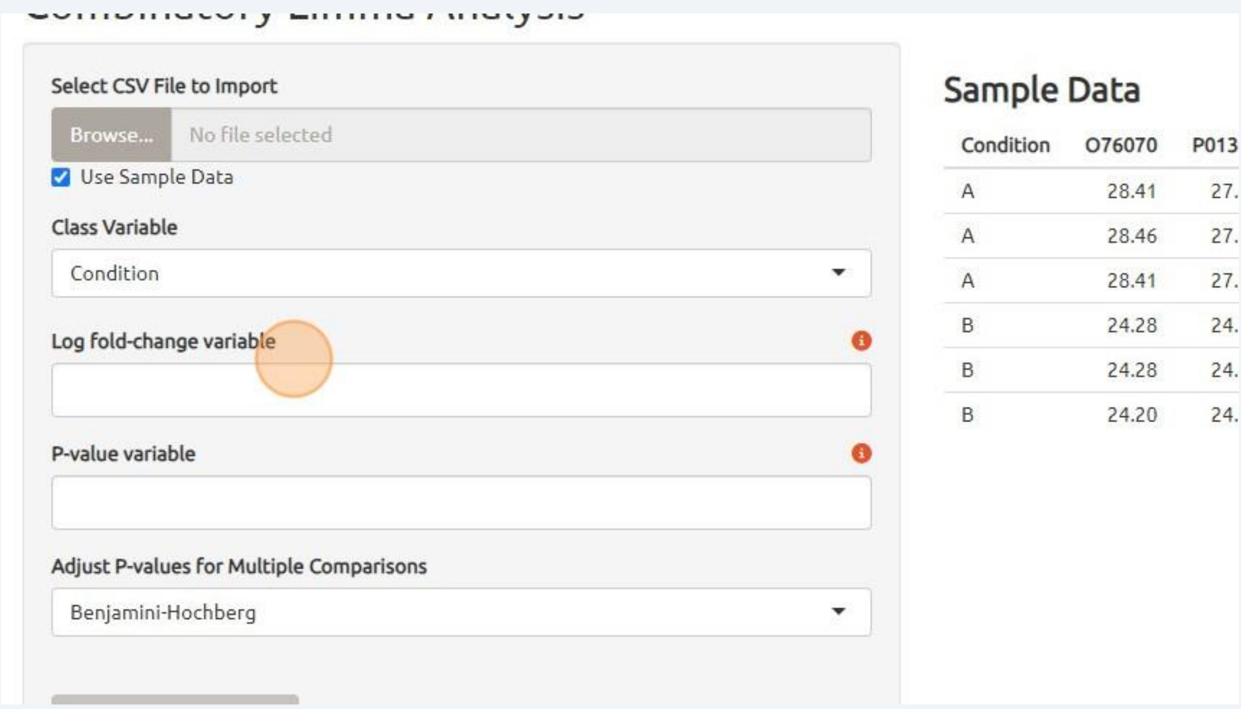

5 **[Optional]** Change log fold change and p-value cutoffs based on the research question at hand.

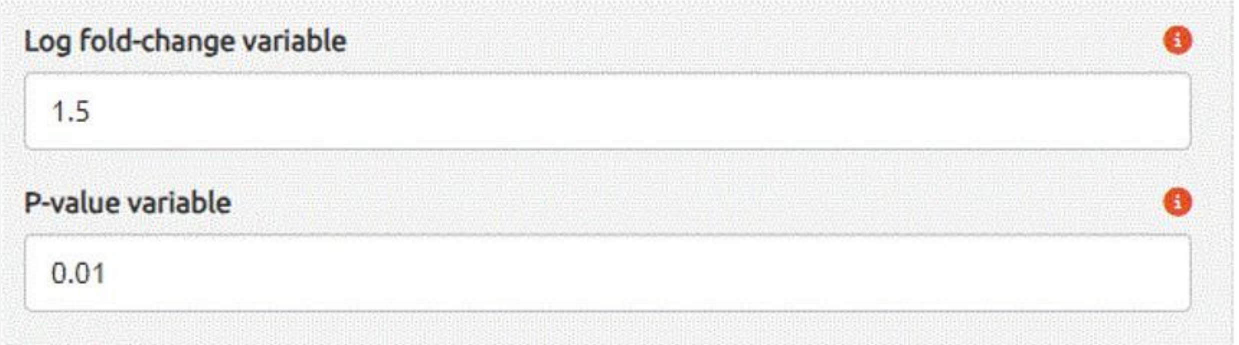

6 **[Optional]** Change the correction procedure for multiple comparison tests based on the research question at hand.

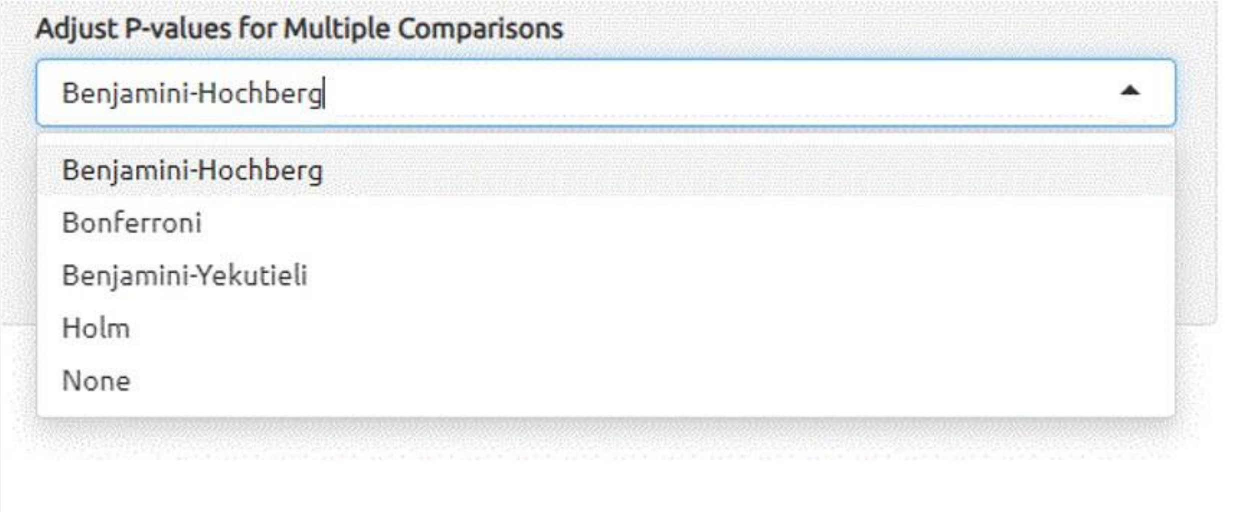

7 Click "Perform Auto Limma"

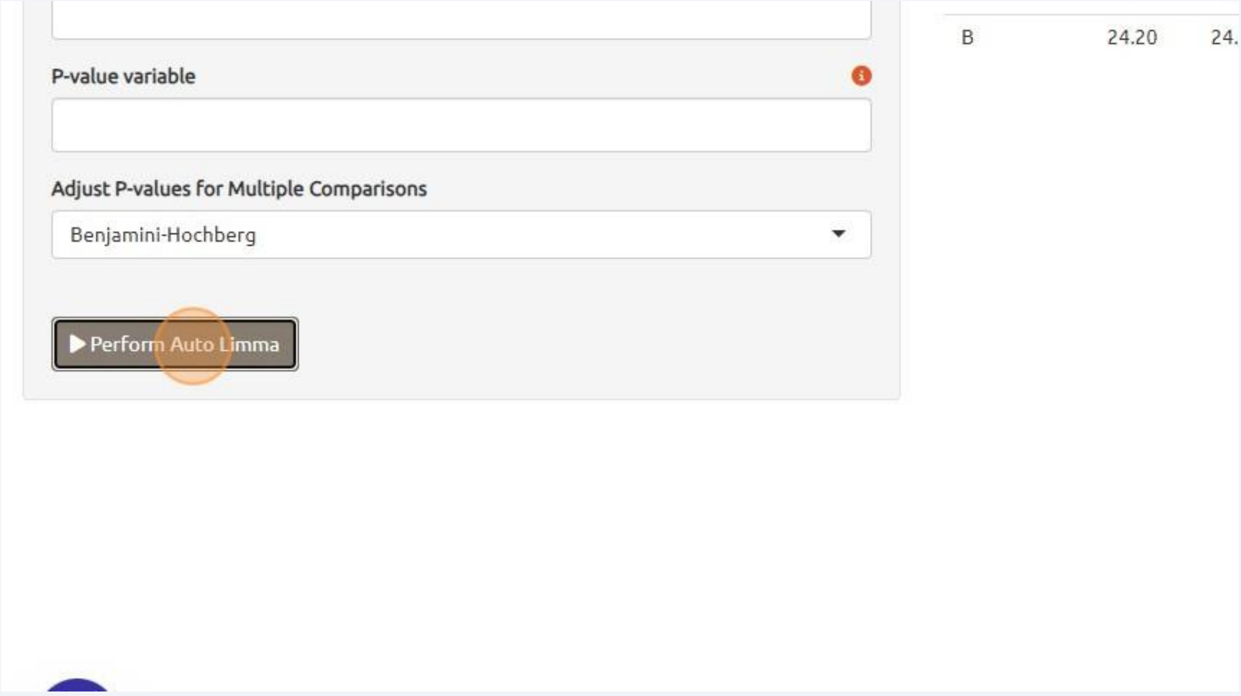

8 This will result in calculating all possible combinations ofthe classes.

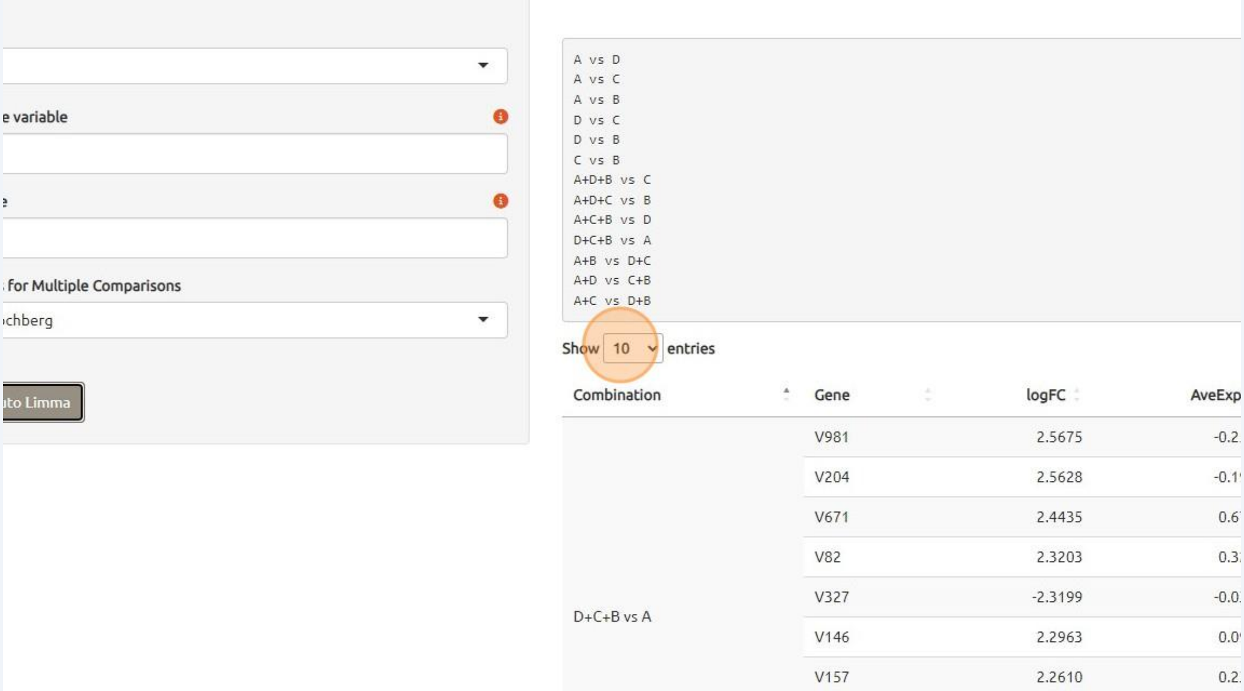

9 This will result in producing a table of differential expression results for each combination.

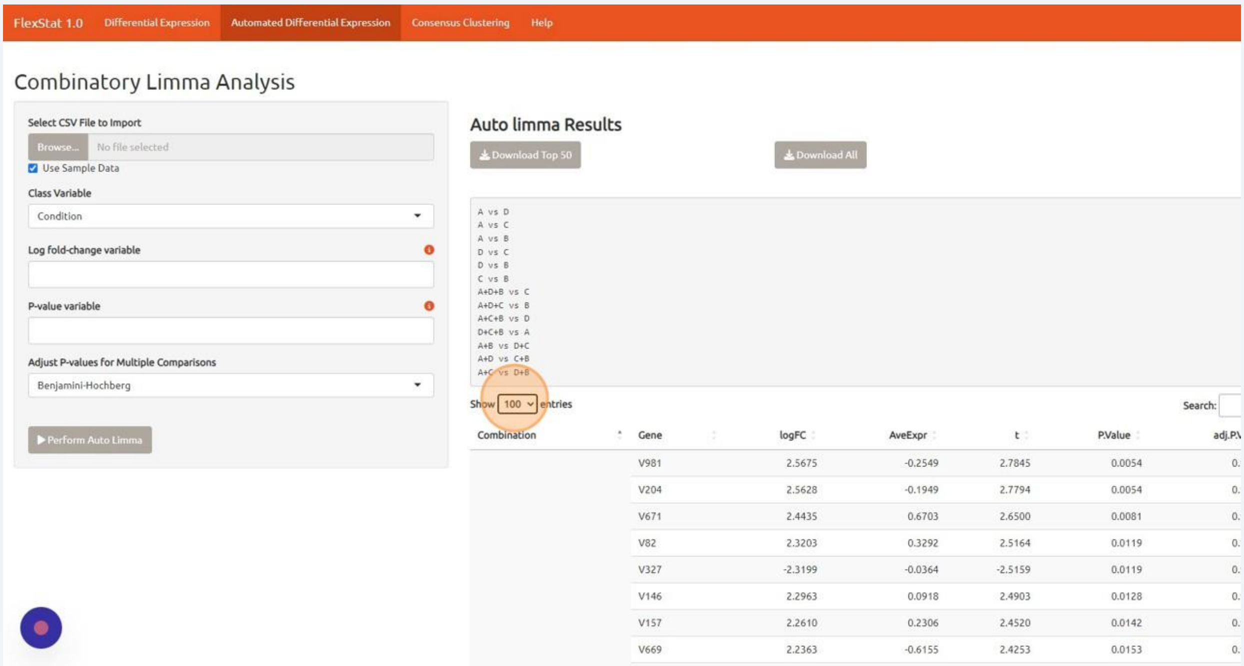

10 Navigate using page numbers to explore the results.

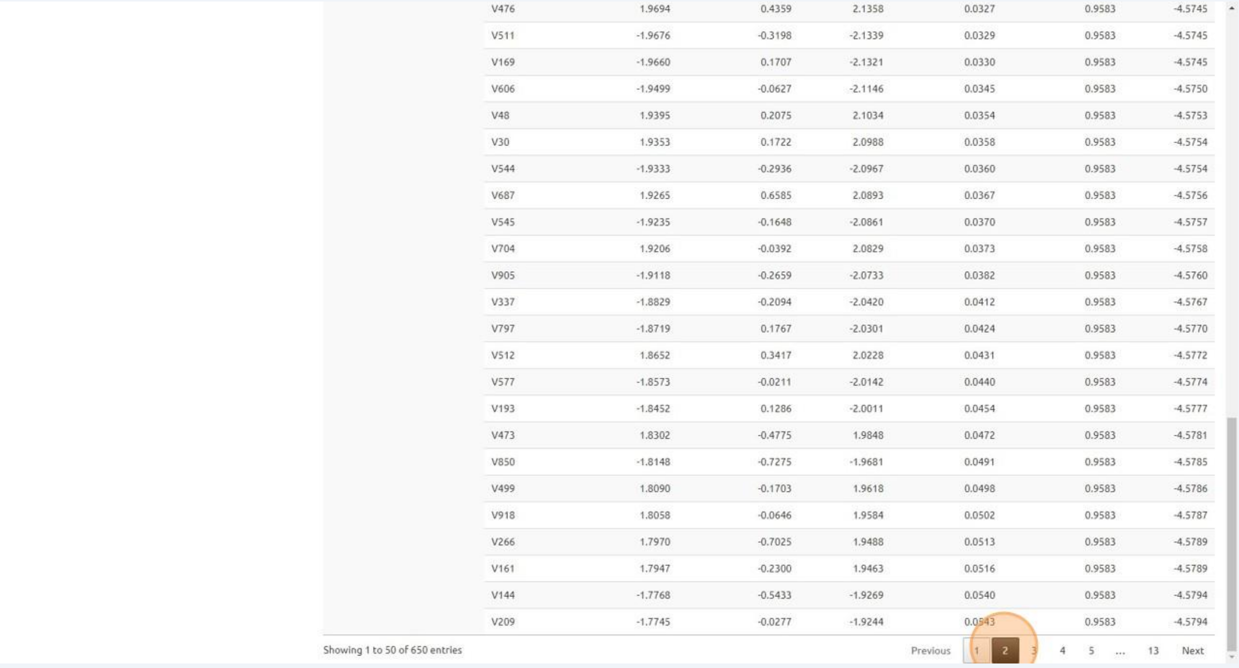

11 Click "Download Top 50" to download top 50 genes/proteins from each combination

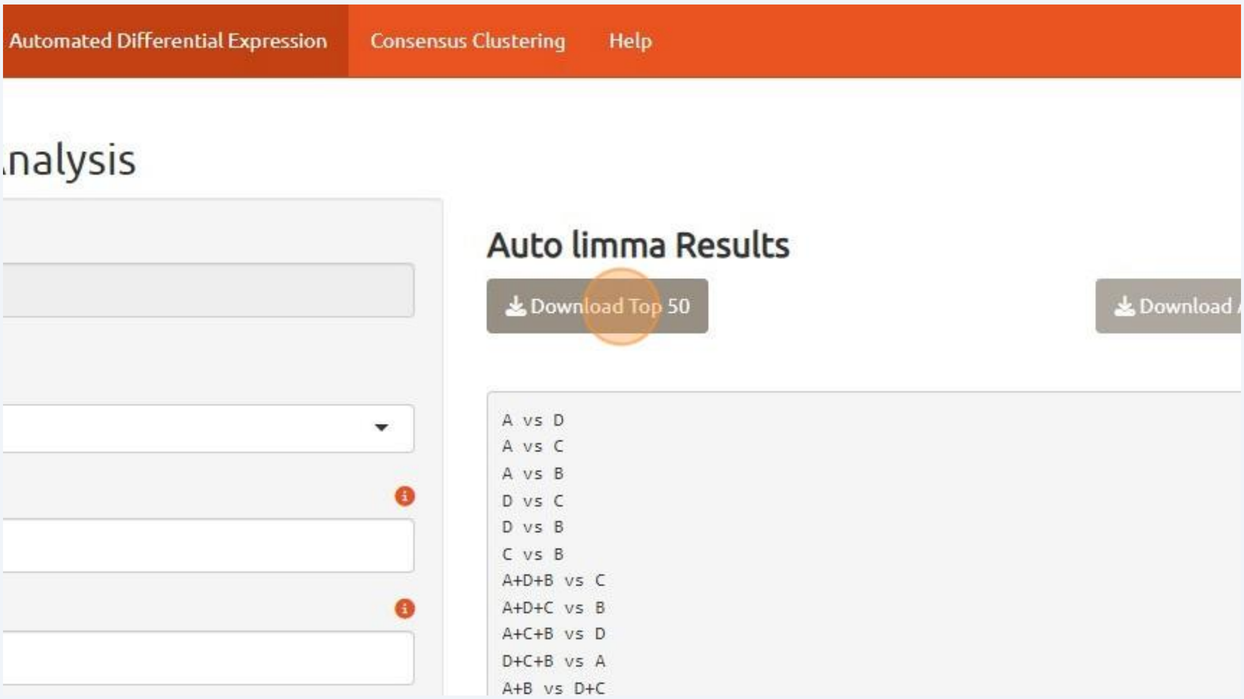

12 Downloaded excel files will be as follows. The first sheet will be an index explaining the subsequent sheets representing different combinations.

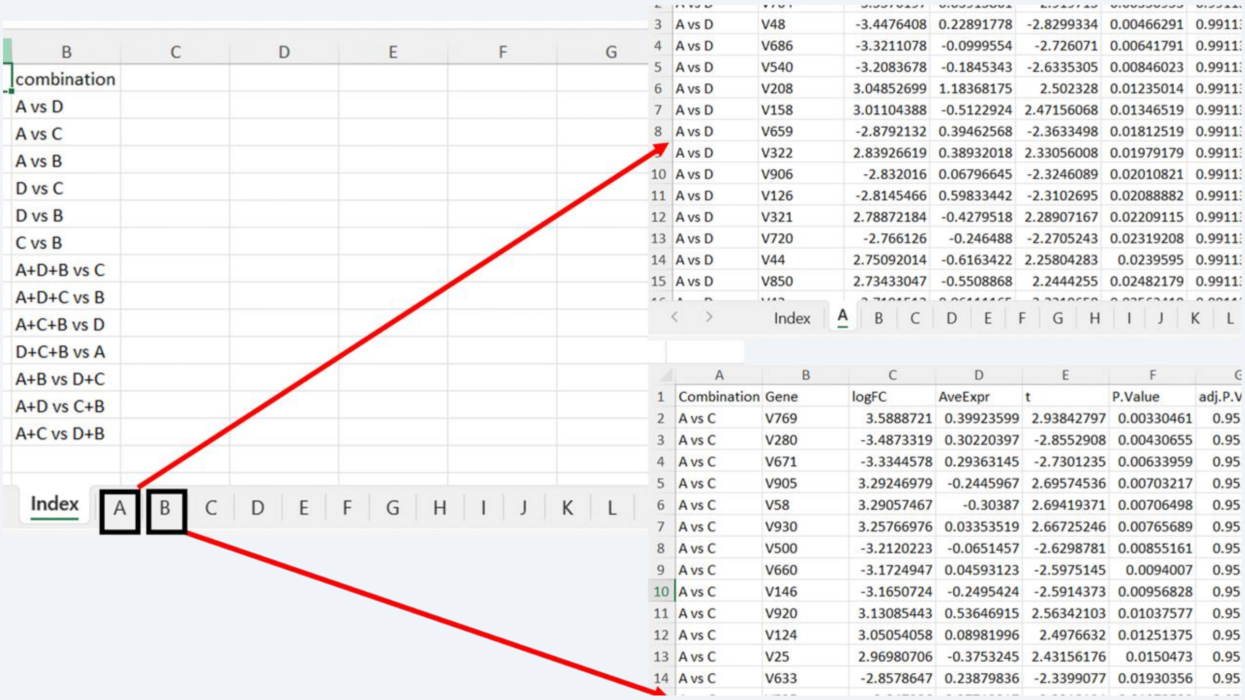

13 Click "Download All" to download all results.

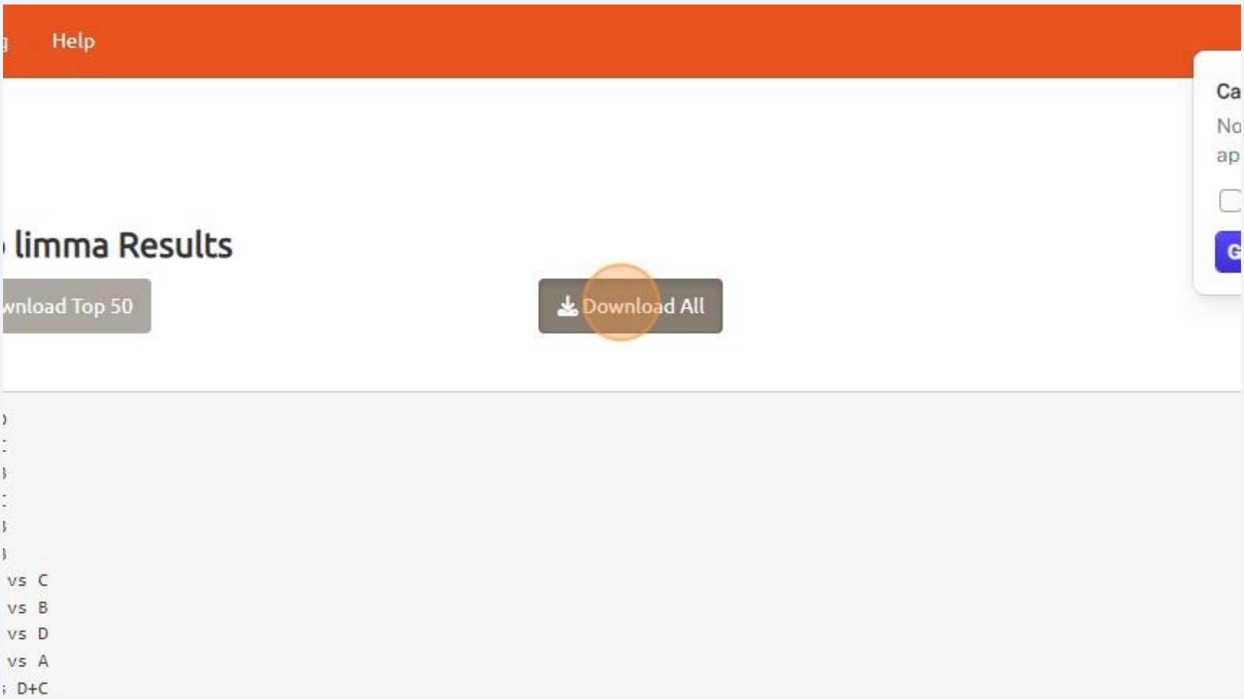

Step-by-step Guide to Perform Consensus Clustering using FlexStatv1 Pipeline

This feature enables consensus clustering of a expression profile by customizing the clustering algorithm, distance measurements, and linkages

1 Navigate to https://jglab.shinyapps.io/flexstatv1-pipeline-only/

2 Go to "Consensus Clustering" tab.

3 Click "Use Sample Data"

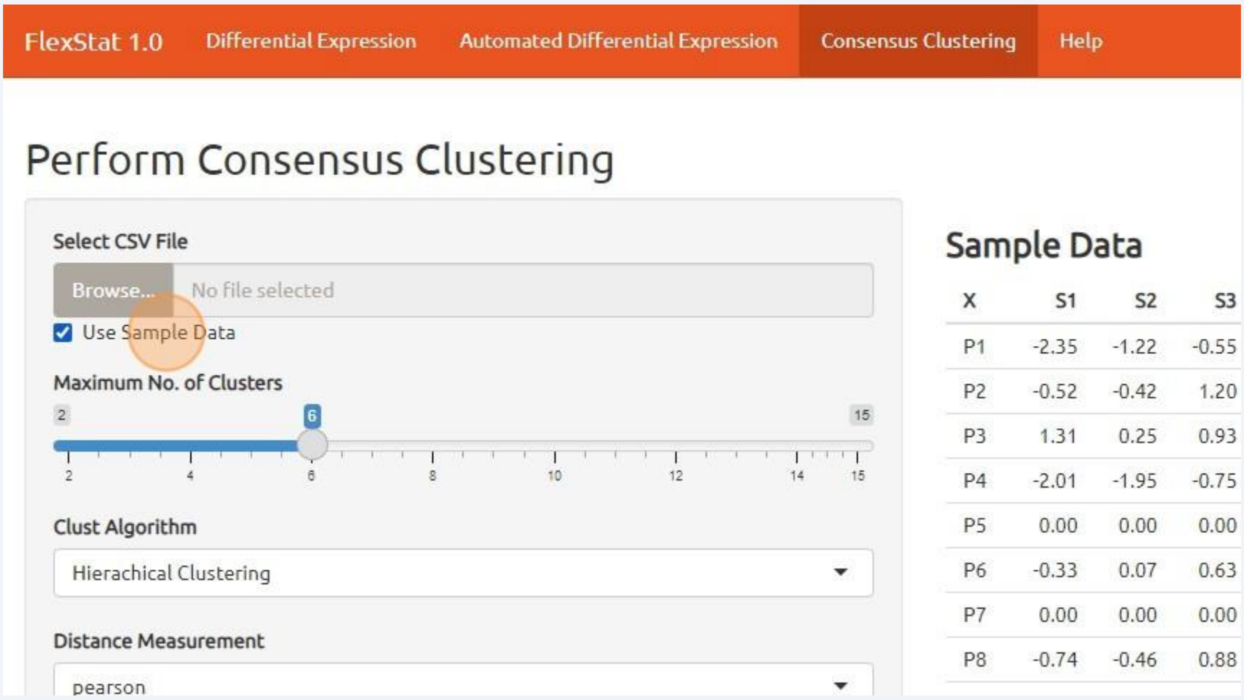

4 Select maximum number of clusters to generate the consensus clustering matrix.

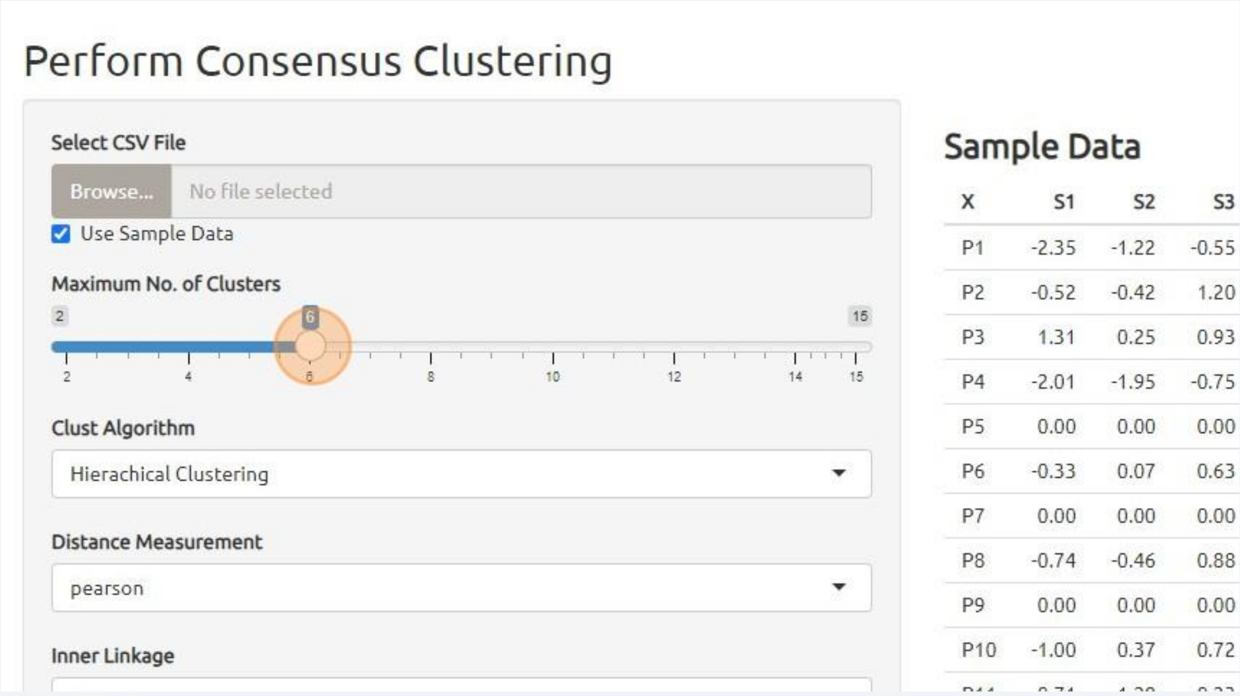

5 Select clustering algorithm.

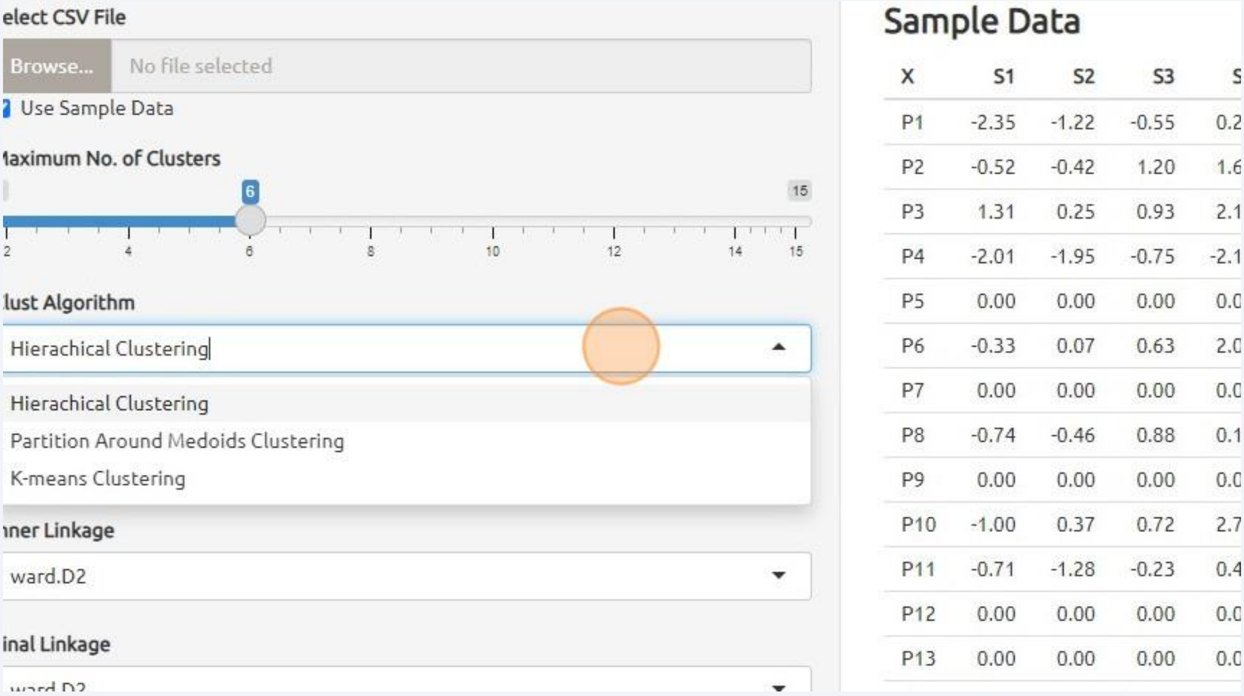

6 Select distance measurement to define clusters.

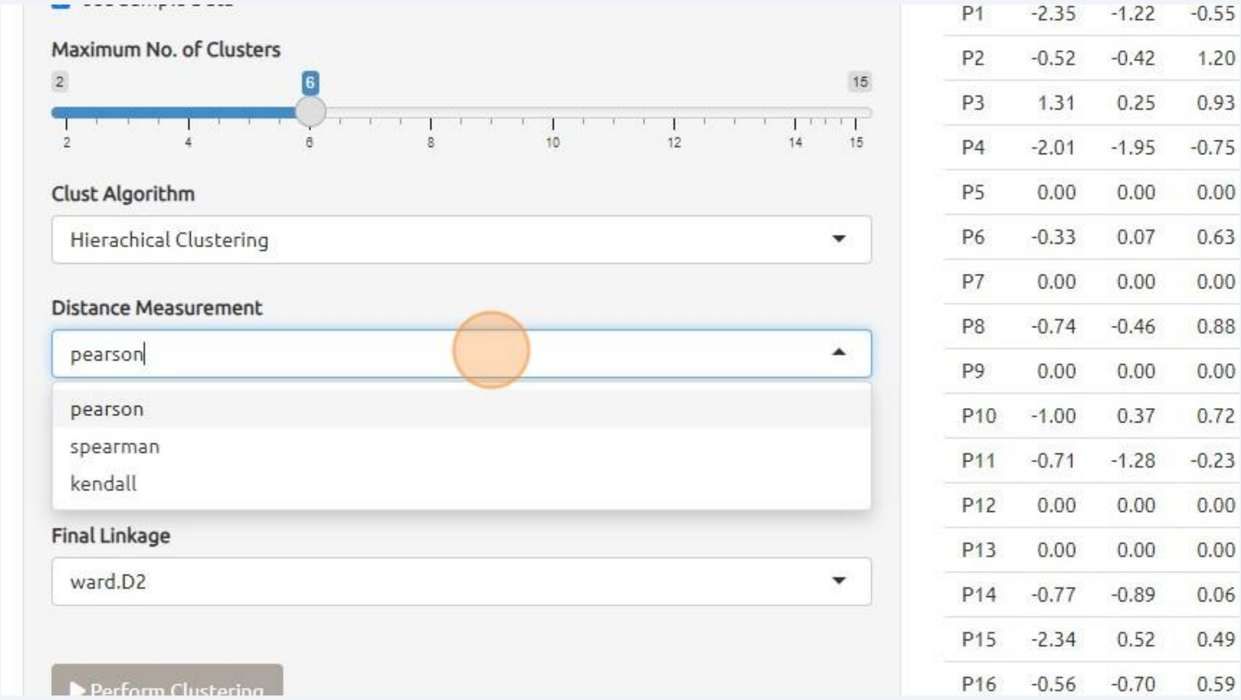

7 Select distance measurement to be used in iterative agglomerative clustering.

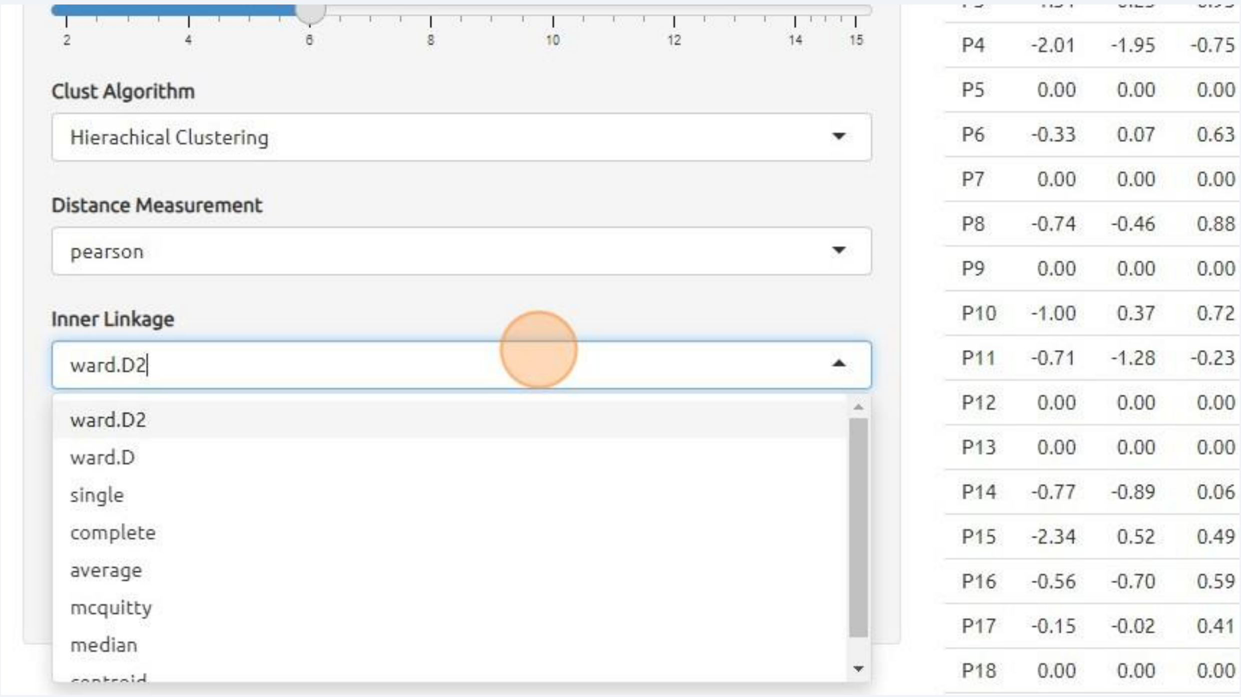

8 Select distance measurement to be used in the final agglomerative clustering.

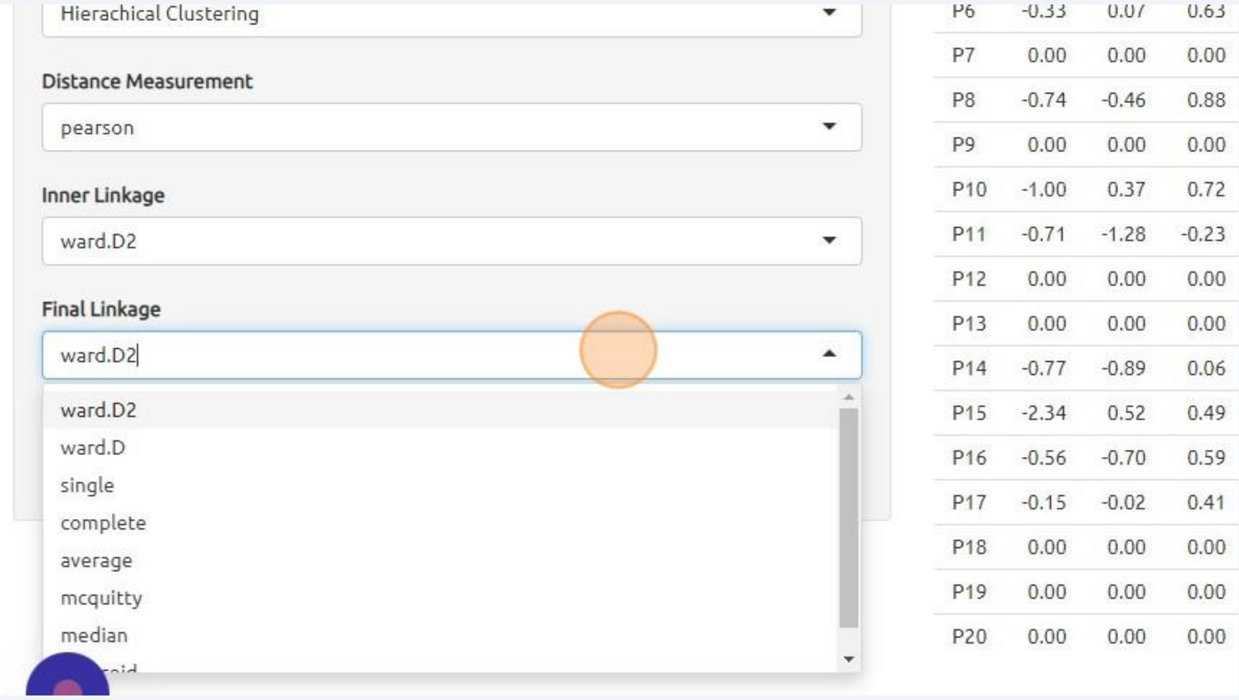

9 Click "Perform Clustering"

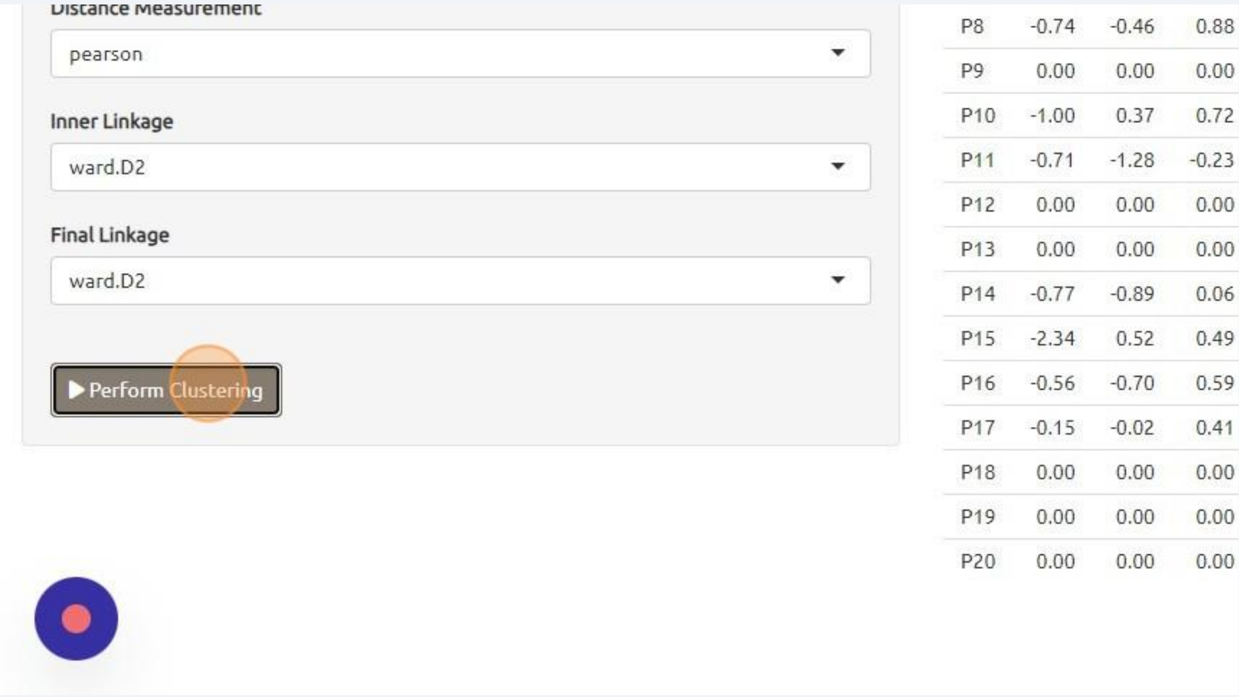

10 Explore the clustering results.

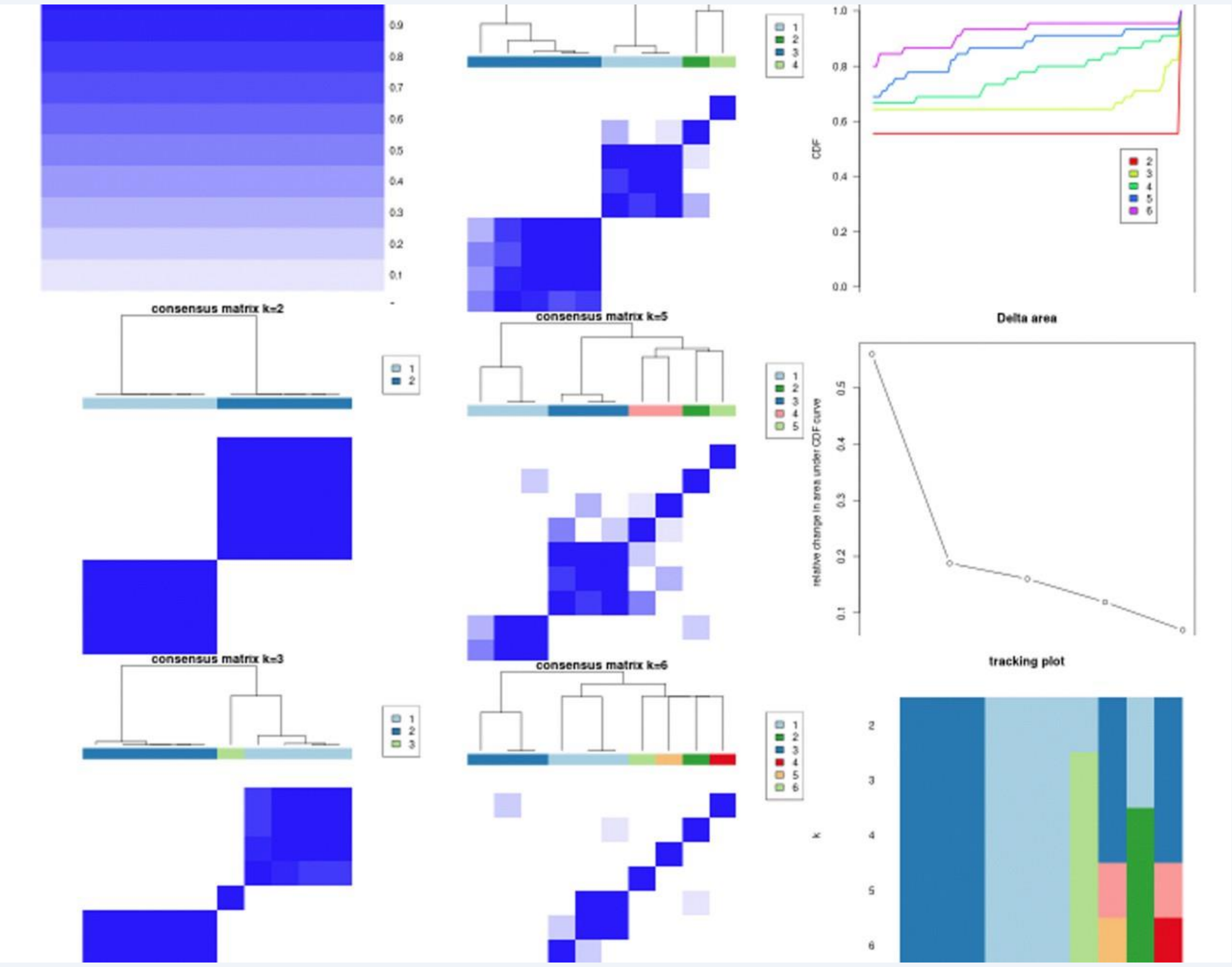

11 Click "download icon" to download the clustering results as a zip file.

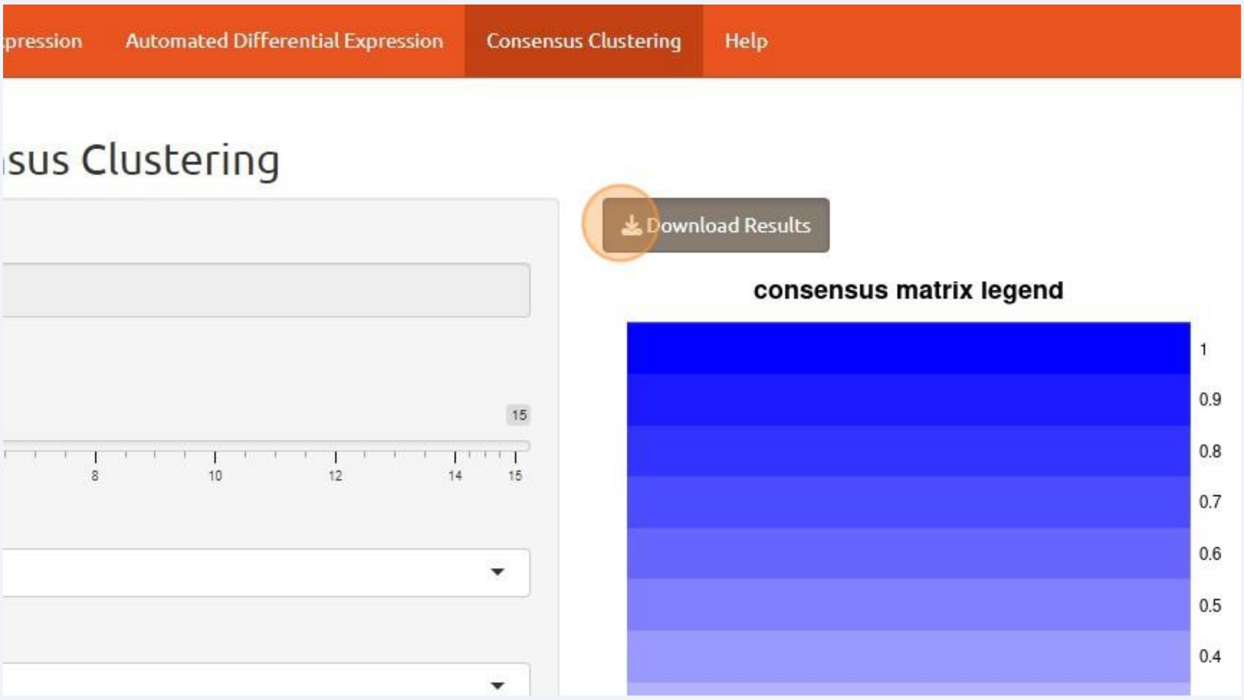

12 The zip file consists of clustering outcome images and an excel with excel file with cluster assignments.

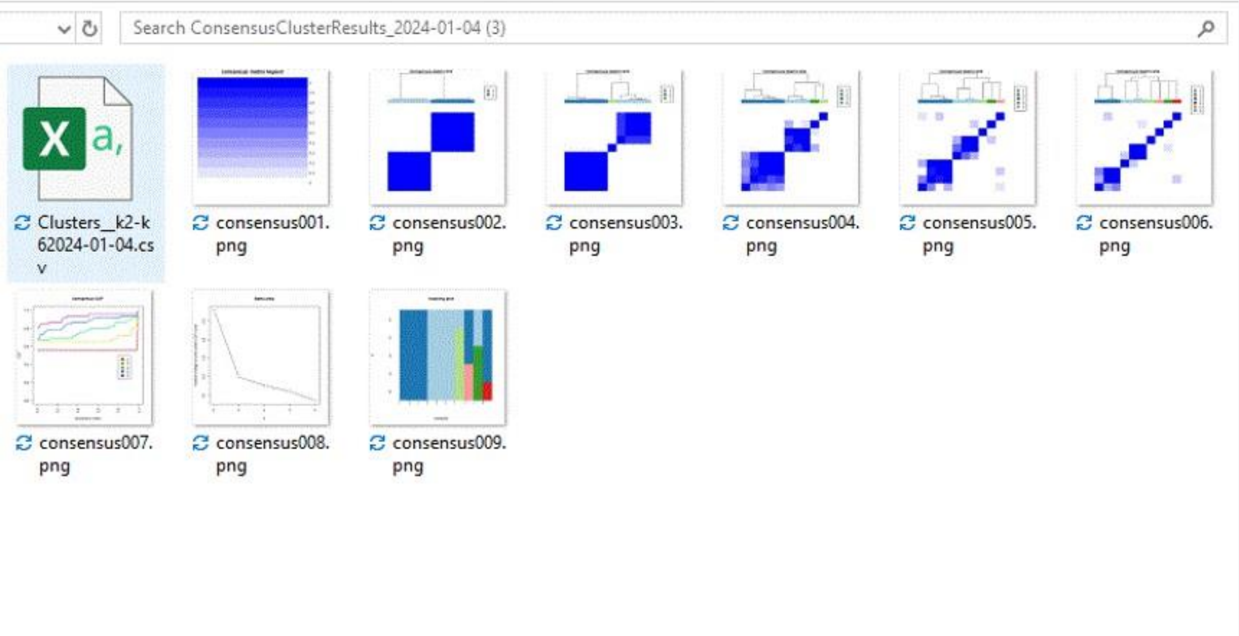

13 The cluster assignments matrix represents samples in rows and cluster number (i.e.1,2,3...) as columns.

	k2	k3	k4	k5	k6
S1	1	1	1	1	1
S2	1	1	2	2	2
S3	2	2	3	3	3
S4	2	2	3	3	4
S5	2	2	3	3	3
S6	1	1	1	1	1
S7	2	2	3	3	3
S8	2	2	3	4	5
S9	1	1	1	1	1
S10	1	3	4	5	6

Clusters__k2-k62024-01-04
